# Developmental factors drive the compartmentalized and discontinuous maturation of the small intestinal epithelium during the early postnatal period

**DOI:** 10.1371/journal.pbio.3003212

**Published:** 2026-08-03

**Authors:** Johannes Schöneich, Aline Dupont, Stefan Schlößer, Mingbo Cheng, Matthias A. Schmitz, Isabel Richter, Narasimha Murthy Keshava Prasad Gubbi, Kaiyi Zhang, Tiago Maié, Asmae Laouina, Sofia Jäverfelt, Fabian Imdahl, Christophe Toussaint, Antoine-Emmanuel Saliba, Christoph Kuppe, Oliver Pabst, Thaher Pelaseyed, Ivan G. Costa, Mathias W. Hornef

**Affiliations:** 1 Institute of Medical Microbiology, RWTH Aachen University Hospital, Aachen, Germany; 2 Institute for Computational Genomics, RWTH Aachen University Hospital, Aachen, Germany; 3 Institute for Molecular Medicine, RWTH Aachen University Hospital, Aachen, Germany; 4 Department of Medical Biochemistry and Cell Biology, Institute of Biomedicine, University of Gothenburg, Gothenburg, Sweden; 5 Helmholtz Institute for RNA-based Infection Research (HIRI), Helmholtz-Center for Infection Research (HZI), Würzburg, Germany; 6 Institute of Molecular Infection Biology Faculty of Medicine, University of Würzburg, Würzburg, Germany; 7 Cluster for Nucleic Acid Sciences and Technologies - NUCLEATE, Würzburg, Germany; 8 Department of Nephrology and Hypertension, Rheumatology and Immunology (Medical Clinic II), Medical Faculty RWTH Aachen, Aachen, Germany; 9 Center of Excellence in Nephrology, University Hospitals Aachen Cologne Dusseldorf, Aachen, Germany; Center for Genome Engineering, KOREA, REPUBLIC OF

## Abstract

The temporal, spatial, and cellular diversity of the small intestinal epithelium during the postnatal period, a critical time window that accompanies the transition from placental energy supply to enteral feeding, and the establishment of the enteric microbiota and postnatal immune maturation, has not been systematically investigated. Here, we used laser capture microdissection and bulk RNA-Seq, proteomics, and single cell RNA-Seq to analyze the total, organ site, crypt- and villus-specific intestinal epithelium of specific pathogen-free, germ-free, and *Salmonella*-infected mice during the postnatal period. We identified key temporal and organ-site specific expression patterns that revealed a weak effect of the microbiota but strong influence of developmental regulators during early life. We also determined age-dependent signaling pathway and transcription factor activity and characterized age- and cell type-specific developmental trajectories revealing a distinct compartmentalized maturation process along the proximal-to-distal length and crypt-villus axis and a discontinuous appearance of goblet/Paneth cell and absorptive enterocyte transcriptional profiles. Finally, we described the cell type-specific response to neonatal enteric infection. Taken together, our findings identify the epithelium as an integral element in the maturation of postnatal mucosal tissues and in the establishment of host-microbe homeostasis.

## Abstract 2

During the postnatal period, the small intestinal epithelium transitions from placental energy supply to enteral feeding. This study systematically analyses the temporal, spatial and cellular diversity during the maturation of murine postnatal mucosal tissues and the establishment of host-microbe homeostasis.

## Introduction

The small intestinal epithelium facilitates the digestion and absorption of dietary nutrients, forms a physicochemical mucosal barrier that restricts the luminal enteric microbiota, and represents the first line of defense in the event of infection. The wide range of functions of the small intestine is reflected by the broad diversity of epithelial cell types such as stem and transit amplifying (TA) cells, absorptive enterocytes, secretory Paneth, goblet, tuft and enteroendocrine cells (EEC), and M cells, all in turn composed of different subtypes and differentiation stages [1–5]. This cellular diversity is regulated by a complex network of transcriptional and nontranscriptional endogenous factors [6–13]. In addition, intestinal epithelial cells actively interact with the microbiota and the underlying immune and stroma cells [14–19]. While intestinal epithelial cell heterogeneity has been characterized in adult mice, as well as in the pediatric and adult human host [2,3,20,21], much less is known about the early postnatal period [22]. This period, however, is particularly critical since it facilitates the transition from placental energy supply to enteral feeding and the concomitant establishment of the enteric microbiota and maturation of the mucosal immune system, critical to the maintenance of the host-microbe homeostasis. In addition, the neonatal intestinal epithelium differs significantly from that of the adult host [1,20]. Crypts and Paneth cells both appear postnatally only around the second week of life [23,24]. In the absence of crypts, it also lacks defined TA cells, continuous epithelial cell proliferation, and established crypt-villus migration [25]. On the other hand, fetal-type enterocytes that engulf luminal material for intraepithelial processing and the efferocytosis of damaged epithelial cells by neighboring enterocytes are restricted to the neonatal period [26,27]. Significant differences in the transcription of metabolic enzymes, differentiation factors, innate immune sensing and antimicrobial host defense molecules have been observed between neonatal and adult mice [6,7,28–32]. These differences, controlled in part by master regulators of developmental epithelial gene expression such as BLIMP1 (encoded by *Prdm1*) or MAFB, may also result from microbial signals [6–8,14].

**Fig 1:**
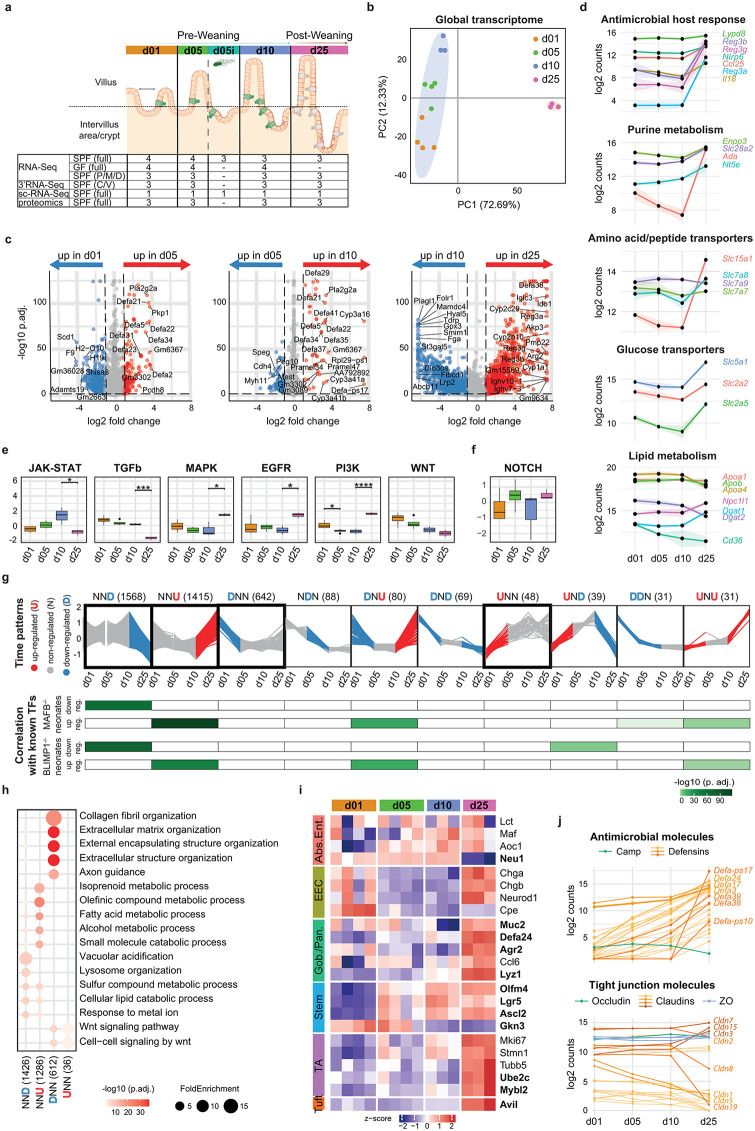
The global intestinal epithelial transcriptome during postnatal development. **(a)** Schematic representation of the development of the murine small intestine during the early postnatal period (top) and table summarizing the different samples (number of biological replicates indicated) analyzed in this study (bottom). P: proximal, M: medial, D: distal, C: crypt, V: villus. Created in BioRender. Schleibach, M. (2026) https://BioRender.com/1c0dcwd. **(b)** PCA plot representing the full small intestinal epithelial transcriptome of 1-day-old (orange, *n* = 4), 5-day-old (green, *n* = 4), 10-day-old (blue, *n* = 3) and 25-day-old (pink, *n* = 3) mice. Ellipses were estimated using the Khachiyan algorithm and show the distribution of the pre-weaning (light blue) and post-weaning (light pink) samples. **(c)** Volcano plots showing differential gene expression between 1- and 5-day-old (left), 5- and 10-day-old (middle), and 10- and 25-day-old (right) samples. Blue and red dots indicate genes statistically (*P*adj < 0.05) upregulated (|log_2_FC| > 1) in the younger and older animals, respectively. Gray dots indicate nonsignificantly and/or nondifferentially regulated genes. **(d)** Temporal expression pattern of selected genes encoding antimicrobial, metabolic, and transport molecules in 1-, 5-, 10-, and 25-day-old SPF mice [3]. Median (lines) and median absolute deviation (MAD) (patches). **(e, f)** Box plots showing PROGENy pathway analysis (e) and the NOTCH pathway analysis (f) at the different ages. Color code as in Fig 1b. ANOVA with Tukey-HSD post-hoc-test. **(g)** 10 most abundant temporal postnatal gene expression patterns as identified by temporal DE analysis (U, upregulated, red; N, nonregulated, gray; D, downregulated, blue) and their association with reported BLIMP/MAFB gene regulation. The number of DE genes for each pattern is indicated in brackets. **(h)** Dot plot showing the top 5 enriched GO terms (by adjusted *p*-value) for the DE genes identified as belonging to the NND, NNU, DNN and UNN expression patterns. The number of DE genes associated with a GO term for each pattern is indicated in brackets. **(i)** Heatmap depicting the expression level of known marker genes for absorptive enterocytes (Abs.Ent.), enteroendocrine cells (EEC), goblet/Paneth (Gob./Pan.) cells, stem cells, transit amplifying (TA) cells and tuft cells. Bold names represent genes that were identified as DE between at least 2 consecutive time points. **(j)** Temporal expression (counts) of genes encoding for known antimicrobial (top panel) and tight junction molecules (bottom panel). Underlying data for panels b, c, d, e, f, i and j can be found in S1 Data. Underlying data for panels g and h can be found in S2 Data. Dataset available under GSE283495.

The aim of the present study was to characterize the multidimensional changes of the small intestinal epithelium during the neonate-to-adult transition. This period of life has attracted particular attention due to the strongly enhanced infection-associated mortality. Furthermore, epidemiological and experimental evidence has suggested that exposure to exogenous factors during this early period can have lasting consequences and determine long-term health [33,34]. State-of-the-art methods were used to characterize the global, organ-, site- and cell type-specific transcriptional and/or proteomic profile of the gut epithelium of healthy specific pathogen-free (SPF) and germ-free (GF) mice at different time points after birth as well as mice with neonatal enteric infection (Fig 1a). Our results represent an important new resource for the community and expand our understanding of the age-dependent changes occurring during this transitional period. Characterization of the influence of environmental factors may help to identify new strategies to promote health at early age and reduce childhood mortality.

## Materials and methods

### Ethics statement

All animal work was performed according to the regulation specified by the Federation for Laboratory Animal Science Associations (FELASA) and the German Society of Laboratory Animal Science (GV-SOLAS, http://www.gv-solas.de). Experimental animal work was approved by the North Rhine-Westphalia Office of Nature, Environment and Consumer Protection (LAVE, approvals 81-02.04.2017.A397).

### Animals

C57BL/6J SPF mice were bred and maintained under SPF conditions in ventilated cages, with 12-hour light/dark cycles, and ad libitum access to food and water at the Institute of Laboratory Animal Science at the RWTH University Hospital Aachen. GF mice were generously provided by Tom Clavel and Susan A. Jennings (RWTH University Hospital, Aachen). 1-day-old C57BL/6J SPF newborn mice were orally infected with 1µl PBS containing 100–300 CFU of log-phase *S.* Typhimurium (ATCC14028). At 4 days post-infection, all animals were sacrificed by decapitation and their small intestines removed and processed as described below.

### Sample preparation for bulk RNA-Seq and proteomics

Intestinal epithelial cells were isolated as previously described [28]. Briefly, full small intestines or parts of the small intestines (the proximal part corresponding to the proximal 5% to 20% of the full small intestine, the medial part to the 37.5% to 62.5% and the distal part to the 80% to 95%) were cut into small pieces, incubated at 37 °C in 30 mM EDTA for 10 min and shaken for 20 s. Detached epithelial cells were then filtered through a 100µm cell strainer, washed with PBS and pelleted by centrifugation. Pellets were then either resuspended in TRIzol reagent (RNA-Seq) or snap-frozen in liquid nitrogen (proteomics) and stored at −80 °C.

### Bulk RNA-Seq

RNA was subsequently isolated following manufacturer’s instructions and cDNA libraries were prepared using NEBNext ultra II directional RNA library prep kit for Illumina with the NEBNext poly(A) mRNA magnetic isolation module (New England Biolabs) and sequenced in paired end mode on a NextSeq 500 or a NovaSeq 6000 sequencer (Illumina) at the Genomics Facility, a core facility of the Interdisciplinary Centre for Clinical Research (IZKF) Aachen within the Faculty of Medicine at RWTH Aachen University. Each sample corresponded to one given animal, except for day 1 samples for which cells isolated from the small intestine of two 1-day-old animals were pooled.

### Proteomic analysis

Each sample corresponded to one given animal, except for day 1 samples for which cells isolated from the small intestine of three 1-day-old animals were pooled. For the sample preparation, isolated epithelial cells were lysed by addition of 150 µL lysis buffer (4% SDS, 100mM Tris-HCl pH 7.5, 100mM DTT) and heated for 5 min at 95 °C. Cell lysates were sonicated for 10 s, centrifuged at 16,000 x g in a tabletop centrifuge at RT and supernatant added onto 10 kDa cutoff filters (#OD010C33, PALL). Protein concentrations were measured at 280 nm using Nanodrop (Thermo Fisher Scientific). 250 µg of protein of each sample was alkylated and digested using filter-aided sample preparation [35]. Every sample was incubated with 0.45 µg trypsin at 37 °C overnight apart from the pooled samples of three 1-day old animals where 0.65 µg trypsin was used. Peptide concentration after elution was measured at 280 nm using NanoDrop and peptides cleaned with StageTip C18 columns prior to mass-spectrometry (MS) analysis [36].

Nano liquid chromatography (LC)-MS/MS was performed on a Q-Exactive HF mass-spectrometer (Thermo Fischer Scientific), connected with an EASY-nLC 1000 system (Thermo Fischer Scientific) through a nanoelectrospray ion source. Peptides were loaded on a reverse-phase column (150 mm^3^ 0.075 mm inner diameter, New Objective, New Objective, Woburn, MA) packed in-house with Reprosil-Pur C18-AQ 3 mm particles (Dr. Maisch, Ammerbuch, Germany). Peptides were separated with a 230-min gradient: from 3% to 25% B in 175 min, 25% to 45% B in 30 min, 45% to 100% B in 5 min, followed by 20 min wash with 100% of B (A: 0.1% formic acid, B: 0.1% formic acid/80% acetonitrile) using a flow rate of 250 nl/min. Q-Exactive HF was operated at 250 °C capillary temperature and 2.0 kV spray voltage. Full mass spectra were acquired in the Orbitrap mass analyzer over a mass range from m/z 350–1,600 with resolution of 60,000 (m/z 200) after accumulation of ions to a 3e6 target value based on predictive AGC from the previous full scan. The 12 most intense peaks with a charge state ≥2 were fragmented in the HCD collision cell with normalized collision energy of 27%, and tandem mass spectrum was acquired in the Orbitrap mass analyzer with resolution of 15,000 after accumulation of ions to a 1e5 target value. Dynamic exclusion was set to 30 s. The maximum allowed ion accumulation times were 20 ms for full MS scans and 50 ms for tandem mass spectrum.

### Sample preparation for single cell RNA-Seq

Here, single intestinal epithelial cells were isolated using a protocol adapted from Haber and colleagues [2]. Longitudinally cut-opened small intestines were placed in a 50 mL tube filled with PBS, shortly vortexed to remove the luminal content and further cut into 2- to 5-mm long pieces. The intestinal pieces were then transferred into a 50 mL tube prefilled with 30 mL room-tempered RPMI supplemented with 5% fetal calf serum (FCS) and 2 mM EDTA. Next, the tube was placed on a shaker set at 100 rpm at room temperature. After 15 min incubation, single epithelial cells were filtered through a 100 µm cell strainer, pelleted by centrifugation at 300  *g* at 4 °C. The pellet was resuspended in 1mL PBS containing 5% FCS and 0.01 mM EDTA and kept on ice. The remaining tissue pieces were transferred into a new 50 mL tubes containing 30 mL room-tempered RPMI supplemented with 5% FCS and 2mM EDTA and the cell isolation process was repeated three more times. The first fraction collected was discarded, while the epithelial cells contained in the three last fractions were pooled and stained using a rat anti-mouse PE-Cy7-labeled anti-EpCAM antibody (118,215, BioLegend), a rat anti-mouse FITC-labeled anti-CD45 antibody (103,108, BioLegend) and DAPI (Carl Roth). Single alive EpCAM^+^ CD45^−^ cells were sorted using the SH800 Cell Sorter (Sony). Isolation of barcoded RNA and subsequent cDNA library preparation were performed using the Chromium single cell 3′ reagent kits v2 and the Chromium Controller (10x Genomics), following manufacturer’s instructions. cDNA libraries were sequenced in paired end mode on a NextSeq 500 sequencer (Illumina) at the Genomics Facility, a core facility of the Interdisciplinary Centre for Clinical Research (IZKF) Aachen within the Faculty of Medicine at RWTH Aachen University. Each sample corresponded to three to six animals pooled. In addition, two datasets obtained from 2 distinct scRNA-Seq experiments were pooled for the analysis of the day 25 sample.

### Crypt and villi sample preparation for 3’RNA-Seq

Small intestines were rolled and snap-frozen in liquid nitrogen for 30 s to 1 min before being transferred to a −80 °C freezer until further use. 12µm-thick cryosections were then cut using a Leica Cryotome and mounted onto UV-treated Zeiss PEN membrane slides. Next, slides were transferred to successive bathes of 95% ethanol, 75% ethanol, 50% ethanol, 1% cresyl violet solution in DEPC-treated water, 50% ethanol, 75% ethanol, 95% ethanol, 100% ethanol (x2) for 30 s each. The slides were finally left for 5 min in 100% ethanol before being allowed to dry for 5 min at room temperature. Crypts (1 million µm²) and villi (2 million µm²) were then microdissected using a Zeiss PALM MicroBeam laser microdissection system (10x objective, cut energy 57 (1–100), cut focus 58–60 (1–100)) and catapulted into opaque UV-treated Zeiss adhesive caps. RNA was immediately isolated from the microdissected tissue pieces using the microRNeasy kit (Qiagen) following manufacturer’s instructions. Finally, cDNA libraries were prepared using the QuantSeq 3′ mRNA-Seq library prep kit FWD with the i5 6nt dual indexing add-on module (Lexogen) and sequenced in single end mode on a NextSeq 500 sequencer.

### Immunofluorescence staining

4µm-thick PFA-fixed paraffin embedded small intestinal tissue sections were stained with a rabbit anti-chromogranin A (23342-1-AP, Proteintech), rabbit anti-mucin 2 (GTX100664, Biozol), rabbit anti-lysozyme (A0099, Dako), rabbit anti-Ki67 (28074-1-AP, Proteintech), goat anti-IgA (1040-62, Southern Biotech) or mouse anti-E-cadherin (610182, BD biosciences) antibody and their appropriate fluorescence-labeled secondary antibodies (Dianova), counterstained with fluorescein‐conjugated wheat germ agglutinin (WGA, Vector) and mounted using DAPI-containing mounting medium (Vectorlabs). Chromogranin A^+^, mucin 2^+^, and lysozyme^+^ epithelial cells and IgA^+^ lamina propria cells were quantified per 624.70 µm × 501.22 µm fields and Ki67^+^ epithelial cells were quantified per crypt units. Twenty fields from 2 nonconsecutive tissue sections were analyzed per mouse and at least 38 crypt units were analyzed per mouse. 3 mice were analyzed per time point. Representative images were taken for each time point using an Axio Observer Z1 inverted phase contrast fluorescence microscope (Zeiss) equipped with the apotome.2 and colibri.2 (Zeiss) modules. In addition, 4µm-thick PFA-fixed paraffin-embedded small intestinal tissue sections were stained with hematoxylin and eosin to illustrate postnatal crypt development.

### Bioinformatic and statistical analysis

RNA-Seq raw sequence reads were trimmed for adapter/low-quality reads using cutadapt (version 4.4), aligned to the reference genome (mm10, GRCm38) using STAR (version 2.7.10a) and then a read count matrix was generated using the featureCounts function from the Rsubread package (version 2.14.2) [37]. Subsequent pre-analysis and differential expression analysis of the RNA-Seq data were carried out using the DESeq2 package (version 1.38.3) [38]. The raw read counts were normalized utilizing DESeq2’s VST method. Low count genes (overall expression below 60 counts in the dataset) were filtered out and zeroes were imputed. Global and crypt-villus transcriptomic datasets were batch-corrected using ComBat-seq [39]. To identify differentially expressed (DE) genes, a generalized linear model was employed to fit dispersion estimates, with the likelihood ratios test (LRT) used to establish statistical significance. Genes that showed an adjusted *p*-value (adjustment method Benjamini-Hochberg) of less than 0.05 and log_2_ fold change (FC) higher/lower than 1/-1 were identified as DE. Additionally, the ashr method was applied to shrink log_2_ FCs [40]. To allow overall comparability, the FCs and adjusted *p*-values in each analysis were capped at maximum and minimum thresholds corresponding to the 0.01 and 99.9 percent quantiles (negative and positive fold-changes) and the 0.01 percentage quantile for adjusted *p*-values in the volcano plots. Supplementary tables contain the raw, uncapped data.

Temporally DE genes were identified by comparing DE genes between any two consecutive timepoints (d05 *versus* d01; d10 *versus* d05; d25 *versus* d10). Hierarchical clustering was performed on gene expression value, with the ward.D2 method empirically chosen for gene clustering. Enrichment of temporal DE patterns for previously published BLIMP1- and MAFB-responsive genesets was assessed using Fisher’s exact tests [7,8]. For each temporal pattern and each TF-regulated geneset (up/down), a 2 × 2 contingency table was constructed based on overlap between the temporal DE genes and the TF-associated list, using all detected genes in the count matrix as the background. Tests were performed in R using a two-sided Fisher’s exact test, and *p*-values were adjusted using the Benjamini–Hochberg FDR method.

For each intestinal epithelial zone (proximal, medial, distal), the top 50 genes were selected based on adjusted *p*-value (*P*adj. < 0.05) and mean expression values (variance stabilized; VST) at each developmental time point (d01, d05, d10, d25) were calculated for these genes. Spearman correlation was computed between expression at each time point and d25, generating pairwise correlations for each zone.

MS raw files were processed with MaxQuant software version 1.5.7.4 [41], peak lists were identified by searching against the mouse UniProt protein database (downloaded 2018.07.11). Searches were performed using trypsin as an enzyme, maximum 2 missed cleavages, precursor tolerance of 20 ppm in the first search used for recalibration, followed by 7 ppm for the main search and 0.5 Da for fragment ions. Carbamidomethylation of cysteine was set as a fixed modification, methionine oxidation and protein N-terminal acetylation were set as variable modifications. The required false discovery rate (FDR) was set to 1% both for peptide and protein levels and the minimum required peptide length was set to seven amino acids. Label free quantification (LFQ) based on two peptides was used. The proteomic dataset was analyzed using the limma R package (version 3.54.2) [42]. Protein intensities were log-transformed, normalized, and fitted to a linear model, with an empirical Bayes model applied to stabilize variance estimates. Differentially abundant proteins were identified using the same thresholds as described before in the RNA-Seq data analysis. To identify the origin of the proteins that were present in the proteomic dataset but absent in the bulk RNA-Seq count matrix, we obtained the processed Seurat R-object files for mammary gland tissue published inside the *Tabula Muris* dataset [43]. The R-object already contained annotations and clustering of cell types. This annotation was consequently used to map the uncorrelated proteins to the specific cell types of the mammary gland tissue.

For the pre-processing of the scRNA-Seq data, de-multiplexing of fastq files and alignment to the mm10 mouse genome were performed using the Cellranger toolkit (version 3.1.0) by 10x Genomics and the version 2.5.1b of STAR included in Cellranger. Quality control, normalization, dimensionality reduction, unsupervised clustering and identification of DE genes were performed using the Seurat v4 R package [44]. During pre-processing, cells with less than 600 genes (nFeature_RNA) were filtered out. Clusters 2 and 11, as well as clusters 14–18 were then filtered out of the subsequent analysis, due to high mitochondrial counts and low cell count/cluster, respectively. Additionally, cells with fewer than 1,000 or more than 40,000 total RNA counts (nCount_RNA) were excluded to remove low-quality cells (e.g., empty droplets) and potential doublets. A subset of the 2,000 highest variable genes was identified using Seurat’s FindVariableFeatures function. Seurat’s default integration algorithm was used to combine datasets for the main analysis. Clusters were annotated based on canonical marker genes from the literature (Fig 5b) [2]. Smaller subclusters were merged to generate six clusters to accommodate to the six major small intestinal cell types (absorptive enterocytes, EECs, goblet and Paneth cells, stem cells, Transit-amplifying (TA) cells and tuft cells). TA cells were additionally distinguished from stem cells by using a threshold of above 50 percent marker gene expression between the canonical TA markers, which were also expressed in stem cells against the expression of stem cell markers. Cells with above 50 percent TA marker and below 50 percent stem marker gene expression were considered to be TA cells.

**Fig 2:**
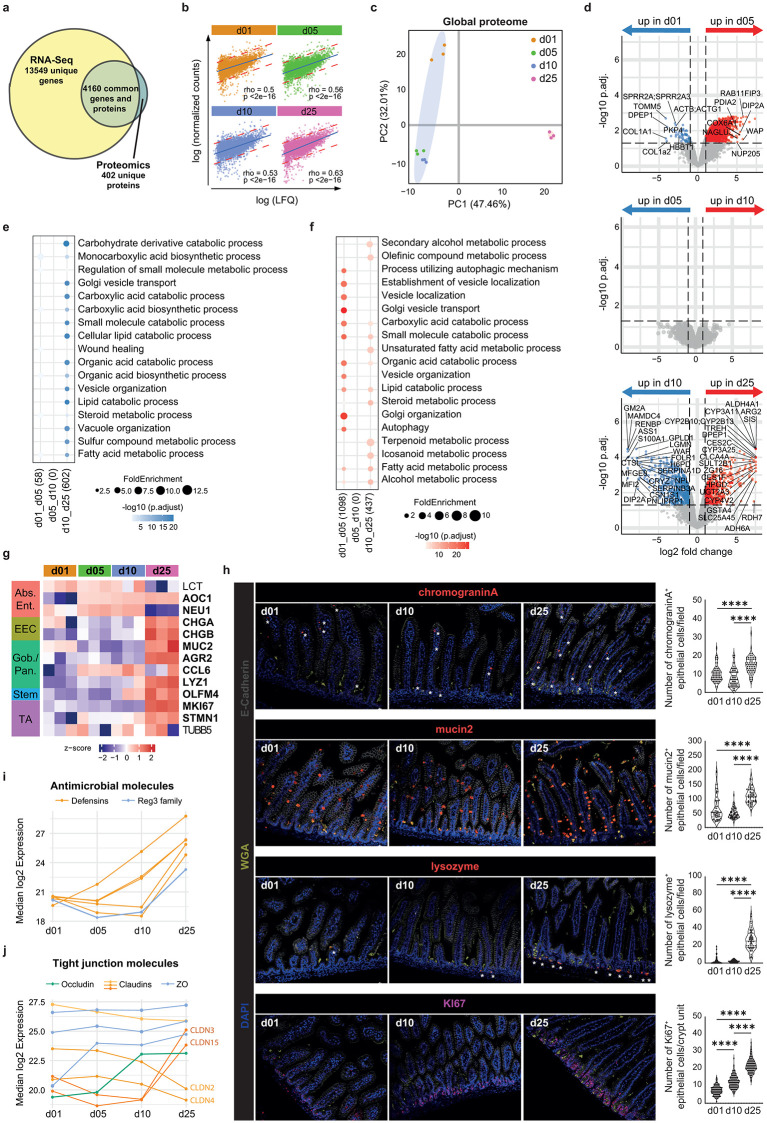
The global intestinal epithelial proteome during postnatal development. **(a)** Venn diagram representing the overlap between the RNA-Seq and proteomic datasets. **(b)** Correlation of the expression level of the 4,160 common genes and proteins detected by RNA-Seq and proteomics in 1-day-old (orange), 5-day-old (green), 10-day-old (blue) and 25-day-old (pink) animals. Spearman correlation values and *p*-values are shown for each age. **(c)** PCA plot representing the full small intestinal epithelial proteome of 1-day-old (*n* = 3), 5-day-old (*n* = 3), 10-day-old (*n* = 3) and 25-day-old (*n* = 3) mice. Ellipses were computed using the Khachiyan algorithm and show the pre-weaning (light blue) and post-weaning (light pink) samples. Color code as in Fig 2b. **(d)** Volcano plots showing the differential expression of proteins between 1- and 5-day-old (top), 5- and 10-day-old (middle), and 10- and 25-day-old animals (bottom). Blue and red dots indicate proteins statistically (*P*adj < 0.05) upregulated (|log_2_FC| > 1) in the younger and older animals, respectively. Gray dots indicate nonsignificantly and/or nondifferentially regulated proteins. **(e)** Dot plot showing the top 10 enriched GO terms (by adjusted *p*-value) for the DE genes downregulated with age. The number of DE genes associated with a GO term for each comparison is indicated in brackets. **(f)** Dot plot showing the top 10 enriched GO terms (by adjusted *p*-value) for the DE genes upregulated with age. The number of DE genes associated with a GO term for each comparison is indicated in brackets. **(g)** Heatmap depicting the expression level of known markers for absorptive enterocytes (Abs.Ent.), goblet/Paneth (Gob./Pan.) cells, enteroendocrine cells (EEC), stem cells, and transit amplifying (TA) cells. Bold names represent proteins that were identified as DE between at least 2 consecutive time points. **(h)** Immunofluorescence images of small intestinal tissue sections of 1-, 10- and 25-day-old mice stained for either chromogranin A, mucin 2, lysozyme (all red) or Ki67 (purple) (left) and corresponding image quantification (right). Counterstaining with DAPI (blue), WGA (green) and E-cadherin (gray). For the image quantification, 20 fields (624,70µm x 501,22µm) or at least 38 crypt units along the proximal-distal axis were analyzed per animal. 3 animals were analyzed for each age. Asterisks show positive cells. Kruskal-Wallis test with Dunn’s multiple comparisons post-test. **(i, j)** Temporal expression of known antimicrobials (i) and tight junction proteins (j). Underlying data for panels c, d, e, f, g, i and j can be found in S4 Data. Underlying data for panel b can be found in S5 Data. Underlying data for panel h can be found in S6 Data. Dataset available under the identifier PXD059639 at the ProteomeXchange Consortium.

**Fig 3:**
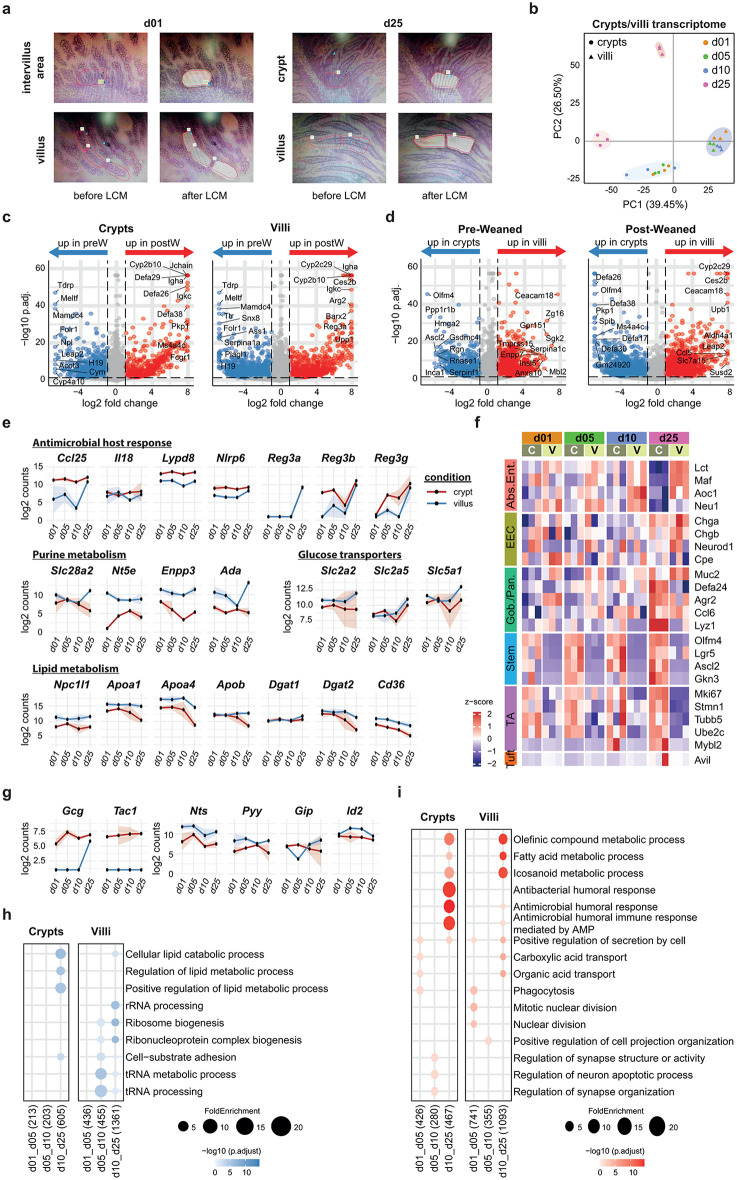
Spatial epithelial gene expression along the crypt-villus axis. **(a)** Pictures of small intestinal tissue sections, obtained from 1-day-old (left) and 25-day-old (right) animals and stained using cresyl violet. Pictures were taken before (left) and after (right) laser capture microdissection (LCM) of intervillus/crypt (top) and villus (bottom) area. **(b)** PCA plot representing the small intestinal epithelial transcriptome of either the crypt/intervillus area (circles) or villi (triangles) of 1-day-old (orange, *n* = 3), 5-day-old (green, *n* = 3), 10-day-old (blue, *n* = 3), and 25-day-old (pink, *n* = 3) mice. Ellipses were calculated using the Khachiyan algorithm and show the pre-weaning (crypts: light blue; villi: gray blue) and post-weaning (crypts: light pink; villi: gray pink) samples. **(c)** Volcano plots showing differential gene expression between pre-weaning (1-, 5- and 10-day-old) and post-weaning (25-day-old) crypt samples (left) and pre-weaning and post-weaning villi samples (right). Blue and red dots indicate genes statistically (*P*adj < 0.05) upregulated (|log_2_FC| > 1) in the younger and older animals, respectively. Gray dots indicate nonsignificantly and/or nondifferentially regulated genes. **(d)** Volcano plots showing differential gene expression between crypts and villi in pre-weaning (1-, 5- and 10-day-old) (left) and in post-weaning (25-day-old) (right) samples. Blue and red dots indicate genes statistically (*P*adj < 0.05) upregulated (|log_2_FC| > 1) in the crypt and villi, respectively. Gray dots indicate nonsignificantly and/or nondifferentially regulated genes. **(e)** Temporal expression pattern of selected genes encoding antimicrobial, metabolic, and transport molecules in the crypt *vs.* villus epithelium [3]. Median (lines) and median absolute deviation (MAD) (patches). **(f)** Heatmap depicting the expression level of known marker genes for absorptive enterocytes (Abs.Ent.), enteroendocrine cells (EEC), goblet/Paneth (Gob./Pan.) cells, stem cells, transit amplifying (TA) cells and tuft cells in 1-, 5-, 10-, and 25-day-old crypt/intervillus area (C) and villi (V) samples. **(g)** Temporal expression pattern of EEC-specific hormone genes in the crypt *vs.* villus epithelium. Median (lines) and median absolute deviation (MAD) (patches). **(h)** Dot plot showing the top 3 enriched GO terms (by adjusted *p*-value) for the DE genes downregulated with age in crypts (left) and in villi (right). The number of DE genes associated with a GO term for each comparison is indicated in brackets. **(i)** Dot plot showing the top 3 enriched GO terms (by adjusted *p*-value) for the DE genes upregulated with age in crypts (left) and in villi (right). The number of DE genes associated with a GO term for each comparison is indicated in brackets. Underlying data for panels b, c, d, e, f, g, h and i can be found in S9 Data. Dataset available under GSE283142.

**Fig 4:**
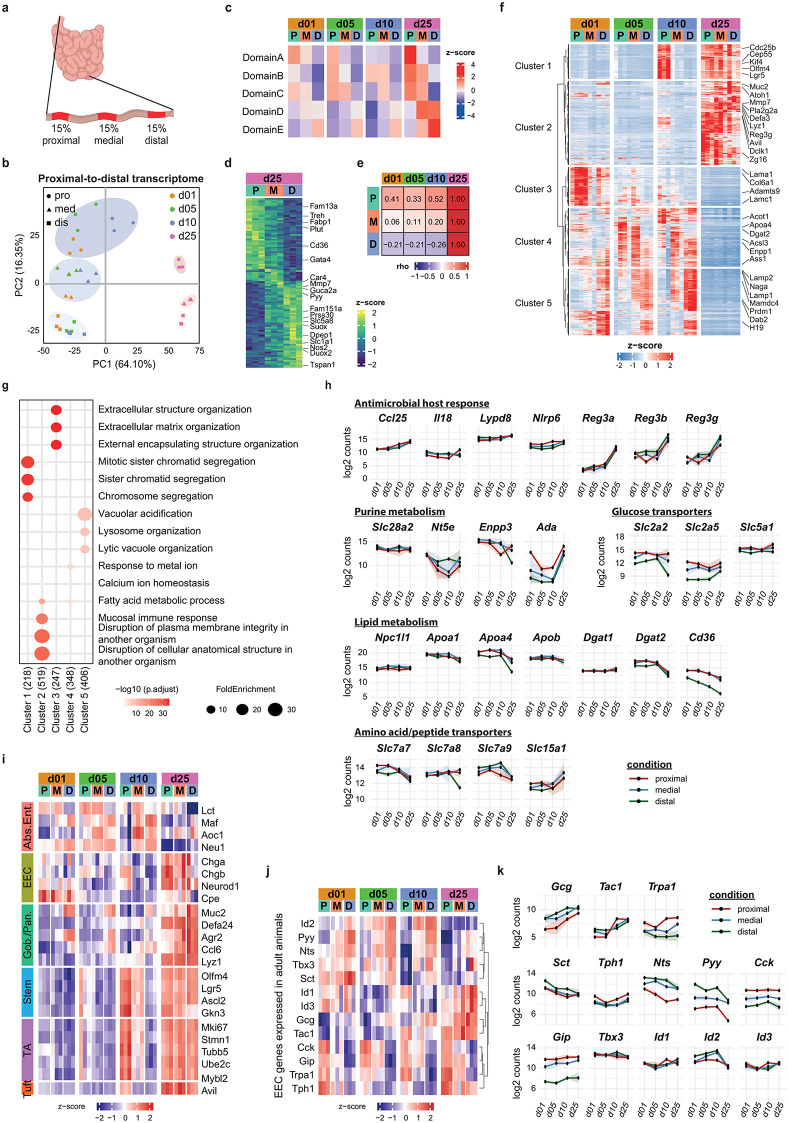
Spatial epithelial gene expression along the length of the small intestine. **(a)** Schematic representation of the small intestine showing the different samples collected. Created in BioRender. Dupont, A. (2026) https://BioRender.com/txlwv50. **(b)** PCA plot representing the epithelial transcriptome of either the proximal (circles), medial (triangles) or distal (squares) part of the small intestine of 1-day-old (orange, *n* = 3), 5-day-old (green, *n* = 3), 10-day-old (blue, *n* = 3) and 25-day-old (pink, *n* = 3) mice. Ellipses were estimated using the Khachiyan algorithm and show the distribution of the pre-weaning (proximal: light blue; medial: blue; distal: gray blue) and post-weaning (proximal: light pink; medial: pink; distal: gray pink) samples. **(c)** Heatmap depicting the combined expression level of the genes included in the 5 zonation quintiles along the proximal-to-distal length of the small intestine (as defined by Zwick and colleagues, domain A representing the most proximal end and domain E the most distal end [77]) in the proximal (P), medial (M) and distal (D) parts of the small intestine of 1-, 5-, 10- and 25-day-old animals. **(d)** Heatmap depicting the expression level of the top 50 genes (by adjusted *p*-value) differentially regulated in each part vs. the 2 other parts of the small intestine of 25-day-old animals. **(e)** Heatmap showing the correlation between the expression of the top 50 signature genes for the proximal, medial and distal parts of a 25-day-old animal (identified in Fig 4d) in 1-, 5- and 10- (and 25-)day-old animals. **(f)** Heatmap depicting the expression level of all genes differentially regulated between the proximal and medial part or the medial and distal part of 1-, 5-, 10- and/or 25-day-old animals. **(g)** Dot plot showing the top 3 enriched GO terms (by adjusted *p*-value) for the DE genes associated with each of the 5 clusters identified in Fig 4f. The number of DE genes associated with a GO term for each comparison is indicated in brackets. **(h)** Temporal expression pattern of selected genes encoding antimicrobial, metabolic, and transport molecules in the different segments of the small intestinal epithelium [3]. Median (lines) and median absolute deviation (MAD) (patches). **(i)** Heatmap depicting the expression level of known marker genes for absorptive enterocytes (Abs.Ent.), enteroendocrine cells (EEC), goblet/Paneth (Gob./Pan.) cells, stem cells, transit amplifying (TA) cells and tuft cells in the proximal, medial and distal parts of 1-, 5-, 10- and 25-day-old animals. **(j)** Heatmap depicting the expression level of known EEC genes [69]. **(k)** Temporal expression pattern of EEC-specific hormone genes in the different segments of the small intestinal epithelium. Median (lines) and median absolute deviation (MAD) (patches). Underlying data for panels b, f, h, i, j and k can be found in S10 Data. Underlying data for panels d and e can be found in S11 Data. Dataset available under GSE283143.

Three of the six identified clusters of cells (absorptive enterocytes, goblet/Paneth and EECs) were further divided into subclusters and reanalyzed to explore gene expression differences within each cell population. The Harmony algorithm was used to integrate the data of the goblet/Paneth and EEC subsets [45]. This reanalysis was followed by a new marker gene analysis to identify marker genes for each subcluster. To visualize the gradient of absorptive enterocytes from crypt to villus, we aggregated the expression of the top 50 DE genes (ranked by log_2_ FC) from pre-weaning crypts versus villi and post-weaning crypts versus villi obtained from the 3′ bulk RNA-seq dataset. These aggregated signatures were then overlaid onto the generated single-cell dataset to delineate crypt- and villus-associated regions. Likewise, the top 50 DE genes from pre-weaning proximal *versus* distal and post-weaning proximal *versus* distal segments were aggregated and overlaid onto the single-cell data to identify spatial zonation along the proximal–distal axis.

In addition, goblet and Paneth cells were identified using the expression of *Zg16* and *Lyz1*, respectively. The EEC subclusters were annotated using known marker genes from the literature [2]. For the absorptive enterocyte and goblet/Paneth clusters, DE marker genes (DE genes) were identified using a Wilcoxon signed-rank test with false discovery-rate correction (Seurat’s FindMarkers method). To reduce overall complexity, only consecutive timepoints (day 5 versus day 1, day 10 versus day 5, and day 25 versus day 10) were subsequently chosen for further analysis. Overall, the same thresholds as the ones described in the RNA-Seq analysis were chosen for the DE analysis.

For transcription factor (TF) analysis, we used the DoRothEA R package (version 1.18.0) to estimate TF activities from gene expression data [46,47]. DoRothEA employs a curated collection of TF-target interactions weighted by confidence levels (A to E). TF activities were computed using the VIPER algorithm, and results were scaled using z-scores for downstream analysis. Initially, TF activity was calculated per cluster for absorptive enterocytes, goblet/Paneth cells, EEC and stem cells; Tuft and TA cells were excluded due to low cell count) for each time point (day 1, 5, 10 and 25). To assess TF activity consistency across timepoints, we extracted significant TF activity results for the absorptive enterocytes, goblet/Paneth cells and EEC clusters and identified the top 15 most active TFs per time point based on z-score ranking. Presence-absence matrices were generated, with presence coded as 1 and absence as 0. Heatmaps were created using the ComplexHeatmap package (version 2.22.0), and hierarchical clustering was applied to visualize TF presence patterns across timepoints [48,49].

Pseudotime trajectories were inferred using slingshot (v. 2.14.0) [50]. Following the preprocessing and clustering workflow described above, PCA embedding was used as input for trajectory construction along the given cluster structure. The root cluster was defined biologically, based on prior annotations (clusters expressing bulk crypt genes, see above) to ensure correct trajectory orientation. Pseudotime trajectories were calculated for the subsets of enterocytes (pre-weaning and post-weaning) and goblet/Paneth cells (pre-weaning).

Pseudotime values were linearly scaled to a uniform range and divided into consecutive intervals of equal width, defining an ordered series of pseudotime bins inside the single cell object. Differential expression across bins was calculated using a log_2_ FC threshold of 1 and an adjusted *p*-value cutoff of 0.05. For each bin, the top 50 DE genes (ranked by adjusted *p*-value) were selected and average expression per bin was computed for this gene set, row-scaled (z-score), and filtered for low-variance features to capture pseudotime-dependent transcriptional changes. TF activity dynamics were evaluated by matching the previously calculated TF activity matrix to the corresponding cells, scaling each TF across cells, and calculating mean activity within each pseudotime bin. Top 100 TFs with the highest average activity across bins were retained to summarize pseudotime-dependent regulatory patterns.

DE gene-based gene ontology (GO) analysis was calculated in the same manner for all datasets. A comparative cluster analysis was done using the clusterProfiler package (version 4.12.6) [51]. Only the biological process (bp) ontology was used and only the top significant GO terms (*P*adj. < 0.05, adjustment method Benjamini–Hochberg) were reported.

Pathway activities were inferred using the PROGENy (version 3.19) and decoupleR (version 2.10) R packages for the RNA-Seq and proteomic datasets [47,52]. PROGENy computes scores for 14 signaling pathways based on gene expression data. Default settings were used, and pathway scores were scaled for comparisons. Boxplots were generated on these inferred pathway activities per time point, followed by an analysis of variance and a Tukey-HSD post-hoc-test.

To estimate Notch pathway activity across samples, we applied single-sample Gene Set Enrichment Analysis (ssGSEA) using the GSVA R package (v2.0.7) [53]. Mouse Notch pathway genes were retrieved from the REACTOME subset of the C2 collection in the mouse Molecular Signatures Database (MSigDB) [54]. Normalized RNA-seq counts and proteomic LFQ values were used as input, and ssGSEA scores were computed for each sample to obtain quantitative pathway activity. For visualization and comparison with PROGENy pathway scores, ssGSEA scores were scaled per pathway (z-score). Statistical differences across consecutive timepoints were assessed using one-way ANOVA followed by Tukey’s post-hoc test.

Statistical analysis of the immunofluorescence pictures was performed in GraphPad Prism 10 using the Kruskal-Wallis test with Dunn’s multiple comparisons post-test (3 groups) and the Mann-Whitney test (2 groups).

### Sex- and gender-based analyses

Since the focus of this study was on neonatal and infant samples collected prior to puberty of the animals, we did not analyze sex-specific characteristics. Both male and female neonatal and infant animals were included in the study; sex-specific genes such as *Xist*, *Jpx*, *Ftx*, *Tsx* and *Cnbp2* were removed from the analysis.

## Results

### Developmental rather than microbial factors drive global transcriptome changes during the early postnatal period

First, we performed bulk RNA-Seq on total small intestinal epithelial cells isolated from 1-, 5-, 10- and 25-day-old C57BL/6J SPF mice to identify the major transcriptional changes occurring during the early postnatal life. Principal component analysis (PCA) showed a pronounced difference between the pre-weaning (1-, 5- and 10-day-old) and post-weaning (25-day-old) samples along the first dimension (PC1, 72.7%), as well as a gradual shift with increasing age among the pre-weaning samples along the second dimension (PC2, 12.3%) (Fig 1b). Whereas expression of Paneth cell-associated antimicrobial genes (e.g., defensins (*Defa*), phospholipase A_2_ (*Pla2g2a*), and lysozyme (*Lyz1*) and the corresponding GO terms) increased steadily throughout the postnatal period, a marked increase in expression was noted at weaning for more broadly expressed factors of the antimicrobial program including the regenerating islet-derived proteins 3α, β and γ (*Reg3a, b* and *g*), interleukin-18 (*Il18*), the chemokine *Ccl25*, and the inflammasome component NOD-like receptor family pyrin domain containing 6 (*Nlrp6)* (Figs 1c, 1d, S1a, S1b, S1d and S1 Data). Similarly, the upregulation of some digestive enzymes (e.g., trehalase (*Treh*), sucrase isomaltase (*Sis*)) and of enzymes of the purine metabolism characteristically localized at the villus tip, such as the adenosine deaminase (*Ada*), was most pronounced at weaning (Figs 1c, 1d, S1a, S1b, S1d and S1 Data). Transmembrane transporters for iron (e.g., *Trf* and *Meltf*), folate (e.g., *Folr1*), vitamin A (e.g., *Ttr*), bile acids (e.g., *Abcb11*), glutathione peroxidases (e.g., *Gpx3*), as well as the multiligand endocytotic receptor *Lrp2*, were downregulated at weaning, whereas amino acid/peptide transporters such as the di- and tri-peptide transporter solute carrier family 15 member (*Slc15a1*, also known as peptide transporter, PEPT1) and the neutral amino acid transporter *Slc7a8* as well as the glucose transporters *Slc2a2*, *Slc2a5* and *Slc5a1* showed a marked increase in expression at weaning, likely corresponding to the change in dietary availability and/or metabolic demand at this critical time period (Figs 1c, 1d, S1a, S1b and S1 Data) [26,55–57]. Interestingly, whereas expression of the final enzyme in the triacylglycerol (TAG) synthesis diacylglycerol-O-acyltransferase 2 (*Dgat2*), the fatty acid translocase *Cd36,* and the apolipoprotein A-IV (*Apoa4*) decreased during the postnatal period, an increase in the expression of the nonessential TAG synthesis enzyme *Dgat1* and the important cholesterol transporter Niemann-Pick c1-like 1 (*Npc1l1*) was observed at weaning (Fig 1d). The expression of genes associated with tissue remodeling (GO terms “external encapsulating structure”, GO:0045229; “extracellular structure organization”, GO:0043062, and “extracellular matrix organization”, GO:0030198) decreased from birth on (S1c Fig, S1 Data). Gene expression of the endocytosis adapter disabled homolog 2 (*Dab2*), the endosomal protein endotubin (*Mamdc4*), the low density lipoprotein receptor-related protein 2 (*Lrp2*), and enzymes such as cathepsin L (*Ctsl*) was at its highest during the pre-weaning period, in line with the presence of fetal-type enterocytes with intraepithelial protein digestion in supranuclear vacuoles during early life (Figs 1c, S1a, S1b and S1 Data) [58]. Finally, genes downregulated between postnatal day 1 and day 5 included known fetal-specific genes (e.g., *H19*) (Figs 1c and S1a). Fewer genes were DE between day 5 and day 10, indicating a relatively stable situation of the epithelium between the two major transcriptional shifts directly after birth and at weaning (Fig 1c).

Pathway analysis using PROGENy revealed a transient increase in Jak/Stat signaling with a maximum at postnatal day 10, possibly related to the transient increase in mucosal cytokine expression during the so-called weaning reaction (Fig 1e) [15,16]. This analysis also identified an unexpected increase of the mitogen-activated protein kinase (MAPK), epidermal growth factor receptor (EGFR), and phosphoinositide 3-kinase (PI3K) pathways after weaning, whereas the NF-κB and TNF signaling pathways remained unaltered during the postnatal period (Figs 1e and S1f). The decrease in the transforming growth factor (TGF)β pathway after weaning might be due to the loss of breast milk-derived TGF-β or changes in the mesenchymal cell compartment providing BMP signals (Fig 1e) [59,60]. Interestingly, the epithelial WNT pathway decreased over time despite the delayed appearance of WNT ligand-producing Paneth cells, suggesting alternative dominant sources (Fig 1e) [61,62]. Finally, the fact that the hypoxia-mediated signaling pathway decreased after birth before increasing again at weaning was possibly related to the delayed luminal colonization by anaerobic bacteria (S1f Fig). No significant age-dependent alteration was found for the Notch pathway calculated based on the Reactome pathway database (Fig 1f) [63].

As transcriptional changes occurred for specific groups of genes at defined time points after birth (S1e Fig, S1 Data), a time pattern analysis of the gene expression between consecutive time points after birth (upregulated (U) and downregulated (D) DE genes or nonregulated genes (N) between 1- and 5-, 5- and 10- and 10- and 25-day-old) was performed (Fig 1g, top panel, S2 Data). It confirmed the previously reported dominant transcriptional shift occurring at weaning with NND and NNU being the two largest temporal patterns, comprising 1,568 and 1,415 genes, respectively. Genes within the NND and NNU, as well as the DNU, UND and UNU temporal patterns significantly correlated with a set of genes previously reported to be differentially regulated by BLIMP1 (encoded by *Prdm1*) and MAFB, two developmentally regulated TFs known to drive the intestinal epithelial neonatal-adult transition [6–9] (Fig 1g, bottom panel). Consistent with the strong influence of developmental factors, comparative analysis of the small intestinal epithelium of age-matched pre-weaned SPF and GF mice revealed relatively minor differences with few DE genes, whereas the comparison between 1- *versus* 5-, and 5- *versus* 10-day-old GF mice resembled the comparison of SPF mice at the corresponding age (S1g-S1i Fig, S3 Data). Similarly, no major differences were observed during the early postnatal phase between GF and SPF mice for selected genes with known important epithelial function (Figs S1j and 1d). This was unexpected and is in marked contrast to the situation in the adult individual, where the microbiota has been identified as influencing epithelial metabolic enzyme, glycosylation and antimicrobial effector gene expression [14,64]. Gene ontology (GO) analysis mainly assigned the NNU time pattern to metabolic and catabolic processes (e.g., “fatty acid metabolic process” GO:0006631, or “small molecule catabolic process” GO:0044282), suggesting increased active lipid and monosaccharide metabolism after weaning, and the NND time pattern to the loss of fetal-type enterocytes at weaning (e.g., “vacuolar acidification” GO:0007035, “lysosome organization” GO:0007040) (Fig 1h, S2 Data). In contrast, genes differentially regulated between 1 and 5 days after birth (DNN, 642 genes and UNN, 48 genes) were associated with Wnt signaling (GO:0016055), extracellular matrix organization (GO:0030198) and extracellular structure organization (GO: 0043062) (Fig 1h, S2 Data). Other temporal patterns identified smaller groups with less than 100 genes each that warrant future analysis (Fig 1g, top panel). Together, these results define the overall time course and relative contribution of epithelial transcriptional changes that accompany the establishment of mucosal host-microbial homeostasis.

Temporal gene expression differences may in part result from changes in the abundance of different epithelial cell types. Indeed, expression analysis of previously established marker genes for the different epithelial cell types revealed a significant upregulation mainly of genes expressed in goblet and Paneth cells, stem cells, TA cells, and tuft cells at weaning (Fig 1i) [2]. Consistently, expression of the majority of antimicrobial peptide and protein genes increased with age although at a somewhat different pace and with the notable exception of the cathelicidin CRAMP (*CAMP*) that decreased after weaning (Fig 1j, top panel, S1 Data) [31]. In contrast, expression of tight junction protein-encoding genes including claudins, zonula occludens proteins (ZO) and occludin remained relatively stable with few exceptions (Fig 1j, bottom panel, S1 Data). Expression of claudin (*Cldn*) 1 and 5 decreased steadily over time, whereas expression of claudin 8, 19 and less so 2 decreased, and claudin 15, 3, and less so 7 increased at weaning (Fig 1j, bottom panel, S1 Data).

### Proteome changes of the intestinal epithelium during the postnatal period and at weaning

The proteome analysis of total isolated small intestinal epithelium was based on 4,562 detected proteins, of which 4,160 were also detected at the transcriptional level by bulk RNA-Seq (Fig 2a, 2b). Mapping of the global small intestinal epithelial proteome of 1-, 5-, 10- and 25-day-old mice confirmed the strong difference between pre- and post-weaning samples (PC1, 47.5%; Fig 2c). Consistently, DE protein analysis showed increased expression of Paneth cell-associated antimicrobial effector molecules (e.g., DEFA, LYZ1), metabolic (e.g., SIS, TREH, dipeptidase 1 (DPEP1), maltase glucoamylase (MGAM) and xenometabolic enzymes (e.g., carboxylesterase CES1F and CES2C), transport molecules (e.g., solute carrier SLC5A45), mitochondrial proteins (e.g., arginase 2 (ARG2), beta-carotene oxygenase (BCO2), sulfitoxidase (SUOX)), and cell proliferation (e.g., MKI67) and tight junction (e.g., claudin CLDN15) proteins at weaning (Figs 2d, S2a, S2b and S4 Data). Proteins whose expression decreased at weaning were consistent with the previously observed transcriptional changes. Many were metabolic enzymes (e.g., argininosuccinate synthase 1 (ASS1), hexose-6-phosphate dehydrogenase/glucose-1-dehydrogenase (H6PD), N-acetylneuraminate pyruvate lyase (NPL) and GO terms related to catabolic/metabolic processes) or represented fetal-type enterocytes (e.g., MAMDC4, CTSL, alpha-N-acetylgalactosaminidase (NAGA) and GO terms “vesicle organization” and “vacuole organization”) (Figs 2d, 2e, S2a, S2b; S4 Data). PROGENy analysis and Reactome pathway analysis of the Notch pathway confirmed many of the changes of pathway activity observed using transcriptomics such as the JAK-STAT, MAPK, PI3K pathway (S2c, S2d Fig).

Whereas day 5 and 10 samples clustered closely together on the PCA (no DE proteins between them), they both clustered away from day 1 samples (PC2, 32.0%; Fig 2c). Interestingly, this was due to a high number of proteins being upregulated (1,098) rather than downregulated (58) between day 1 and day 5 (Fig 2e, 2f, S4 Data). Most of the early upregulated proteins were found within UNN and UND, two of the main time patterns identified by the proteomic analysis (S2e, S2f Fig, S7 Data). This sharply contrasted with the transcriptomic dataset which had fewer genes (126) differentially upregulated between day 1 and day 5 after birth (Fig 1c and 1g). A possible explanation for this finding is the presence of milk-derived proteins in the enterocytes of 5-day-old but not 1-day-old mice. Indeed, using a previously published single cell dataset of mammary gland tissue from the *Tabula Muris*, 135 of the 402 molecules detected at the protein but not mRNA level could be matched to genes transcribed in progenitor and mature luminal cells of breast tissue, suggesting their breast milk origin (S2h–S2j Fig, S8 Data) [43]. Consistently, typical milk proteins (e.g., whey acid protein (WAP), milk fat globule EGF and factor V/VIII domain containing (MFGE8), casein alpha S1 (CSN1S1)) were detected in day 10 but not day 25 samples (Fig 2d, S4 Data). Another explanation for the higher number of proteins upregulated between day 1 and day 5 might be due to nontranscriptionally regulated processes like autophagy (e.g., autophagy-related 3 (ATG3), GRIP and coiled-coil domain containing 2 (GCC2), dynactin subunit 1 (DCTN1), kinesin family member 3B (KIF3B), cullin 3 (CUL3), huntingtin (HTT), the GO terms “autophagy” (GO:0061919 and GO:0006914,) and “vesicle localization, organization and transport” (GO:0016050 and GO:0048193)) and lipid catabolism and metabolism (e.g., lipase E (LIPE), fatty acid transport protein 2 (SLC27A2), caseinolytic mitochondrial matrix peptidase chaperone subunit X (CLPX), galactosidase α (GLA), arylsulfatase A (ARSA), hexokinase 2 (HK2), aminoacylase 1 (ACY1) and aspartylglucosaminidase (AGA), and the GO terms “lipid catabolic process” (GO:0016042) “unsaturated fatty acid metabolic process” (GO:0033559) and “steroid metabolic process” (GO:0008202)) representing processes involved in the enteral intraepithelial trafficking and processing of dietary metabolites by fetal-type enterocytes (Fig 2f, and S4 Data) [8,26]. Alternatively, the increase in protein expression may reflect the increased mTOR- and eIF2a kinase-mediated autophagosome activity induced by metabolic starvation following the interruption of maternal transplacental nutrient supply at birth [65,66].

Many detected differences in protein expression were associated with the expression profile of specific epithelial cell types and may therefore be caused by changes in their abundance. Indeed, immunofluorescence staining and quantitative analysis of epithelial cells positive for chromogranin A (EECs), mucin 2 (goblet/Paneth cells), lysozyme (Paneth cells), and Ki67 (TA cells) confirmed a significant increase in the number of EECs, goblet/Paneth cells, and TA cells during the postnatal period and in particular between day 10 and 25 after birth (Fig 2h), consistent with the changes of cell type markers in the transcriptome (Fig 1i) and proteome (Fig 2g). In accordance with the increase of Paneth cells, antimicrobial proteins and peptides increased with age (Fig 2i, S4 Data). In contrast, expression of most tight junction proteins remained relatively stable except for an increase in claudin 3 and 15 and a moderate decrease of claudin 2 and 4 at weaning (Fig 2j, S4 Data).

### Age-dependent compartmentalization of gene expression along the crypt-villus axis

Analysis of the global intestinal epithelium only incompletely reflects the distinct anatomical compartmentalization along the crypt-villus axis and the functional specialization of the small intestinal epithelium (S3a Fig) [3,67,68]. To account for differences in the epithelial transcriptome, we next combined laser capture microdissection (LMD) with bulk RNA-Seq to individually analyze the transcriptome of the crypt (or intervillus area for pre-weaning animals) and villus epithelium (Fig 3a). Of note, although LMD was used to obtain epithelial cell material, the samples also contained transcripts from stromal (e.g., endothelin (*Edn3*)) and/or immune (e.g., immunoglobulin κ light chain (*Igkc*) and α heavy chain (*Igha*)) cells located in the vicinity of the intestinal epithelium (S9 Data).

PCA revealed a clear separation between the crypt/intervillus and villus epithelial transcriptomes at all ages (Fig 3b). This separation is consistent with a previous report of villus zonation of the epithelium in adult mice, although our crypt fraction associated with the lowest villus fraction of this report (S3b Fig) [3]. This epithelial zonation was observed at all ages, albeit to a lesser degree at early postnatal time points (S3b Fig). The comparison between the pre- and postweaning epithelium of specifically crypts or villi recapitulated many of the changes observed in total epithelial cells (Figs 3c, S1b). Irrespective of the age of the animal, genes differentially upregulated in the crypt/intervillus *versus* villus epithelium included stem cell markers (e.g., olfactomedin 4 (*Olfm4*)), molecules involved in DNA replication (e.g., PCNA clamp associated factor (*Pclaf*), topoisomerase (*Top2a*)) and antimicrobial host defense (e.g., *Defa*, deleted in malignant brain tumors 1 (*Dmbt1*)) genes (Figs 3d, S3c and S9 Data). Similarly, many of the genes associated with the antimicrobial host response program were more dominantly expressed in the crypt/intervillus epithelium at all ages, consistent with the protection of the regenerative stem cell niche against microbial challenge, whereas most transport molecules for dietary substrates such as glucose, lipids, or purine metabolites were more highly expressed by the villus epithelium, in closer contact to the luminal content (Fig 3e). No major zonal expression was found for amino acid/peptide transporters, except for *Slc15a1*, whose major upregulation previously observed at weaning happened dominantly in the villi (S3f Fig). Consistent with the presence of a crypt-villus zonation also in neonatal mice (S3b Fig), the crypt- and villus-specific analysis of cell type marker genes confirmed the localization of stem and TA cell markers to the intervillus/crypt area also in preweaning animals, although to a lesser degree (Fig 3f). Also, the previously reported localization of specific EEC subtypes to the crypt/intervillus *versus* villus epithelium in adult mice was confirmed in our study and could also be detected in neonatal mice, although less pronounced (Figs 3g, S3g, S3h) [69,70]. Crypt-dominated gene expression was found for glucagon (*Gcg*) and tachykinin (*Tac1*), whereas a more pronounced expression was found at the villus level for bile and pancreas-secretion inducing secretin (*Sct*) and cholecystokinin (*Cck*), neurotensin (*Nts*), neuropeptide YY (*Pyy*)*,* and transcriptional regulator inhibitor of DNA binding (*Id2*) throughout or at specific time points during postnatal development (Figs 3g, S3g, S3h). Interestingly, villus epithelial gene expression facilitating luminal sensing increased during the postnatal period for the satiety- and insulin-inducing but gastric acid-suppressing glucagon (Gcg) and gastric inhibitory peptide (Gip), whereas it decreased for the bile, gastric acid and pancreas secretion-inducing peptide hormones secretin (Sct) and cholecystokinin (Cck). This might reflect changes in the global energy metabolism after birth and the early start of hepatic bile acid synthesis and may support efficient absorption of breast milk nutrients [71,72].

PCA also confirmed the high similarity between the day 1, 5, and 10 samples and the strong difference between the pre- and post-weaning transcriptomes (Fig 3b). Compared to the pre-weaning intervillus epithelium, the post-weaning crypt epithelium was characterized by GO terms associated with antimicrobial responses and represented by increased Paneth cell-associated genes (e.g., *Pla2g2a*, *Defa, Reg3b, Reg3g*), consistent with the emergence of Paneth cells during the second week of life (Figs 3c, 3i, S3c, S3d and S9 Data) [23,31]. Also, the crypt/intervillus epithelium exhibited decreased expression of genes represented by the GO terms “regulation of lipid metabolic process” (GO:0019216) and “cellular lipid catabolic process” (GO:0016042) and a simultaneous increase of genes represented by the term “fatty acid metabolic process” (GO:0006631) at weaning, likely representing a shift from a “primed/regulatory” state to an “active execution” of lipid metabolism (Figs 3c, 3h, 3i, S3c, S3d and S9 Data) [73]*.*

In contrast, the post-weaning villus epithelium was characterized by an increased expression of genes represented by GO terms involved in nutrient digestion and transport (e.g., *Treh*, *Mgam*, nucleoside transporter *Slc28a1*, pyrimidine-degrading beta-ureidopropionase *Upb1*, cytochrome P450 *Cyp1a1* and *Cyp4b1*, monoamine oxidase A (*Maoa*)), and mucosal barrier molecules (e.g., *Reg3a*, *Muc3/Muc17)*, compared to the pre-weaning villus epithelium (Figs 3c, 3i, S3c, S3e and S9 Data) [74,75]. Translational activity (GO terms “ribosome biogenesis” (GO:0042254), “tRNA metabolic process” (GO:0006399), and “ribonucleoprotein complex biogenesis” (GO:0022613)) was downregulated after weaning, possibly reflecting the increased cellular turnover and reduced need for translational activity at the villus tip upon initiation of continuous crypt-villus cell migration at weaning (Fig 3h, S9 Data), whereas “fatty acid metabolic process” (GO:0006631) was upregulated representing the start of active lipid metabolism (Fig 3i, S9 Data).

Furthermore, both the neonatal intervillus and villus epithelium exhibited strong signs of intracellular vesicle trafficking and enzymatic activity, as evidenced by the increased gene expression of the small GTPase *Rab30*, sorting nexin 8 (*Snx8)*, cathepsins L and B (*Ctsl* and *Ctsb* (only in the villus for the latter)), and disabled adaptor protein 2 (*Dab2*)), in line with the macromolecule internalization and intraepithelial degradation of fetal-like enterocytes (Figs 3c, S3c–S3e and S9 Data) [8,26]. In contrast, increased expression of the IgA transport molecule polymeric immunoglobulin receptor (*pIgR*) in both post-weaning crypt and villus epithelium was accompanied by an increase of IgA-secreting plasma cells starting after weaning (Figs 3c, S3d–S3e, S3i and S9 Data) [76]. PROGENy and Reactome-based Notch pathway analysis assigned signaling pathways their anatomical localization (S3j–S3m Fig).

### Transcriptional differences along the proximal-to-distal length of the small intestine

In addition to the crypt-villus axis, the small intestinal epithelium is characterized by a strong anatomical and functional compartmentalization along its length [77]. To characterize segment-specific gene expression, we collected, at all ages, a fragment of the proximal, medial, and distal small intestinal epithelium, representing the duodenum, jejunum, and ileum, respectively (Fig 4a). The segments were well separated and anatomically defined on the PCA along the PC2 axis (16.4%), but these segmental differences were outweighed by the previously described pre- to post-weaning transition (PC1, 64.1%) (Figs 4b, S4a). The aggregated expression of segment-specific genes previously defined in adult mice (domain A to E, A being the most proximal) [77] recapitulated the proximal-to-distal segmentation of the day 25 samples but this correspondence was less pronounced in the preweaning samples (Fig 4c). We also defined segment-specific gene patterns in our own dataset based on the top 50 DE genes in the proximal, medial and distal segments of the 25-day-old intestine (Fig 4d). We then correlated the expression level in the proximal, medial and distal epithelium of 25-day-old mice with that of the corresponding segments in 1-, 5-, 10- (and 25-)day-old mice. The correlation coefficient indicated a decreasing correlation between the post-weaning proximal, medial, and distal and the preweaning proximal, medial, and distal expression profiles, suggesting a developmental delay of the medial and even more so distal segment of the intestinal epithelium during the postnatal period (Figs 4e, S4b and S11 Data) [1].

Hierarchical clustering of all DE genes identified 5 major clusters with differential segmental and/or temporal expression pattern (Fig 4f). Cluster 1, 3 and 5 showed the strongest segmental differences. Thus, cluster 1, characterized by GO terms “sister chromatid segregation” (GO:0000070) and “chromosome segregation” (GO:0007059), was represented by genes with a higher expression in all segments after weaning, but already in the proximal part at day 10 after birth, indicating that stem cell and TA cell proliferation started earlier in the proximal intestine (Figs 4f, 4g, S4c, S4d, S4f and S10 Data). Cluster 3 encompassed genes whose expression peaked at birth (1-day-old), more strongly and consistently in the proximal part, and that were characterized by GO terms such as “extracellular structure/extracellular matrix organization” (GO:0043062 and GO:0030198), indicating early tissue remodeling and maturation, a prerequisite for crypt formation (Figs 4f, 4g, S4c–S4e and S10 Data). Genes of cluster 5, represented by GO terms such as “lytic vacuole organization” (GO:0080171) or “lysosome organization” (GO:0007040), most likely representing fetal-type enterocytes, were more prominently and consistently expressed in the pre-weaning distal intestine, in line with the persistence of these enterocytes in the distal small intestine during the first 2 weeks of life (Figs 4f, 4g, S4c–S4e and S10 Data) [26]. Cluster 2, characterized by GO terms associated with “disruption of plasma integrity”/”disruption of cellular anatomical structure in another organism” (GO:0051673 and GO:0140975), “mucosal immune response” (GO:0002385), and “fatty acid metabolic process” (GO:0006631) most likely representing Paneth cell-derived antimicrobial molecules and the start of epithelial lipid metabolism, was comprised of genes uniformly upregulated in all segments post-weaning (Figs 4f, 4g, S4c, S4f). In contrast, genes from cluster 4 were upregulated in all segments during pre-weaning and were mostly associated with metabolic processes (Figs 4f, 4g, S4c–S4d, S4e and S10 Data). Similarly, except for *Slc7a8*, amino acid/peptide transporter and antimicrobial program gene expression exhibited little segmental differences, whereas the adenosin desaminase (*Ada*) showed increased expression in the proximal small intestine and the glucose transporters *Slc2a2* and *Slc2a5* and fatty acid translocase *Cd36* in the middle and proximal intestine at all ages, consistent with the known anatomical site of the maximal absorption of their respective substrate (Fig 4h).

Gene expression of marker genes of the crypt-based epithelial cell types, stem cells, TA cells and less so Paneth cells (*Lyz1*), increased at day 10 after birth in the proximal gut segments, consistent with the proximal-to-distal developmental wave (Fig 4i) [1]. In contrast, no differences were found between the different segments for the appearance of absorptive enterocytes and goblet cells (Fig 4i). Segmental differences of EEC hormone-encoding genes were noted, with a decreased expression of cholecystokinin (*Cck*), and the incretin gastric inhibitory peptide (*Gip*) transcripts and an increased expression of glucagon (*Gcg*), neurotensin (*Nts*), and the peptide YY (*Pyy*) transcript along the proximal-to-distal length of the intestine at all ages (Fig 4j, 4k).

### Age- and cell type-specific developmental trajectories revealed by single cell transcriptional analysis

**Fig 5:**
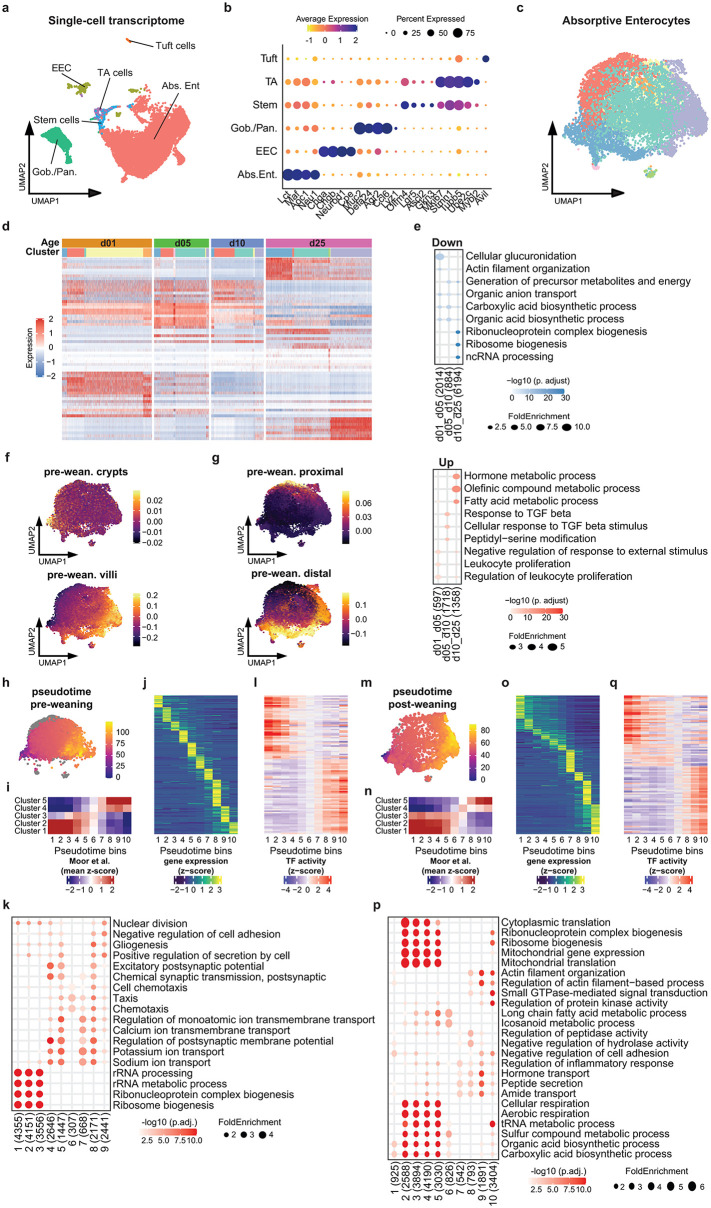
Single cell transcriptional analysis of intestinal epithelial cells. **(a)** UMAP plot displaying the different types of epithelial cells recovered from uninfected pre- and post-weaning murine small intestines: Absorptive enterocytes (red), Goblet/Paneth (green), stem (blue), transit amplifying (purple), enteroendocrine (ochre) and tuft (orange). **(b)** Dot plot displaying the marker genes for each cluster identified in Fig 5a. **(c)** UMAP plots showing the different subclusters recovered from the subclustering of the absorptive enterocyte cluster. **(d)** Heatmap representing the top 8 DE genes per absorptive enterocyte subcluster. Cluster color code as in Fig 5c. **(e)** Dot plot showing the top 3 enriched GO terms (by *P*adj) for the genes differentially downregulated (top) and upregulated (bottom) with age in absorptive enterocytes. The number of DE genes associated with a GO term for each comparison is indicated in brackets. **(f)** UMAP plots showing the expression level of genes previously found to be associated with pre-weaned crypts (top) and pre-weaned villi (bottom). **(g)** UMAP plots showing the expression level of genes previously found to be associated with pre-weaned proximal (top) and pre-weaned distal (bottom) samples. **(h–q)** Pseudotime analysis of the cells isolated from preweaned and postweaned samples. (h, m) UMAP plots showing the results of the pseudotime analysis for the pre-weaned (h) and post-weaned samples (m) using Slingshot. (i, n) Heatmap depicting the combined expression level of the genes included in the 5 zonation quintiles along the adult villus length (as defined by Moor and colleagues, cluster 1 representing the base of the villus and cluster 5 the tip of the villus [3]) along the pseudotime trajectory of preweaned (i) and postweaned (n) samples. (j, o) Heatmap depicting the expression of the top 50 marker genes for each bin of the preweaned (j) and postweaned (o) pseudotime trajectory. (k, p) Dot plot showing the top 3 enriched GO terms (by adjusted *p*-value) for the DE genes in each bin along the preweaned (k) and postweaned (p) pseudotime trajectory. The number of DE genes associated with a GO term for each bin is indicated in brackets. (l, q) Heatmap depicting the expression of the top 100 transcription factors (TF) identified by DoRothEA for each bin along the preweaned (l) and postweaned (q) pseudotime trajectory. Underlying data for panels b and e can be found in S12 Data. Underlying data for panel d can be found in S14 Data. Underlying data for panels f and g can be found in S15 Data. Underlying data for panel j, k, l, o, p ad q can be found in S16 Data. Dataset available under GSE284074.

Next, we performed single-cell RNA sequencing (scRNA-seq) and analyzed 15,582 high quality cells isolated from 1-, 5-, 10- and 25-day-old mice (d01-d25) (S5a–S5f Fig), as well as from *Salmonella*-infected animals (results presented in Fig 7). The single cell epithelial transcriptome of healthy animals captured all major intestinal epithelial cell types (i.e., absorptive enterocytes, goblet and Paneth cells, tuft cells, enteroendocrine cells, stem cells and TA cells), identified using established marker genes (Figs 5a, 5b, S5f) [2]. Of note, the isolation and flow cytometric sorting protocol of epithelial cells was optimized for the neonatal intestine, leading to an underrepresentation of crypt-based cell types such as stem and TA cells in the day 10 and day 25 sample (S5f Fig).

**Fig 6:**
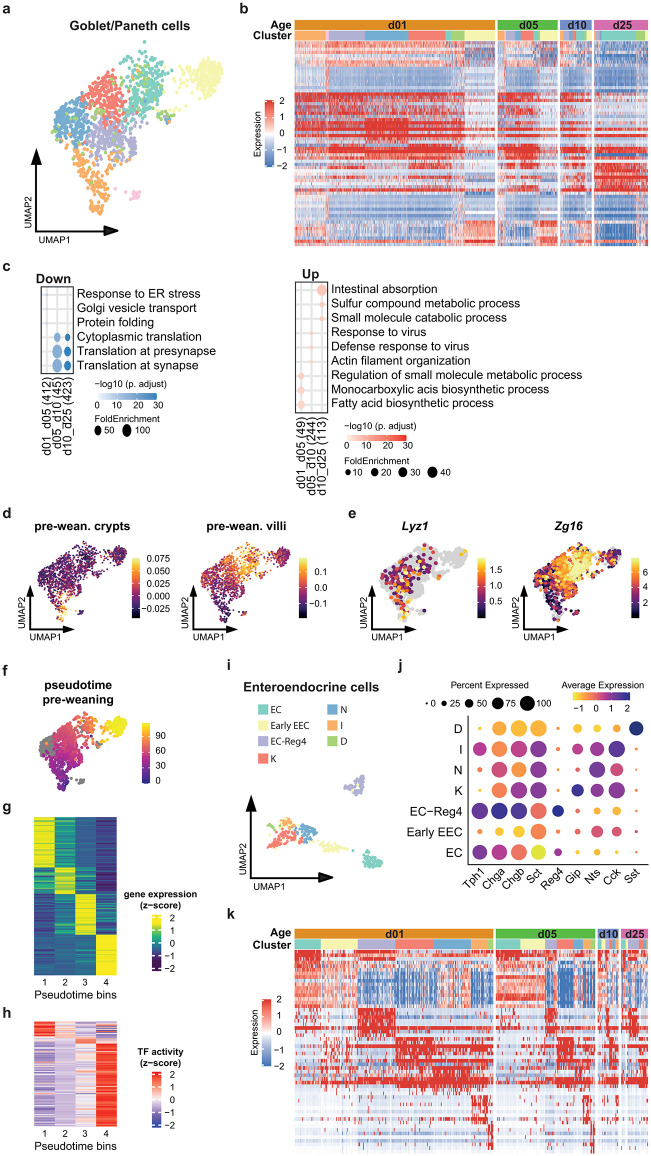
Single cell transcriptional analysis of secretory intestinal epithelial cells. **(a)** UMAP plots showing the different subclusters recovered from the subclustering of the goblet/Paneth cluster. **(b)** Heatmap representing the top 8 DE genes per goblet/Paneth subcluster. Cluster color code as in Fig 6a. **(c)** Dot plot showing the top 3 enriched GO terms (by *P*adj) for the genes differentially downregulated (left) and upregulated (right) with age in goblet/Paneth cells. The number of DE genes associated with a GO term for each comparison is indicated in brackets. **(d)** UMAP plots showing the expression level of genes previously found to be associated with pre-weaned crypts (left) and pre-weaned villi (right). **(e)** UMAP plots showing the expression level of known Paneth (*Lyz1*, left) and goblet cell (*Zg16*, right) markers. **(f–h)** Pseudotime analysis of the cells isolated from the preweaned samples. (f) UMAP plot showing the results of the pseudotime analysis for the pre-weaned samples using Slingshot. (g) Heatmap depicting the expression of the top 50 marker genes for each bin of the pseudotime trajectory. (h) Heatmap depicting the expression of the top 100 transcription factors (TF) identified by DoRothEA for each bin along the pseudotime trajectory. **(i)** UMAP plots showing the different subclusters recovered from the subclustering of the EEC cluster. **(j)** Dot plot displaying the marker genes for each cluster identified in Fig 5i. **(k)** Heatmap representing the top 8 DE genes per EEC subcluster. Subcluster color code as in Fig 5i. Underlying data for panels c and j can be found in S12 Data. Underlying data for panels b and k can be found in S14 Data. Underlying data for panel d can be found in S15 Data. Underlying data for panels g and h can be found in S17 Data. Dataset available under GSE284074.

**Fig 7:**
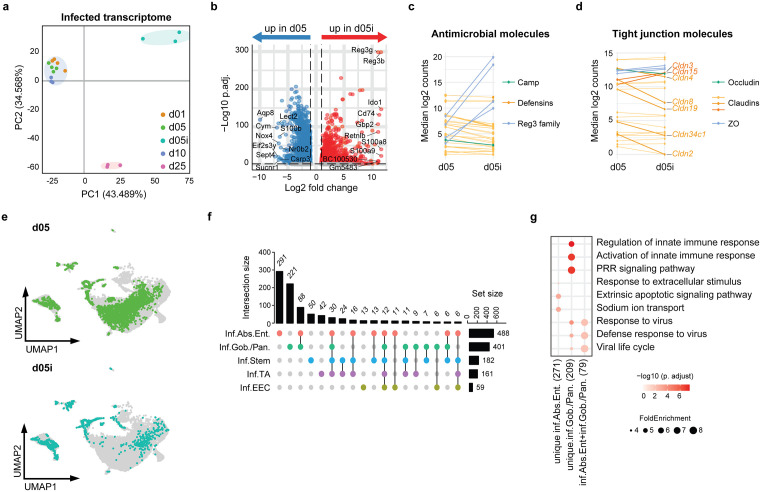
Transcriptional analysis of intestinal epithelial cells isolated from *Salmonella*-infected animals. **(a)** PCA plot representing the epithelial transcriptome of the small intestine of 1-day-old (orange, *n* = 4), 5-day-old (green, *n* = 4), 10-day-old (blue, *n* = 3) and 25-day-old (pink, *n* = 3) uninfected mice, as well as 5-day-old mice infected at birth with *S*. Typhimurium (turquoise, *n* = 3). Ellipses were estimated using the Khachiyan algorithm and show the distribution of the pre-weaning (light blue) and post-weaning (light pink) uninfected and 5-day-old infected (light turquoise) samples. **(b)** Volcano plots showing differential gene expression between 5-day-old *S*. Typhimurium-infected and uninfected samples. Blue and red dots indicate genes statistically (*P*adj < 0.05) upregulated (|log_2_FC| > 1) in the uninfected and infected animals, respectively. Gray dots indicate nonsignificantly and/or nondifferentially regulated genes. **(c, d)** Expression (counts) of genes encoding for known antimicrobial (c) and tight junction molecules (d) in infected and uninfected 5-day-old animals. **(e)** UMAP plots displaying the epithelial cells isolated from uninfected (top; green; same dataset as in S5e Fig) and *Salmonella*-infected (bottom; turquoise) 5-day-old newborn mice. **(f)** Upset plot showing the number of genes differently regulated by one or more epithelial cell type upon infection with *S*. Typhimurium. Absorptive enterocytes (Abs. Ent.; red), goblet/Paneth cells (GP; green), stem cells (blue), transit amplifying cells (TA; purple) and enteroendocrine cells (EEC; ochre). Only interactions with 10 and more DE genes are shown. **(g)** Dot plot showing the top 3 enriched GO terms (by adjusted *p*-value) for the interactions identified in Fig 6f that have more than 50 DE genes. The number of DE genes associated with a GO term for each comparison is indicated in brackets. Underlying data for panels c and d can be found in S1 Data. Underlying data for panels a, b, f and g can be found in S18 Data. Datasets available under GSE283495 and GSE284074.

We initially focused on the effect of age on epithelial cell type differentiation, since little is known about regulatory circuits that drive epithelial cell differentiation during the neonatal and early infant period [2,20]. We employed DoRothEA, a curated collection of transcription factor (TF) gene interactions, to identify cell type-specific TF activity [46]. This allowed the identification of cell type-dependent TF activity in the epithelium of 1-, 5-, 10-, and 25-day-old mice (S5g Fig and S13 Data) as well as the age-dependent TF activity in absorptive enterocytes, goblet/Paneth cells and EECs (S5h Fig and S13 Data). The majority of cell type-specific TF acted in an age-independent fashion (S5i Fig). A subsequent assembly of the TF with the strongest activity in each age group for absorptive enterocytes, goblet/Paneth cells, and EECs revealed mainly three patterns (S5h Fig): Firstly, the large group of TF with age-independent activity such as for example Hnf1a, Hnf4a, and Cebpe for absorptive enterocytes, Spdef, Tcf4, and Sox9 for goblet/Paneth cells, or Ets2 and Mafb for EECs. Secondly, TF with post-weaning activity such as Egr3 and Sp3 for absorptive enterocytes and E2f4 for EECs [78]. And finally, TF with strong pre-weaning activity such as Gata4, Lef1, Stat2, Usf2, and Cebpa that have been identified in the intestinal epithelium, but not associated with the neonatal period [12,79–81]. However, given that these TF were identified in cell type clusters of largely differentiated cells, it remains unclear whether these TF drive cell type-specific functions or lineage commitment.

Next, we analyzed the absorptive enterocyte cluster in more detail (Figs 5c and S5j). Subclustering and illustration of the top 8 DE genes per subcluster in a heatmap highlighted the age-specific appearance of individual cell subtypes (Fig 5d, S14 Data). GO term analysis of the age-dependent transcriptional profile of absorptive enterocytes confirmed the previously observed developmental changes of metabolic processes illustrated by the upregulation of genes represented by the GO term “fatty acid metabolic process” (GO:0006631), suggesting increased active lipid metabolism and identified a downregulation of translational activity with weaning (Fig 5e, S12 Data). In contrast to the identification of metabolic processes, the increase in TGF-β activity in absorptive enterocytes between day 5 and 10 after birth (e.g., GO terms “response to TGF beta”, GO:0071559 or “cellular response to TGF beta stimulus”, GO: 0071560) was not detected in the pathway analysis based on the global transcriptomic and proteomic profiles during this time window, highlighting the importance of cell type-specific analyses (Figs 5e; 1d; S2c, and S12 Data). Projection of gene sets found to represent preweaning or postweaning crypts and villi (Fig 3) as well as the preweaning or postweaning proximal and distal epithelium (Fig 4) revealed the anatomical localization of the cells within the absorptive enterocyte cluster (Figs 5f, 5g, S5k, S5l). Pseudotime analysis of preweaning absorptive enterocytes identified a developmental trajectory in neonatal mice that recapitulated the order of previously identified marker genes of the crypt-villus axis (Fig 5h, 5i). Similar trajectories were also found within the absorptive enterocyte cluster of 1-, 5-, and 10-day-old mice (S5m Fig). A heatmap of DE genes along the trajectory illustrated the developmental path of these cells (Fig 5j, S16 Data) and the GO term and TF analysis identified strong ribosomal activity in the early trajectory (crypt region), and cell transport in the late trajectory (villus region) consistent with previous findings (Fig 5k, 5l, and S16 Data). Similarly, a developmental trajectory was also identified in the postweaning absorptive enterocyte cluster (Fig 5m) and analyzed for its correlation with established crypt-villus zonation genes (Fig 5n), DE genes (Fig 5o, S16 Data), representative GO terms (Fig 5p, S16 Data) and TFs (Fig 5q, S16 Data).

Next, we analyzed the goblet/Paneth cell cluster in more detail (Fig 6a). Subclustering and illustration of the top 8 DE genes per subcluster in a heatmap highlighted the appearance of individual goblet/Paneth cell subtypes during the postnatal period (Figs 6b, S6a and S14 Data). GO term analysis of DE genes revealed reduced translational activity (GO:0002181) and increased intestinal absorption (GO:0050892) with increasing age, likely reflecting the bioenergetic demand rather than genuine absorptive function (Fig 6c, S12 Data). Also here, cells of the goblet/Paneth cell cluster localized along the preweaning and postweaning crypt-villus axis (Figs 6d and S6b) and Paneth (*Lyz1*) or goblet cell (*Zg16*) marker gene expression could be localized (Fig 6e). The low number of cells precluded pseudotime analysis of the postweaning sample, but pseudotime analysis of the pre-weaning samples revealed a developmental trajectory towards Zg16 positive cells and identified DE genes and TFs, characterizing the cellular differentiation along the trajectory (Fig 6f–6h, S17 Data).

In contrast, the seven identified enteroendocrine (EEC) subclusters could be assigned to seven known EEC subtypes based on previously reported marker genes and were detected at all ages with similar abundancy (Figs 6i–6k and S6c, S6d) [2]. A heatmap illustrating the top 8 DE genes per EEC subtype showed the typical “staircase” pattern for 1–5-, 10-, and 25-day-old mice, confirming the presence of the different EEC subtypes similarly in neonatal and adult animals consistent with previous findings (Fig 6k, S14 Data).

### The global and cell type-specific response to enteric infection

To investigate epithelial gene expression under challenged conditions, we also analyzed intestinal epithelial cells collected at day 4 post-infection from mice infected orally at day 1 after birth with 100–300 CFU *S.* Typhimurium (i.e., age-matched to the day 5 time point) [82]. Mapping of the small intestinal epithelial bulk transcriptome of 5-day-old *S.* Typhimurium-infected mice on that of 1-, 5-, 10- and 25-day-old healthy mice illustrated the major infection-induced transcriptional changes (PC1, 43.5%) consistent with our previous report (Fig 7a) [32]. DE gene analysis of day 5 uninfected and day 5 infected samples revealed upregulation of typical epithelial antimicrobial host response genes such as c-type lectins (e.g., *Reg3g*, *Reg3b*, *Reg3a*), alarmins (e.g., *S100a8*/*S100a9)*, inducible NO synthase 2 (*Nos2*), NAPDH oxidase 1 (*Nox1*), serum amyloid A1 (*Saa1*), and cytokines (e.g., *Tnf* and C-C motif chemokine ligand 8 (*Ccl8*)), but also a number of catabolic enzymes (e.g., tryptophan catalytic enzyme indoleamine 2,3-dioxygenase 1 (*Ido1*), uridine degrading enzyme uridine phosphorylase 1 (*Upp1*)) (Fig 7b and 7c, S1 and S17 Data). In contrast, expression of α-defensins remained unaltered consistent with their mainly posttranscriptional regulation (Fig 7c, S1 Data) [83]. Similarly, expression of the majority of tight junction proteins remained unaffected, although some claudins such as claudin 2, 4, 8, 19 and 34c1 exhibited an infection-induced downregulation, and claudin 3 and 15 were upregulated (Fig 7d, S1 Data). Differences between the models, e.g., immortalized cell lines *versus* primary epithelial cells within intact tissue, may explain the different outcome in previous reports [84,85]. Uniform Manifold Approximation and Projection (UMAP)s representing the scRNA-Seq of the uninfected (d05) and infected datasets (d05i) illustrated a marked decrease in the abundance of absorptive enterocytes in 5-day-old infected animals (Fig 7e). Given the previously shown largely preserved tissue architecture in infected animals, with only low numbers of cleaved caspase 3 positive enterocytes, this suggests that the inflammatory environment in vivo aggravated the cell stress during cell isolation and flow cytometric sorting leading to exclusion from the analysis due to low sequence data quality (Figs 7e, S5a–S5e) [82,86]. Interestingly, a reduced cell abundance was not observed for stem, Paneth/goblet, tuft or enteroendocrine cells, indicating a higher resilience of these cells towards the proapoptotic inflammatory environment in vivo. A subsequent cell type-specific analysis of the response to infection revealed sets of genes altered by infection in multiple cell types, but as well sets of altered genes unique to specific cell types (Figs 7f, S7a–S7e and S18 Data). GO term analysis of these uniquely induced genes suggested that absorptive enterocytes were affected by ion transport changes (“sodium ion transport”, GO:0006814) and the induction of extrinsic apoptosis (“extrinsic apoptotic signaling pathway”, GO:0097191) consistent with the increased loss of absorptive enterocytes in the infected tissue (Fig 7g, S18 Data). Although Reg3β and Reg3γ expression was ubiquitously observed (S7a–S7e Fig), many antimicrobial host response genes were uniquely upregulated in goblet/Paneth cells (e.g., GO term “regulation of innate immune response”, GO:0045088; “activation of innate immune response”, GO:0002218; and “PRR signaling pathway”, GO:0002221) (Fig 7g, S18 Data).

## Discussion

Our results illustrate the complexity and striking heterogeneity of the different neonatal small intestinal epithelial cell types, demonstrate intriguing alterations in epithelial gene and protein expression early after birth and identify functional changes that may contribute to the postnatal transition. Unexpectedly, global transcriptional differences between the proximal and distal intestine as well as between the crypt and villus epithelium were as pronounced in the neonate as compared to the adult host despite the lack of an organ site-specific microbiota composition, the lack of defined small intestinal crypts and a reduced mucus production, making the neonatal mucosal architecture less compartmentalized [24,72,82]. Also, whereas the enteric microbiota has a significant impact on the epithelial expression of host defense, glycosylation and metabolic genes in adult animals, our epithelial transcriptome suggested little differences between SPF and GF mice in pre-weaning animals [14]. Although our global analysis does not exclude an influence of early life microbial signals on the acquisition of specific epithelial cell functions, for example as previously indicated for Paneth cells [87], it suggests that the low diversity and large interindividual variation of the early microbiota composition may fail to provide reliable innate immune signals, thus favoring developmental programming to steer the neonate intestinal epithelium [72,88].

Although the establishment of intestinal epithelial stem cell organoids representing all epithelial cell lineages has been a major breakthrough in intestinal epithelial cell research [89], organoids grown in vitro still lack the influence of the enteric microbiota, dietary, nervous and immune cell signals, as well as hormones and tissue ontogeny, supporting the need to analyze ex vivo isolated primary cells [19,90]. Nonetheless, the isolation and analysis of primary small intestinal epithelial cells remain technically challenging. Firstly, detachment from the basal membrane, a prerequisite for the isolation and analysis of epithelial cells, reduces their fitness and together with the proapoptotic signals during infection-induced inflammation may reduce their viability [91]. Secondly, the anatomical organization of the intestinal epithelium makes it difficult to isolate representative fractions of viable cells simultaneously from villi and crypts at different ages using the same protocol [92]. As our analysis focused on the early postnatal period, we prioritized villus cell over adult crypt cell coverage, which explains the underrepresentation of crypt-based stem, TA, and Paneth cells in our adult sample. However, the crypt epithelium of adult mice has been previously characterized [2,3].

All intestinal epithelial cell types arise from Lgr5^+^ pluripotent stem cells [10,11,93]. Notch-mediated lateral inhibition and a complex network of epithelial and mesenchymal signals and TFs drive differentiation into the specific cell type lineages [1,24,94]. Many largely uncharacterized TFs were significantly associated with specific epithelial cell types in the neonate and await further analysis. Interestingly, fetal TFs have been shown to reemerge and influence cell differentiation in the inflamed infant intestine [95]. In addition, migration along the crypt-villus axis exposes cells to spatial gradients of proliferation and differentiation-regulating factors [24], and a proximal-to-distal gradient, while under marked transcriptional control, may also be influenced by differences in the concentration of microbial or dietary stimuli [12,96].

Major differences in the intestinal epithelial gene and protein expression were observed at weaning, largely explained by the transcriptional influence of two of the major transcriptional regulators of the pre- to post-weaning transition, BLIMP1 (*Prdm1*) and MAFB [6–9]. They included changes in the expression of digestive enzymes reflecting the transition of breast milk to solid food [6,7]. Cessation of breast milk feeding and thereby cessation of contact with the breast milk constituents EGF and TGF-β reduced TGF-β and EGF receptor signaling in epithelial cells after weaning [73]. Also, the emergence of crypts and crypt-based Paneth cells was identified by the upregulation of typical Paneth cell genes such as α-defensins and phospholipase A_2_ [23,31,93]. The global analysis of antimicrobial peptides and proteins suggest that the largely post-transcriptional regulation of Paneth cell α-defensins in the adult host, may be substituted by a strong and global upregulation of the c-type lectins such as Reg3γ in the absence of mature Paneth cells in the neonatal host upon infectious challenge [31,32,82,83]. The transcriptional changes of both digestive enzymes and antimicrobial molecules were found throughout the intestinal tract. In contrast, epithelial cell proliferation, as a sign of the establishing crypt niche, started earlier in the proximal part of the intestine consistent with the proximal-to-distal developmental wave [1]. The previously described “weaning reaction” in the mucosal tissue that reflects immune maturation and is accompanied by elevated expression of pro-inflammatory cytokines may be responsible for enhanced epithelial signaling via the Jak/Stat, MAPK and PI3K pathway [15].

Important transcriptomic and proteomic changes were also detected earlier, between day 1 and 5 after birth. In contrast to the transcriptome analysis, the proteome analysis revealed mostly proteins upregulated between day 1 and 5 after birth. In part, this conundrum could be explained by the presence of proteins produced by mature luminal cells in the breast gland [43]. Milk proteins in isolated intestinal epithelial cells most likely reflects the presence of fetal-type enterocytes that internalize luminal proteins by endocytosis followed by intraepithelial degradation by cathepsins [26]. Fetal-type enterocytes were restricted to the pre-weaning period and their gene signature was most abundant in the distal part of the small intestine, consistent with the literature [26]. In addition, the detection of proteins in the absence of their corresponding transcripts may be due to increased autophagocytotic activity in the neonatal host, providing peptides amenable to mass spectrometric detection. Autophagy has been shown to be critical to overcome the energy shortage of the neonatal host imposed upon birth and until establishment of enteral feeding [65,66]. Both aspects illustrate the unique situation of the neonatal host and identify important strategies to adapt to postnatal life.

Together, we used state-of-the-art methods and models to characterize the global, organ- and site-specific as well as single-cell transcriptional and proteomic profile of the gut epithelium at different time points after birth. Our results identify and characterize key temporal, organ site, and cell type-specific gene expression profiles, determine age-dependent signaling pathway activity, and define age- and cell-type-specific TF activity, illustrating the strong developmental influence and only marginal effect of the enteric microbiota during the early postnatal period. We characterize the age-dependent developmental trajectories of individual cell types, highlighting the compartmentalized and discontinuous transcriptional profiles of specific cell type and describe the global and cell-type-specific response to neonatal enteric infection. The intestinal epithelium directly or indirectly interacts with stromal, nervous and immune cells in the intestinal tissue and major changes in the cell type abundance, cell differentiation, activation and cellular effector functions have been described during the postnatal period for epithelial cells, but also for T and B lymphocytes [15,97], dendritic cells [76], lymphatic endothelial cells [98], lymph node stromal cells [99] and nervous cells [100–102]. The present study solely focuses on the postnatal development of the intestinal epithelium and provides the basis for future investigations, but, ultimately, an integrated view on the postnatal development of all individual cell types and their interaction during postnatal maturation will be needed to fully understand the complexity of the postnatal transition and help to identify strategies to reduce childhood mortality and promote mucosal homeostasis and long-term health.

## Supporting information

S1 Fig (a) Biplot displaying the top 50 driver genes for the 1st and 2nd dimension of the PCA plot shown in Fig 1b. Black/blue names represent genes that were identified as DE/non-DE between pre- and post-weaning samples. 1-day-old (orange dots), 5-day-old (green dots), 10-day-old (blue dots) and 25-day-old (pink dots). **(b)** Volcano plots showing differential gene expression between pre-weaning (1-, 5- and 10-day-old) and post-weaning (25-day-old) samples. Blue and red dots indicate genes statistically (*P*adj < 0.05) upregulated (|log_2_FC| > 1) in the younger and older animals, respectively. Gray dots indicate nonsignificantly and/or nondifferentially regulated genes. **(c)** Dot plot showing the top 3 enriched GO terms (by adjusted *p*-value) for the DE genes downregulated with age (blue DE genes from Fig 1c). The number of DE genes associated with a GO term for each comparison is indicated in brackets. **(d)** Dot plot showing the top 3 enriched GO terms (by adjusted *p*-value) for the DE genes upregulated with age (red DE genes from Fig 1c). The number of DE genes associated with a GO term for each comparison is indicated in brackets. **(e)** Heatmap depicting the expression level of all genes differentially regulated between either 1- and 5-day-old, 5- and 10-day-old and/or 10 and 25-day-old animals. **(f)** Box plots showing PROGENy pathway analysis at the different ages. Color code as in S1a Fig. ANOVA with Tukey-HSD post-hoc-test. **(g)** PCA plot representing the full small intestinal epithelial transcriptome of 1-day-old (orange, *n* = 4/4 SPF/GF), 5-day-old (green, *n* = 4/4) and 10-day-old (blue, *n* = 3/4) specific pathogen free (SPF, circles) and germ-free (GF, triangles) mice. Ellipses were estimated using the Khachiyan algorithm and show the distribution of samples for each age group. **(h)** Volcano plots showing differential gene expression between 1- and 5-day-old (left) and between 5- and 10-day-old (right) GF samples. Blue and red dots indicate genes statistically (*P*adj < 0.05) upregulated (|log_2_FC| > 1) in the younger and older animals, respectively. Gray dots indicate nonsignificantly and/or nondifferentially regulated genes. **(i)** Volcano plots showing differential gene expression between GF and SPF mice at 1 (left), 5 (middle) and 10 (right) days of age. Blue and red dots indicate genes statistically (*P*adj < 0.05) upregulated (|log_2_FC| > 1) in GF and SPF animals, respectively. Gray dots indicate nonsignificantly and/or nondifferentially regulated genes. **(j)** Temporal expression pattern of selected genes encoding antimicrobial, metabolic, and transport molecules in 1-, 5-, and 10-day old GF mice [3]. Median (lines) and median absolute deviation (MAD) (patches). Underlying data for panels a, b, c, d, e, f and j can be found in S1 Data. Underlying data for panels g, h and i can be found in S3 Data. Dataset available under GSE283495.
(EPS)

S2 Fig (a) Biplot displaying the top 50 driver proteins for the 1st and 2nd dimension of the PCA plot shown in Fig 2c. Black/blue names represent proteins that were identified as DE/non-DE between pre- and post-weaning samples. 1-day-old (orange dots), 5-day-old (green dots), 10-day-old (blue dots) and 25-day-old (pink dots). **(b)** Volcano plots showing differential protein expression between pre-weaning (1-, 5- and 10-day-old) and post-weaning (25-day-old) samples. Blue and red dots indicate proteins statistically (*P*adj < 0.05) upregulated (|log_2_FC| > 1) in the younger and older animals, respectively. Gray dots indicate nonsignificantly and/or nondifferentially regulated proteins. **(c, d)** Box plots showing PROGENy pathway analysis (c) and NOTCH pathway analysis (d) at the different ages. Color code as in S2a Fig. ANOVA with Tukey-HSD post-hoc-test. **(e)** Heatmap depicting the expression level of all proteins differentially regulated between either 1- and 5-day-old, 5- and 10-day-old and/or 10 and 25-day-old animals. **(f)** Six most abundant temporal postnatal protein expression patterns as identified by temporal DE analysis. The number of DE proteins for each pattern is indicated in brackets. **(g)** Dot plot showing the top 5 enriched GO terms (by *P*adj value) for the DE genes identified as belonging to the NND, NNU, UND and UNN expression patterns. The number of DE proteins associated with a GO term for each pattern is indicated in brackets. **(h)** UMAP plot displaying the different types of cells in breast tissue identified by the previously published *Tabula Muris* and overlapped with the expression level of the intestinal epithelial proteins that were not detected at the transcriptional level by RNA-Seq. **(i)** Heatmap depicting the expression level of the intestinal proteins not detected at the transcriptional level in the different types of cells in breast tissue previously identified by *Tabula Muris*. **(j)** Violin plot showing the quantification of the data presented in S2h Fig. Underlying data for panels a, b, c, d and e can be found in S4 Data. Underlying data for panels f and g can be found in S7 Data. Underlying data for panels h, i and j can be found in S8 Data. Dataset available under the identifier PXD059639 at the ProteomeXchange Consortium.
(EPS)

S3 Fig (a) Pictures of small intestinal tissue sections obtained from 1-, 5-, 10- and 25-day-old animals and stained using H&E staining. **Note the development of the intervillus/crypt structure with age. (b)** Heatmap depicting the expression level of the individual genes included in the 5 zonation quintiles along the adult villus length (as defined by Moor and colleagues, cluster 1 representing the base of the villus and cluster 5 the tip of the villus [3]) in 1-, 5-, 10- and 25-day-old crypt/intervillus area and villi samples. **(c)** Biplot displaying the top 50 driver genes for the 1st and 2nd dimension of the PCA plot shown in Fig 3b. Black/blue names represent genes that were identified as DE/non-DE between pre- and post-weaning crypts or villi samples. 1-day-old (orange dots), 5-day-old (green dots), 10-day-old (blue dots) and 25-day-old (pink dots). **(d)** Volcano plots showing differential gene expression between 1- and 5-day-old (top), 5- and 10-day-old (middle) and 10- and 25-day-old (bottom) crypt samples. Blue and red dots indicate genes statistically (*P*adj < 0.05) upregulated (|log_2_FC| > 1) in the younger and older animals, respectively. Gray dots indicate nonsignificantly and/or nondifferentially regulated genes. **(e)** Volcano plots showing differential gene expression between 1- and 5-day-old (top), 5- and 10-day-old (middle) and 10- and 25-day-old (bottom) villi samples. Blue and red dots indicate genes statistically (*P*adj < 0.05) upregulated (|log_2_FC| > 1) in the younger and older animals, respectively. Gray dots indicate nonsignificantly and/or nondifferentially regulated genes. **(f)** Temporal expression pattern of selected genes encoding amino acid/peptide transport molecules in the crypt (red) *versus* villus (blue) epithelium [3]. Median (lines) and median absolute deviation (MAD) (patches). **(g)** Heatmap depicting the expression level of genes known to be associated with EEC located either in the crypt (bottom) or villus (top) area in adult mice [69]. **(h)** Temporal expression pattern of EEC-specific hormone genes in the crypt (red) *versus* villus (blue) epithelium. Median (lines) and median absolute deviation (MAD) (patches). **(i)** Immunofluorescence images of small intestinal tissue sections of 10- and 25-day-old mice stained for IgA (red) (top) and corresponding image quantification (bottom). Counterstaining with DAPI (blue). For the image quantification, 20 fields (624,70µm x 501,22µm) along the proximal-distal axis were analyzed per animal. 3 animals were analyzed for each age. Asterisks show positive cells. Mann-Whitney test. **(j–m)** Box plots showing PROGENy pathway analysis (j,l) and NOTCH pathway analysis (k,m) in the crypt (j,k) and villi (l,m) samples at the different ages. Color code as in S3c Fig. ANOVA with Tukey-HSD post-hoc-test. Underlying data for panels c, d, e, f, g, h, j, k, l and m can be found in S9 Data. Underlying data for panel i can be found in S6 Data. Dataset available under GSE283142.
(EPS)

S4 Fig (a) Biplot displaying the top 50 driver genes for the 1st and 2nd dimension of the PCA plot shown in Fig 4b. 1-day-old (orange dots), 5-day-old (green dots), 10-day-old (blue dots) and 25-day-old (pink dots). **(b)** Expression level of the top 50 genes (by adjusted *p*-value) differentially regulated in each part *versus* the 2 other parts of the small intestine of 25-day-old animals plotted against the expression level in the corresponding part of 1-, 5- and 10-day-old animals. **(c)** Volcano plots showing differential gene expression between proximal and medial (left) and medial and distal (right) of 1-, 5-, 10 and 25-day-old animals (top to bottom). Blue and red dots indicate genes statistically (*P*adj < 0.05) upregulated (|log_2_FC| > 1) in the most proximal and most distal parts, respectively. Gray dots indicate nonsignificantly and/or nondifferentially regulated genes. **(d)** Volcano plots showing differential gene expression between 1- and 5-day-old (left), 5- and 10-day-old (middle) and 10- and 25-day-old (right) proximal, medial and distal samples (top to bottom). Blue and red dots indicate genes statistically (*P*adj < 0.05) upregulated (|log_2_FC| > 1) in the younger and older animals, respectively. Gray dots indicate nonsignificantly and/or nondifferentially regulated genes. **(e)** Dot plot showing the top 3 enriched GO terms (by adjusted *p*-value) for the DE genes downregulated with age in the proximal, medial and distal parts (left to right). The number of DE genes associated with a GO term for each comparison is indicated in brackets. **(f)** Dot plot showing the top 3 enriched GO terms (by adjusted *p*-value) for the DE genes upregulated with age in the proximal, medial and distal parts (left to right). The number of DE genes associated with a GO term for each comparison is indicated in brackets. Underlying data for panels a, c, d, e and f can be found in S10 Data. Underlying data for panel b can be found in S11 Data. Dataset available under GSE283143.
(EPS)

S5 Fig (a–d) Quality control and filtering criteria of the single cell dataset. (a-b) Violin plots showing the feature counts, gene counts, and percentage of mitochondria genes before (a) and after (b) nFeature and nCount filtering. (c) UMAP plot displaying the different clusters obtained after nFeature and nCount filtering (top) and violin plot showing the percentage of mitochondria genes in each of the clusters (bottom). Cluster 2 and 11 (red) were not included in the further analysis due to a high mitochondrial gene count. (d) Violin plots showing the feature counts, gene counts, and percentage of mitochondria genes after cluster filtering. Uninfected 1-day-old, orange; uninfected 5-day-old, green; 5-day-old infected at birth with *S.* Typhimurium, turquoise, uninfected 10-day-old, blue; uninfected 25-day-old, pink. **(e)** UMAP plots displaying the cells recovered at each time point. Same color code as in S5a Fig **(f)** Number of cells recovered per cell type and per time point after filtering. **(g)** Heatmap depicting the top 5 transcription factors (TF) per cell type predicted by DoRothEA in 1-, 5-, 10- and 25-day old animals. **(h)** Graphical representation of the top 15 transcription factors (TF) identified by DoRothEA for 1- (orange), 5- (green), 10- (blue) and 25-day-old (pink) absorptive enterocytes (top), Goblet/Paneth (middle) and enteroendocrine cells (bottom). colored cells depict transcription factors predicted by DoRothEA for each age group. White cells represent TF that were not detected by DoRothEA. **(i)** Upset plots showing the number of DoRothEA-predicted transcription factors specific to one or more age group for the absorptive enterocyte (top left), enteroendocrine (top right) and goblet/Paneth clusters (bottom). Only interactions with 5 and more TFs are shown. **(j)** Number of cells per absorptive enterocyte subcluster and per time point. **(k)** UMAP plots showing the expression level of genes previously found to be associated with post-weaned crypts (left) and post-weaned villi (right). **(l)** UMAP plots showing the expression level of genes previously found to be associated with post-weaned proximal (left) and post-weaned distal (right) samples. **(m)** UMAP plots showing the results of the pseudotime analysis for the 1-day-old (top left), 5-day-old (bottom left) and 10-day-old (top right) samples using Slingshot. Underlying data for panels g, h and i can be found in S13 Data. Underlying data for panels k and l can be found in S15 Data. Dataset available under GSE284074.
(EPS)

S6 Fig (a) Number of cells per goblet/Paneth subcluster and per time point. **(b)** UMAP plots showing the expression level of genes previously found to be associated with post-weaned crypts (left) and post-weaned villi (right). **(c)** Number of cells per EEC subcluster and per time point. **(d)** Frequency of the 7 subtypes of EECs per time point. Subcluster color code as in Fig 5i. Underlying data for panel b can be found in S15 Data. Dataset available under GSE284074.
(EPS)

S7 Fig (a–e) Volcano plots showing differential gene expression between 5-day-old uninfected and *Salmonella*-infected (a) absorptive enterocytes, (b) goblet/Paneth, (c) EECs, (d) stem and (e) TA cells. Blue and red/purple dots indicate genes statistically (*P*adj < 0.05) upregulated (|log_2_FC| > 1) in the uninfected and infected samples, respectively. Purple dots indicate genes uniquely differentially regulated upon infection by one cell type, whereas red dots indicate gene differentially regulated by multiple cell types. Gray dots indicate nonsignificantly and/or nondifferentially regulated genes. Underlying data for panels a, b, c, d and e can be found in S18 Data. Datasets available under GSE284074.
(EPS)

S1 Data Raw data used to generate the panels in Figs 1b–1f, 1i, 1j, S1a–S1f, S1j and 7c, 7d. (XLSX)

S2 Data Raw data used to generate the panels in Fig 1g, 1h. (XLSX)

S3 Data Raw data used to generate the panels in S1g–S1i Fig. (XLSX)

S4 Data Raw data used to generate the panels in Figs 2c–2g, 2i, 2j and S2a–S2e. (XLSX)

S5 Data Raw data used to generate the panels in Fig 2b. (XLSX)

S6 Data Raw data used to generate the panels in Figs 2h and S3i. (XLSX)

S7 Data Raw data used to generate the panels in S2f, S2g Fig. (XLSX)

S8 Data Raw data used to generate the panels in Figs 2a and S2h–S2j. (XLSX)

S9 Data Raw data used to generate the panels in Figs 3b–3i, S3c–S3h and S3j–S3m. (XLSX)

S10 Data Raw data used to generate the panels in Figs 4b, 4f, 4h–4k, S4a and S4c–S4f. (XLSX)

S11 Data Raw data used to generate the panels in Figs 4d, 4e and S4b. (XLSX)

S12 Data Raw data used to generate the panels in Figs 5b, 5e, 6c and 6j. (XLSX)

S13 Data Raw data used to generate the panels in S5g–S5i Fig. (XLSX)

S14 Data Raw data used to generate the panels in Figs 5d and 6b and 6k. (XLSX)

S15 Data Raw data used to generate the panels in Figs 5f, 5g, 6d, S5k, S5l and S6b. (XLSX)

S16 Data Raw data used to generate the panels in Fig 5j–5l and 5o–5q. (XLSX)

S17 Data Raw data used to generate the panels in Fig 6g, 6h. (XLSX)

S18 Data Raw data used to generate the panels in Figs 7a, 7b, 7f, 7g, and S7a–S7e. (XLSX)

## Abbreviations

DE: differentially expressed
EGFR: epidermal growth factor receptor
FC: fold change
FCS: fetal calf serum
FDR: false discovery rate
GF: germ-free
GO: gene ontology
LC: liquid chromatography
LCM: laser capture microdissection
LFQ: Label free quantification
LRT: likelihood ratios test
MAD: median absolute deviation
MAPK: mitogen-activated protein kinase
MS: mass-spectrometry
NPL: neuraminate pyruvate lyase
PCA: Principal component analysis
SPF: specific pathogen-free
TA: transit amplifying
TF: transcription factor
TGF: transforming growth factor
UMAP: Uniform Manifold Approximation and Projection
WAP: whey acid protein
WGA: wheat germ agglutinin.
