# Supplementary material for: Developmental factors drive the compartmentalized and discontinuous maturation of the small intestinal epithelium during the early postnatal period
Source: PLoS Biol. 2026 Aug 3;24(8):e3003212. doi: 10.1371/journal.pbio.3003212 (PMC13460745; doi:10.1371/journal.pbio.3003212)
Supplement: Supplementary file 1 [file pbio.3003212.s029.pdf]

Point-to-point response

Editor

As you will see, the reviewers find the conclusions interesting, but they also raise several issues that would need to be addressed before we can consider the manuscript for publication. Reviewer 1 thinks that the paper should focus on temporal analysis of the key villus zonated genes, describe the crypt and villus dynamic expression of the main EEC genes of the dataset, and analyse the correlation between the transcription signature of each segment at each time point at D25 to determine whether or not all segments mature transcriptionally at the same rates or there are segmental delays, among other issues. Reviewer 2 thinks the data on how the Notch pathway is altered in the intestinal stem cell niche should be included, compare gene expression among different developmental stages postnatal of germ-free mice, and show the expression of Defa or Reg3a, 3b or 3g genes to be able to determine whether Paneth/Goblet cells or enterocytes mature and start to express antimicrobial genes. Reviewers 3 and 4 have very similar views and note that some of the claims are not sufficiently supported by the evidence presented and suggest additional analyses to confirm them. In addition, Reviewer 4 thinks the cell viability issue should be addressed by optimising the experimental setup and perform a pseudotime analysis to compare age-dependent differentiation trajectories.

We appreciate the editor's comment and have modified the manuscript addressing all points raised by the reviewers (see point-to-point response below).

Reviewer #1 (Shalev Itzkovitz)

General comment: The manuscript provides a comprehensive resource for exploring neonatal maturation of the mouse intestine. It combines bulk transcriptomics, proteomics, LCMseq and scRNAseq at key time points before and after weaning. The experiments and analyses are generally well performed. It is hard to obtain a clear take-home message from the paper, but it will be useful to the community as a resource. The paper can be improved in several aspects in a revision:

We are grateful for the overall positive judgement and agree that the data presented have a strong resource character. We have addressed all specific points raised as described below.

Major comments

1. The paper focuses on epithelial lineages, what about stromal and immune lineages? At what stages are macrophages and lymphocytes established? Are there temporal variations in endothelial and fibroblast lineages?

We completely agree with the reviewer that a deeper insight into age-dependent changes of other stromal and immune cell types is interesting and important, and, in fact, this aspect is a major focus of the research in our group. We have previously studied age-dependent aspects of the intestinal epithelial and dendritic cell compartment and the establishing enteric microbiota in our group (for example Torow et al., Immunity 2023; Zhang et al., J. Exp. Med., 2022; van Best et al., Nat Commun., 2020; Fulde et al., Nature 2018; Torow et al., Nat Commun., 2015, Pott et al., PLoS Pathogens, 2012, Menard et al., J. Exp. Med. 2008). We are currently performing additional studies that address age-dependent changes in intraepithelial lymphocytes (Pes et al., unpublished), innate lymphoid cells (Ullrich et al., unpublished), lamina propria T lymphocytes (Al Bounny et al., unpublished), hepatic T lymphocytes (Stange et al., in preparation) and hepatic myeloid cells (Schwentker and Brück et al.,

under review) that will be published soon. In the present study, we however focused on intestinal epithelial cells and, given the cellular heterogeneity of primary epithelial cells and the technical challenges to study them, we believe that the addition of other cell types is beyond the scope of the current manuscript.

2. The paper focuses on crypt vs. villus, however the villus is a highly heterogeneous structure composed of zonal enterocytes (PMID 30270040, 39112711), and secretory cells (34031373). At what stages are these villus zonal sub-populations established? The authors should focus on temporal analysis of the key villus zonated genes.

*The reviewer raises an important point, and we agree that insight into the establishment of villus epithelial zonation after birth is of fundamental interest in respect to many important epithelial cell functions such as digestion, substrate transport, metabolism, innate immune defence and innate immune recognition. We have therefore re-analysed our dataset superimposing the reported marker genes of the different zones (i.e. cluster 1-5) of the villus epithelium reported by Moor et al., 2018 as suggested (**new Fig. 3c and new Fig. S3b**). The results are consistent with the results of the Moor et al. study in respect to adult animals and our analysis now identifies crypt-villus zonation also in preweaning mice although to a somewhat lower degree (page 20, lines 19-23- and page 21, line 3-4). The studies on epithelial crypt-villus zonation mentioned by the review are cited in this paragraph (page 20, line 21).*

3. One of the most interesting cellular populations in the gut are enteroendocrine cells, which exhibit strong zonation both along the crypt-villus axis and across intestinal segments (PMID 30712869, 30038251). The authors should describe the crypt and villus dynamic expression of the key EEC genes in their temporally-resolved dataset.

*We agree that enteroendocrine cells are functionally important and heavily understudied. As requested, we extended our analysis of the postnatal maturation of this epithelial cell type in respect to crypt-villus localisation and proximal-to-distal segmentation taking advantage of the EEC key marker genes established recently by Beumer et al., 2018 and Gehart et al., 2019. Our results confirm the demonstrated crypt- and villus-preference of certain EEC subtypes in adult mice and suggest a similar presence of most EEC subtypes across ages with a somewhat less defined distribution in the crypt and villus region (**new Fig. 3g** and page 21, line 6-8). Interestingly, the distribution of the different EEC subtypes is largely similar in the different gut segments throughout postnatal development (**new Fig. 4j** and page 24, line 5-7). This is consistent with our scRNA analysis that indicates the presence of the EECs subtypes throughout postnatal development (Fig. 6k). This represents a notable difference to absorptive and goblet Paneth cells (Fig.5d and 6b). Moreover, cell type marker genes including marker genes for EECs were included in Fig. 1-4 (**new Fig.1i**, **new Fig. 2g**, **new Fig. 3g**, **new Fig. 4j**).*

4. Segmental maturation - Figure 4 - the authors should compute the correlation between the transcriptomic signature of each segment at each time point with that at D25 to determine whether all segments transcriptomically mature at the same rates, or whether there are segmental delays.

*As suggested by the reviewer, we correlated the expression level of differentially expressed genes of the proximal, middle, and distal segment in 25-day-old mice with the expression level in the proximal, middle and distal segment of 1-, 5-, and 10-day old mice (bulk RNA Seq) (**new Fig. 4e-f**). The results reveal a weak correlation of gene expression in the proximal segment of preweaning mice but no correlation in the middle and distal part of preweaning mice consistent with a proximal-to-distal developmental wave (page 23, line 1-8). These results indicate a developmental segmental delay of the middle and distal segments of the intestine during the postnatal period.*

Minor comments:

1. Throughout the paper, dot plots of enriched functions are too cluttered and are hard to comprehend. The authors should focus on the most stringent pathways and rotate the plots so that the pathways are horizontal (rather than diagonal). They can provide the complete set of enriched pathways in supplementary tables.

As suggested, we reduced the number of GO terms in the respective figure panels (e.g. Fig. 3h and i, Fig. 4h, Fig. 5e, Fig. 6c, Fig. S4e and f, all panel numbers in the revised manuscript) showing only the most stringent pathways and rotated the figure panels to enhance the clarity and readability. The complete sets of identified GO terms are indicated in the respective Supplemental Tables as suggested.

2. Fonts in some figures are too small - e.g. Figure 5e,g,i. Better to show representative genes from each cluster with larger font.

We agree that the gene names in the Fig. 5 e, g, l, of the former manuscript version were hard to read without being able to zoom in on a digital file. The main reason to show these heatmaps was to illustrate the expression of genes differentially expressed between specific cell clusters determined in an unsupervised fashion at the different postnatal time points. We omitted the gene name description in the figure panels, added in some panels representative genes in bigger fonts, and provides the complete gene list in the corresponding Supplemental Tables as suggested.

3. Figure 2b - add Spearman correlation values and pvalues between RNA and proteins.

We added the requested information as suggested (Fig. 2b).

4. Proteome analysis - do the authors identify pancreatic proteins? Such as trypsin, amylase and lipase?

As suggested, we analysed the expression of pancreatic lipase (PNLIP), pancreatic lipase-related protein 1 (PNLIPRP1), pancreatic lipase-related protein 2 (PNLIPRP2), and trypsin 2 (PRSS2) protein in our proteome dataset. Amylase and trypsin 1 were not detected. We show the results to the reviewer in the point-to-point response since we believe it does not directly add to the data shown in figure 2.

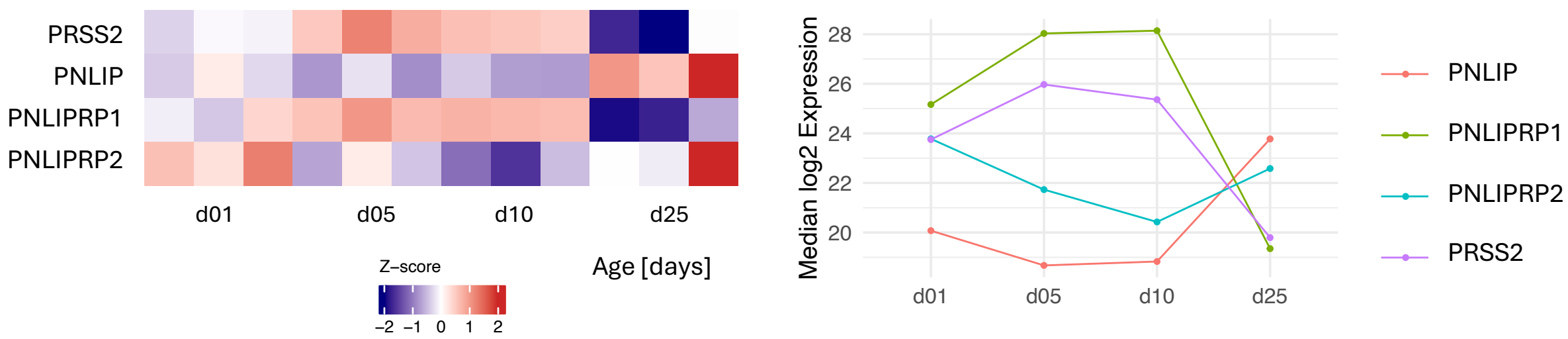

Reviewer #2

General comment: In the manuscript "Developmental factors drive the compartmentalised and discontinuous maturation of the small intestinal epithelium during the early postnatal period", Schöneich, Dupont, and colleagues investigated how the intestinal epithelial cells in different regions change their characteristics over time in the postnatal period, utilizing substantial transcriptomic and proteomic data analysis. These studies provide new insights into the organization and development of intestinal epithelial cells during this specific period. Notably, they also demonstrate how the influence of microbiota differs during the early pre-weaning period; gene expression was not highly affected by microbiota during this period. Overall, the paper's new finding is interesting and relevant

to the PLOS Biology readership. However, some concerns and questions need to be addressed to strengthen their conclusions.

We thank the reviewer for the overall positive judgement. We have addressed all specific points raised as described below.

Major comments

1.The authors show that at post-weaning, the alteration of TGF-beta, MAPK, and PI3K signaling coincides with the upregulation of stem cell signature genes. Those alterations of the pathways may link to the maturation of Paneth cells and promote the production of antimicrobial peptides.
To gain insight into how each cell type is affected by these signaling alterations, I recommend displaying the signaling pathway in crypt cells and villus cells (or at the single-cell level, if possible). Since the Notch pathway is also one of the essential pathways for the intestinal stem cell niche, which interacts with JAK-STAT to influence secretory vs absorptive fate, it would be beneficial to include data on how the Notch pathway is altered, providing an overview of the essential signaling.

*As suggested, we performed the signalling pathway analysis also for the crypt and villus epithelial (LMD-based) transcriptome data set (new Fig. S3g-j). Also, we added an analysis of the Notch pathway in Fig. 1 (as **new Fig. 1e**, bulk RNA Seq, age kinetic of total IECs) and Fig. S2 (as **new Fig. S2d**, proteomics, age kinetic of total IECs). Since the Notch signalling pathway is not included in progeny analysis, a separate analysis was performed as described in the material and methods section.*

2a. The authors demonstrated that developmental rather than microbial factors drive global transcriptome changes during the postnatal period. To strengthen this conclusion, authors should compare gene expression profiling among d01, d05, and d10 in germ-free mice, as they did in SPF mice in main Fig. 1c.

*As suggested, **new Fig. S1g** now shows volcano plots comparing gene expression between germ-free mice each for day 1 versus 5, and 5 versus 10 days after birth. This finding is described in the results section (page 17, line 3-4).*

2b. Moreover, a previous study has reported that colonization by microbiota just before weaning (day 17) induces Paneth cell differentiation in intestinal epithelial cells (PMID: 36473468, DOI: 10.1016/j.immuni.2022.11.003). Thus, there is a possibility that microbiota influences the differentiation and maturation of Paneth cells at the late stage of neonatal (2-3 weeks old) and after weaning. In order to clarify how microbiota contributes to Paneth cell differentiation at different neonatal stages, it would be beneficial to present gene expression profiling from germ-free mice at day 25 and compare it with that of SPF mice on the same day, if available. Otherwise, it might be better to add 'early' to the first subtitle of the result, 'Developmental rather than microbial factors drive global transcriptome changes during the XXX postnatal period.'

We understand the reviewer's point and agree that the analysis of older GF mice would be interesting to study the influence on Paneth cell differentiation. Unfortunately, due to a shortage of the number of germ-free animals available to us, we had to focus and decided to focus on the early time points after birth, the main topic of the study.

To respond to the reviewer's comment, we modified the subtitle as suggested (page 15, line 2-3). Also, we cited the indicated work by Kim et al., 2022 and discuss a possible influence of the enteric microbiota on Paneth cell maturation during late infancy (2-3 weeks of age) in the revised version of the manuscript (page 29, line 12-16).

Although the pace of Paneth cell differentiation during late infancy may still dependent on stimuli of the enteric microbiota, we would like to mention the work by Pütsep et al., (J. Biol. Chem, 2000) who compared Paneth cell-produced antimicrobial peptides between adult germ-free and SPF mice on the peptide level (mass spectrometry) and antimicrobial activity level (activity testing in a bacterial growth assay) and found a largely similar spectrum as well as amount of the proform and mature form of Paneth cell-derived α-defensins (PMID: 11010975).

3. In Figs. 5 and 6, the authors demonstrate how each type of cell undergoes alterations in gene expression and responses during the postnatal period using single-cell RNA sequencing (scRNA-seq). However, they do not annotate transit-amplifying cells (TA cells), which have distinct features and are considered an independent cell type. It would be better to annotate TA cells as a separate population and analyze them independently with other cell types, such as stem cells and other differentiated cells (enterocytes, tuft cells, Enteroendocrine cells, and Paneth/goblet cells).

To address the reviewer's point, we have reanalysed our scRNA Seq dataset from 1-, 5-, 10-, 25-day-old mice using TA cell marker genes defined previously (Haber et al., 2016, see page 11, line 22-26). We defined TA cells within the stem cell cluster as indicated in the revised UMAP (**updated Fig. 5a**) and the marker gene expression analysis (**updated Fig. 5b**). Furthermore, TA cells were added as cell type to all supplemental panels (**updated Fig. S5f and g**) and the manuscript text accordingly (page 24, line 14).
We also added TA cells to the heat maps that show the expression of cell type marker genes/proteins across age on the transcriptional (**new Fig. 1i**) and proteome levels (**new Fig. 2g** and **new Fig. 2h**, only detected marker proteins), along the crypt-villus zones (**new Fig. 3f**), in the different segments (**new Fig. 4i**) and after *Salmonella* infection (**updated Fig. 7f**).

4.The authors demonstrate the maturation of Paneth cells, goblet cells, and enterocytes over time, along with the expression of antimicrobial peptides, as shown in Figs. 1-4.
Since this finding is key to this paper, it would be beneficial to show the expression of Defa genes or Reg3a, 3b, or 3g genes, which were identified as altered genes in Figs. 1-4 over time.
By adding this data to the heat map for Paneth cells, goblet cells, or enterocytes in Fig.5e and g, it would also make it much more straightforward to determine whether Paneth/Goblet cells or enterocytes mature and start to express antimicrobial genes. Otherwise, the possibility that the differential gene expressions of antimicrobial peptides arise from the altered proportions of these cells which currently cannot be excluded.

*As suggested, we have specifically analysed the expression of antimicrobial proteins and peptides such as Reg3α, β and γ as well as α-defensins, and lysozyme (**new figure 1j**). We also included the cathelicidin CRAMP (gene name CAMP) that shows constitutive intestinal epithelial expression exclusively during the postnatal time window as we have previously demonstrated (Menard et al., J. Exp., Med., 2008, PMID: 18180308). Similarly, we also show the expression of antimicrobial peptides and proteins on the protein level (**new Fig. 2i**) and after Salmonella infection (**new Fig. 7c**). However, we prefer to show these results in separate figure panels since former Fig. 5e and g (now Fig. 5d and Fig. 6b) show genes selected in an unsupervised fashion based on cluster-specific gene expression.*

5.In Figs. 5d and e, it would be better to characterise each cluster of absorptive enterocytes. Showing genes, such as Reg1, Slc2a2, and Ada, that are preferentially expressed in each zone along the crypt-villus epithelial axis will help to define the clusters. Information described in PMID 30270040 will be helpful.

Figure 5 has been significantly improved. As recommended by reviewer #3 (point 10B), we performed a trajectory inference (pseudotime) analysis on absorptive enterocytes and goblet/Paneth cells and

*identified trajectories during the pre- and postweaning period (**new Fig. 5h, new Fig. 5m, new Fig. S5m, new Fig. 6f**). Notably, the trajectory of absorptive enterocytes largely follows the crypt-villus axis (**new Fig. 5f**), whereas the trajectory of the preweaning (i.e. before mature Paneth cells arise) goblet/Paneth cell cluster ends at areas of maximal Zg16 (goblet cells) expression (**new Fig. 6f**). Genes differentially expressed along the different trajectories and the associated transcription factors (DoRothEA) were identified and are shown in **new Fig. 5j and l, new Fig. 5o and q, and new Fig. 6g and h**. The new results are discussed in the manuscript text (page 25, line 22-26; page 26, line 1-6 and line 13-15). Figure 5d and e of the former version of the manuscript have been moved (now Fig. 5c and d and Fig. 6a-b) in the revised version of the manuscript. Genes depicted in the heatmap (Fig. 5d and Fig. 6b) were selected by an unbiased way representing the top 8 DE genes per absorptive enterocyte or goblet/Paneth cell subcluster.*

6.In Figs. 5f and g, it would be better to define which cluster corresponds to Paneth cells or Goblet cells, since they are distinct and defined cell types. It would be possible to separate goblet cells and Paneth cells by defined markers, such as Ag16 and Lyz1.

This comment maybe based on a misunderstanding. We do present an expression analysis of Zg16 (presumably what the reviewer refers to as "Ag16") as marker for goblet cells and lysozyme (Lyz1, former version of the manuscript) as marker for Paneth cells in Fig. 6e (former version Fig. 5f). This analysis shows that these established goblet and Paneth cell marker genes (notably identified in the adult intestine) do not clearly separate a Paneth or goblet cell cluster in the neonatal intestine but reveal a common origin of both secretory cell lineages and a continuum of cells with a more pronounced Paneth or goblet cell expression profile. Please note that the neonatal epithelium lacks mature Paneth cells and that we have an underrepresentation of crypt-based Paneth cells of the adult sample in our analysis (see discussion, page 29, line 21-page 30, line 3). Although this of course precludes a clear assignment to either cell type, it most likely reflects the biology of the neonatal intestinal secretory lineage cell differentiation (see also our publication on the expression of antimicrobial peptide genes in the neonate host after Salmonella infection, PMID: 39793046).

Minor comments:

1.In Fig.3a, it would be nice if you could draw and show how the tissue was micro-dissected with a laser in the images.

*As suggested, we added histological images in Fig. 3 that illustrate how the tissue was micro-dissected with the laser (**new Fig. 3a**).*

2.In Fig.3c, dots representing Tdrp, Meltf, Mamdc4, Cyp2b10, and Igkc are exactly in the same position between Crypts and Villi volcanoes. Could you please check if there's an issue with the data processing?

We thank the reviewer for this comment. The similar position of the named genes is due to the threshold set for the analysis as described in the material and methods section (page 10, line 1-4). To allow overall comparability, the fold changes and adjusted p-values in each analysis were capped at maximum and minimum thresholds corresponding to the 0.01 and 99.9 percent quantiles (negative and positive fold-changes) and the 0.01 percentage quantile for adjusted p-values in the volcano plots. This methodology was employed to prevent a small number of extreme values from overshadowing the plots, thereby allowing a clearer visualisation and ensured consistent axis ranges across volcano plots. The corresponding Supplemental Table contain the raw, uncapped data.

3.In Fig. 1a, it is mentioned that no samples for SPF (P/M/D) from d25 were analyzed; however, in Fig. 4c, data for SPF (P/M/D) from d25 are shown. Please clarify.

We apologize for the mistake, SPF (P/M/D) samples were analysed. We corrected Fig. 1a accordingly.

4.At line number 10 on page 14, the authors discussed that the decrease in the transforming growth factor (TGF)-beta after weaning is most likely due to the loss of TGF-beta from breast milk. However, the TGF-beta signaling in epithelial cells can also be influenced by the surrounding cells of the crypts and villi, including mesenchymal cells, which secrete BMP proteins.
It would be better to tone down your discussion.

We thank the reviewer for his/her comment and modified the text to account for the possible influence of local BMP proteins by mesenchymal cells on the decrease of the TGFβ pathway after weaning (page 16, line 9-10). We also added a new reference (PMID 30683451) to highlight this issue.

5.Salmonella infection can alter the expression of genes involved in tight junctions in intestinal epithelial cells and their localization, thereby changing the susceptibility of the intestine to pathogens. Specifically, the gene expression of key tight junction proteins, such as E-cadherin, occludin, claudins, and ZO-1, is reported to change in adult intestinal epithelial cells from adult mice (PMID: 32329192_DOI: 10.1111/cmi.13211). Did you also see those changes in the transcriptome of the neonatal intestine?

*As suggested, we compared specifically the expression of tight junction proteins such as claudins, occludin, and ZO1-3 in the epithelium of Salmonella-infected neonate and age-matched healthy control mice (**new Fig. 7d**). The results reveal no global change in the expression of tight junction molecules upon infection although individual claudins are up- and downregulated (page 27, line 11-15). The difference might be explained by the different models, immortalised Caco2 cells in the Liu et al. study versus primary IECs in our study, as recently studied by Mikael Sellin (PMID: 32365138). The work by Liu et al., 2020 indicated by the reviewer, is cited in the revised version of the manuscript to account for the reviewer's point.*

Reviewer #3

General comment: The manuscript "Developmental factors drive the compartmentalised and discontinuous maturation of the small intestinal epithelium during the early postnatal period", by Schöneich et al. characterizes transcriptional and proteomic changes in the mouse small intestinal epithelium at various stages of neonatal development. The key findings described in the manuscript provide new insight into the region- and temporal-specific changes to the small intestine during development that could provide the basis for new studies regarding neonatal development and host-microbe interactions. The manuscript would be an informative study for readers but requires additional work - considerations are provided below.

We thank the reviewer for the overall positive judgement and have addressed the specific points raised. In particular, we would like to thank the reviewer for the suggestion to change the scRNA Seq data filtering, which we believe has much improved the downstream analyses in our manuscript.

Specific comments

1. P15 - Maternally acquired proteins in the pre-weaning epithelium revealed by proteome analysis
The authors' title for this section should be revised as this section highlights major changes in the

proteome of the small intestine epithelium between pre- (day 10) and post-wean (day 25) and between day 1 and day 5 (maternally acquired proteins). Given the robust changes in the proteome at both timepoints, the title should be changed to reflect the observation at both timepoints.

As suggested, we modified the title of this section to account for the proteome changes both between day 1 and 5 and between day 10 and 25 (page 18, line 2).

2. The authors claim that the change in proteome between day 1 and day 5 of the small intestinal epithelium is due maternal milk-derived proteins. This claim is supported by scRNA-seq data from breast tissue of Tabula Muris that shows breast luminal cells express genes of proteins identified by proteome analysis, but not RNA-seq. The aggregated expression of the 135 genes by UMAP and violin plot in luminal cells is an issue, as it obscures the degree to which these potentially maternally-derived proteins are transcriptionally expressed. Re-visualizing these data as a heatmap with all genes plotted against each cluster as a supplemental figure is recommended. In addition, these data are only correlative and do not provide sufficient evidence that the increase in proteins at day 5 are derived maternally from breast milk. As such, moving these scRNA-seq data as supplemental data would be appropriate.

*As suggested, we generated a heatmap of the differentially expressed genes to better illustrate their degree of transcriptional expression (**new Fig. S2i**). Also, the Tabula muris scRNA Seq dataset analysis was moved to the supplemental data as recommended (Fig. S2h-j).*

3. P18 - "Additionally, the formation of deeper crypts during the late postnatal period may affect the metabolic and transport capabilities of the post-weaning crypt epithelium, due to reduced contact with dietary substrates." This statement provides an explanation as to why the post-wean crypts are associated with a decrease in the lipid metabolism gene signature, but it is contradicted by similar decreases observed in post-wean villi (Fig 3e). As this explanation is speculative and the mirrored decreases in the villi likely reflect a general change in dietary milk, removing this sentence from the results is recommended.

As suggested, we removed the above cited sentence from the results section (page 21, line 22).

4. P18 - "Age-dependent compartmentalisation of gene expression along the crypt-villus axis". The authors use laser capture microdissection (LMD) to characterize the age-dependent changes in gene expression that occurs within the crypt/intervillus and villus regions. The authors identified broad transcriptomic changes in the expression of genes associated with host-defense, lipid and triglyceride metabolism, translation activity, stemness (Olfm4), and cellular turnover. Given that these broad changes in transcriptome are also associated with changes in epithelial cell subsets, the authors should expand upon this idea by providing quantified immunofluorescence staining of some key epithelial cell subsets (ie. stem cells (Olfm4), transit-amplifying cells (Mki67/Pcna, Paneth cells (Lyz1/UEA1), goblet cells (Muc2)) and/or candidate genes of interest (ie. Reg3a)).

*As suggested, we stained small intestinal tissue sections from each three 1-, 10-, and 25-day-old mice and stained for the epithelial cell type markers chromogranin A (enteroendocrine cells), mucin 2 (goblet and Paneth cells), lysozyme (Paneth cells), and Ki67 (proliferating TA cells) and quantified the number of positive cells per field. Examples of the immunofluorescence staining and the statistically evaluated quantification are shown (**new Fig. 2h**). These results illustrate age-dependent changes in the cell type composition of the small intestinal epithelium and validate our results (page 19, line 25-26, page 20, line 1-3).*

5. P19 - "In contrast, increased expression of the IgA transport molecule polymeric immunoglobulin receptor (pIgR) in both post-weaning crypt and villus epithelium was consistent with the increase of

IgA-secreting plasma cells occurring at weaning (Fig. 3c, Fig. S3b-c, Suppl. Table 6)" - The argument for an increase in the abundance of IgA-secreting plasma cells in the villus post-weaning not sufficiently supported as it is based solely on gene expression data of LMD-isolated villi. I would suggest additional corroborative data such as quantifying the number of B cells present within the pre- and post-wean villi.

*As suggested, we stained IgA^{+} plasma cells in small intestinal tissue sections of 10 and 25-day-old mice to illustrate their localisation along the crypt-villus axis. The results confirm the absence of plasma cells in the lamina propria in neonate mice. In adult (day 25) animals, plasma cells were detected (**new Fig. S3f**). These results are consistent with work by our (e.g. PMID 26195040, 36921575, and 37130522) and other groups (23965619 and 17056555).*

6. P19-20 "Transcriptional differences along the proximal-to-distal length of the small intestine". The authors demonstrate that the proximal, medial, and distal intestine undergo region-specific transcriptomic changes that are dependent on age. The authors further aggregate genes of similar expression pattern and label these as 'clusters' while describing their overall function based on GO term enrichment and the expression of key genes of interest. This section would benefit with more interpretation with reference to the study by Zwick et. al. titled "Epithelial zonation along the mouse and human small intestine defines five discrete metabolic domains" (https://eur02.safelinks.protection.outlook.com/?url=https%3A%2F%2Fdoi.org%2F10.1038%2Fs41556-023-01337-z&data=05%7C02%7C%7C0ff4a2579d6245ebe69408ddb4144769%7C5a6d5ee56edf4a26ba93f5872dbb9614%7C0%7C0%7C638864721836319329%7CUnknown%7CTWFpbGZsb3d8eyJFbXB0eU1hcGkiOnRydWUsIlYiOiIwLjAuMDAwMCIsIlAiOiJXaW4zMiIsIkFOIjoiTWFpbCIsIldUIjoyfQ%3D%3D%7C0%7C%7C%7C&sdata=Zquk1VyzgB9Wu0aIwrRBibDNH0rIivfqqyY20bCtKDU%3D&reserved=0). Zwick et. al. demonstrate that the small intestinal epithelium can be divided along the proximal-distal axis into 5 metabolic domains. It would be of interest to the field to determine if these region-dependent metabolic domains are present pre- or post-weaning.

*As suggested, the marker genes of the five adult gut segments (domain A-E) identified by Zwick et al. were projected on our dataset of the proximal, medial and distal epithelial transcriptome of 1-, 5-, 10-, and 25-day-old mice both in an aggregated form as expression of all marker genes (**new Fig. 4c**) and for the identified genes individually (**new Fig. 4d**). The analyses confirm the segmental expression of the profile of domain A to E in our adult day 25 dataset, although, as expected, the five domains do not perfectly fit onto our three parts of the intestine. They further clearly show that the preweaning intestinal epithelium does not exhibit a similarly strong adult-type segmentation. Individual genes of domain A-E do not show segmental expression along the proximal-to-distal epithelium of 1-, 5-, or 10-day old mice (page 22, line 25-page 23, line 4).*

7. P20 - "Age- and cell type-specific developmental trajectories revealed by single cell transcriptional analysis". P10 & Suppl. Fig 5 - For scRNA-seq QC metrics, the authors state that UMIs with >25% mitochondria gene expression were removed. With respect to enterocytes and intestinal stem cells, these two cell subsets by scRNA-seq and other methods have been well characterized to generate more mitochondria due to their metabolic activity. As such, it is generally not recommended filter IECs based on mitochondrial gene expression. The authors need to revisit their scRNA-seq analysis to assess if this cutoff is removing key epithelial cells from their analysis with respect to the day 10 and day 25 samples.

*As recommended, we removed the mitochondrial gene expression filter and re-analyzed our scRNA data set. The modified stepwise filtering protocol is described in the material and methods section of the manuscript (page 11, line 13-16) and illustrated in the **new Fig. S5a-e**. The modification of the*

protocol notably increased the number of cells in the day 10 but in particular in the day 25 sample allowing a more detailed analysis of the postweaning epithelium. We thank the reviewer for this suggestion, which we believe has very significantly improved our study.

8. Figure 5 & Suppl. Fig 5 - Based on the heatmaps (Fig 5E, G) QC data presented, the authors post-wean (day 25) sample captured a critically small fraction of crypt/villus enterocytes (10-30% compared to other timepoints) along with decreased unique features/genes expressed compared to other timepoints. In addition, no stem cells were captured, while there is a 2:1 ratio of enterocytes to secretory cells. This composition of cells captured for this timepoint contradicts other parts of the manuscript which suggest post-weaning intestinal crypts become more defined and develop Paneth cells (ie. stem cells and Paneth cells should be captured). These limitations raise concerns regarding the quality of the data for this sample and suggest the findings based on this sample should be removed from the manuscript or at minimum the caveats of these conclusions should be clearly discussed in the results or discussion.

*As suggested in point 7 of reviewer #3 we modified the filtering protocol of the scRNA Seq dataset (**new Fig. S5a-e**). Thereby we significantly increased the number of analysed cells in the day 25 sample to 4609 (compared to 4979, 2784, and 2306 cells in the day 1, 5, and 10 sample, respectively). This also increased the enterocyte to secretory cell ratio (**adapted Fig. S5f**). All subsequent calculations in Fig. 5 and 6 were re-analysed based on this modified protocol (and in part modified as recommended by the reviewers). We would also like to stress out that we had already initially run two day 25 samples and combined the data for the analysis. Based on the much-increased number of day 25 cells in the analysis and the improved enterocyte to secretory cell ratio, we decided to keep the improved dataset in the manuscript.*

Our epithelial cell isolation protocol for the scRNA Seq analysis was optimised for pre-weaning samples. We had decided to use this protocol since it allowed the best viability of all epithelial cells (including stem cells and Paneth cell precursors) from neonatal mice. However, the reviewer is correct that this protocol leads to an underrepresentation of crypt-based cell types such as stem cells, Paneth cells and TA cells in the day 25 sample. We are aware of this issue and describe this important limitation of the study in the results and discussion section (page 29, line 21-page 30, line 3 and page 24, line 15-17).

9. P20 - "Notably, epithelial cells from adult mice showed an overall lower viability". For the authors' scRNA-seq experiment, they should clarify whether this is based on viability by flow cytometry or scRNA-seq analysis and provide the data.

This statement was based on the reduced number cells from adult (25-day-old) mice entering the analysis using a filtering protocol that included a 25% mitochondrial gene count threshold in the original version of the manuscript. As recommended (see point 7, reviewer #3), we now modified this protocol allowing us to analyse in the revised version of the manuscript a similar number of cells for all ages (4979, 2784,2306, and 4609 cells in the day 1, 5, 10 and 25 sample, respectively). To account for the improved analysis, we modified this sentence (page 24, line 15-17).

10a. P20 - "We initially focussed on the effect of age on epithelial cell type differentiation since, although studied in the adult host, little is known about regulatory circuits that drive epithelial cell differentiation during the neonatal and infant period." Highlighted spelling error.

We thank the reviewer and correct the spelling error (page 24, line 18-20).

10b. The authors use DoRothEA to predict transcription factor (TF) activity that drive epithelial cell differentiation during neonatal development. While the authors identified prospective TFs that may

play a role in driving lineage commitment of enterocytes and secretory epithelial cells, it remains unclear if these TFs drive cell type-specific functions or lineage commitment, given that these associations are presented with 'terminally' differentiated subsets. To better delineate this idea, performing trajectory inference analysis is key to determine where along the predicted differentiation trajectory these TFs are expressed (ie. are these TFs expressed in progenitor population at earlier timepoints or only in progenitor populations post-weaning?).

*We agree with the reviewer on the limitation of the previous analysis and discuss this issue in the manuscript (page 25, line 7-8). As suggested, we performed trajectory inference analysis (pseudotime) on the preweaning and postweaning scRNA Seq dataset of absorptive enterocytes and goblet/Paneth cells. We thereby identified cell type-specific differentiation trajectories (**new Fig. 5h, new Fig. 5m, new Fig. S5m, new Fig. 6f**). We then performed DE gene analysis and DoRothEA on the DE genes along the differentiation trajectories (**new Fig. 5j and l, new Fig. 5o and q, new Fig. 6g and h**).*

11. P21, Figure 5D/F - "A spatial dimension was added to the absorptive enterocyte subclusters by overlapping the gene expression signatures of crypt and villus epithelium previously identified by bulk RNA-Seq (Fig. 3) onto our single cell dataset (Fig. 5d). This allowed the identification of the gene signatures of single absorptive enterocytes along the crypt-villus axis and illustrated the continuous gradient of cell differentiation and functional diversity… A similar phenomenon was also observed for goblet/Paneth cells." The authors infer the trajectory of enterocytes and goblet/Paneth cells based on their previously identified crypt-villus axis gene signatures and defined markers of goblet and Paneth cells that illustrate a gradient of expression that progress through respective subclusters. A key limitation of this analysis is that the authors have separately subclustered enterocytes and secretory lineages when the differentiation of stem cells to enterocytes and secretory lineages occurs in tandem. Moreover, inferring trajectory based on expression gradients of defined genes/gene sets superimposed over subclusters is incorrect, as it assumes subclusters are defined and ordered on the same principles, when their defining genes could be unrelated to their lineage commitment. As such, the authors should perform trajectory inference analysis for each of their respective timepoints to better support this idea.

*As suggested, we performed trajectory inference analysis of the preweaning and postweaning absorptive enterocytes and goblet/Paneth cells. However, pseudotime analysis in our hands was unable to provide a meaningful result based on the stem cell plus absorptive enterocyte or stem cell plus goblet/Paneth cell cluster. Nevertheless, we believe that these trajectories provide insight in the gene expression profile along the continuous cell differentiation within cell clusters (see also point 10b, reviewer #3). We therefore subsequently performed an analysis of the DE genes and gene set-associated transcription factors (DoRothEA) along the 10 bins of each identified trajectory (**new Fig. 5 and 6**).*

12. P22 - "The global and cell type-specific gene response to enteric infection"

We corrected the indicated the sentence (page 26, line 23).

13. P22 - "UMAPs representing the scRNA-Seq of the uninfected (d05) and infected datasets (d05i) illustrated a marked decrease in the abundance of absorptive enterocytes in 5-day-old infected animals." Missing figure reference.

The figure reference (Fig. 7e in the revised manuscript) was added as suggested (page 27, line 18-21).

14. P22 - "DE gene analysis of day 5 uninfected and day 5 infected samples revealed upregulation of typical epithelial antimicrobial host response genes such as c-type lectins (e.g. Reg3g, Reg3b,

Reg3a)". The authors demonstrate that the pre-wean epithelium under steady-state does not express antimicrobial/host defense genes, but that these genes are upregulated post-wean (C-type lectins and defensins). Interestingly, these data suggest the neonatal epithelium has the potential to express host defense genes if required. Moreover, the scRNA-seq of infected and uninfected day 5 epithelium suggest that the increase in Reg3g/a expression with infection occurs in all epithelial lineages (Suppl. Fig 6). These data suggest that the neonatal epithelium may rely on c-type lectins in response to enteric infections, as opposed to defensins from Paneth cells that arise post-weaning. As such, the importance of investigating enteric infections in the neonatal epithelium could be emphasized and improved by expanding upon this distinction in this section of the results or within the discussion.

We completely agree with the interpretation of our findings by the reviewer and are grateful for the stimulating discussion. We would like to additionally mention epithelial expression of the cathelicidin CRAMP by the neonatal but not adult intestinal epithelium previously described by our group (Menard et al., J. Exp. Med. 2006). As suggested, we have expanded the discussion section to discuss the interesting topic of a constitutive versus inducible antimicrobial response in the neonate versus adult host (page 30, line 20-23).

15. P23 - "whereas absorptive enterocytes were associated with a higher cell division rate (e.g. "chromosome segregation", GO:0007059) and goblet/Paneth cells with a higher innate immune response orchestrated by pattern recognition receptors (e.g. "activation of innate immune response", GO: 0002218)." The authors perform differential gene expression analysis and GO term enrichment between uninfected and infected enterocytes and identify many differentially expressed genes and enriched GO terms (Suppl Fig 6a, Fig 6e). A limitation of this comparison as indicated by Fig. 6c is that differential expression compares 81 infected to 1339 uninfected enterocytes. Given the imbalance the number of enterocytes compared, the expression profile of infected enterocytes id likely not representative. This comparison should be removed.

As suggested, we have modified the filtering protocol of the scRNA Seq dataset and re-analysed the data (see point 7, reviewer #3). This allowed the analysis of a larger number of absorptive enterocytes also in the infected day 5 sample (now 294 infected and 2227 uninfected absorptive enterocytes) although neonatal infection still severely reduced the viability of absorptive enterocytes following isolation (Fig. 7e). This likely reflects a biologically relevant influence of infection-induced tissue inflammation on cell viability.

Although we agree that this caveat still represents a major limitation, which we discuss in the manuscript text (page 29, line 21-page 30, line 3), we believe that with the increased number of infected enterocytes, a comparative gene expression analysis still provides a meaningful insight.

16. P25 - "Many largely uncharacterised transcription factors were significantly associated with specific epithelial cell types in the neonate and await further analysis." The purpose/message of this paragraph in the discussion is unclear. The authors state the identified TFs that may drive neonatal epithelial differentiation and speculate that these TFs may play additional roles in restitution from inflammation and regulating various gradients in the small intestine. However, the importance of this idea with respect to neonatal development or intestinal physiology needs further exploration and explanation.

We completely agree with the reviewer that the discussion of the identified potential transcription factors in respect to epithelial cell differentiation and intestinal physiology is speculative and requires future investigations. However, as pointed out by reviewer #1 (general comment), our manuscript has a strong resource character, and we believe that the identification of age- and cell type-specific transcription factors may be of interest for the community and promote further analysis of their

potential role. To account for the reviewer's comment, we have moved the results to the supplemental figures (Fig. S5g-i).

Reviewer #4

General comment: The resulting molecular atlas offers novel insights into: 1. transcriptional changes in intestinal epithelial cells during the postnatal period driven by developmental factors, 2. non-transcriptional regulation of proteins between day 1 and day 5 after birth, 3. age-dependent transcriptional changes along the proximal-to-distal and crypt-villus axes, 4. cell type-specific single-cell transcriptional dynamics during postnatal development, 5. transcriptional alterations in specific cell types in response to infection. This study holds significant potential, as it provides a spatially resolved multi-omics resource for characterizing molecular changes within the mouse intestinal epithelium driven by developmental and microbial cues. However, several major issues need to be addressed to fully support the authors' claims.

We are grateful for the overall positive judgment of the reviewer and have carefully responded to the points raised (see below).

Major comments

1. Figure 1f: It is not clear how the authors evaluated the significance of correlations between the two sets of genes.

As suggested, we modified the text and describe in detail how the data correlation and significance calculation in Fig. 1g of the revised manuscript (Fig. 1f in the former manuscript version) was performed (page 10, line 5-12).

2. Figure 2: The proteomic changes between day 1 and day 5 appear to differ from the transcriptomic changes. The authors suggest that this discrepancy may be due to non-transcriptional regulation of proteins on day 5. However, the correlation between protein and gene expression levels on day 5 appears to be comparable to that of other time points (Figure 2b). This inconsistency should be carefully investigated.

We thank the reviewer for this comment, which may be due to a misunderstanding. Fig. 2b only shows the correlation of genes/proteins identified both, in the transcriptome and proteome dataset.

3. On page 20, the sentence "Notably, epithelial cells from adult mice showed an overall lower viability" is ambiguous. The authors should clarify what type of variability was measured and provide appropriate supporting evidence.

This statement was based on the reduced number cells from adult (25-day-old) mice entering the analysis using a filtering protocol that included a 25% mitochondrial gene count threshold in the original version of the manuscript. As recommended by reviewer #3 (see point 7, reviewer #3), we now modified this protocol allowing us to analyse in the revised version of the manuscript a similar number of cells for all ages (4979, 2784,2306, and 4609 cells of the day 1, 5, 10 and 25 sample, respectively). To account for the improved analysis and increase the clarity, we modified this sentence (page 24, line 15-17). See also point 9, reviewer #3.

4. The scRNA-seq data generated in this study appears to be of suboptimal quality. In particular, intestinal stem cells at postnatal day 10 and day 25 seem to be underrepresented, likely due to issues with cell viability. Although the authors have acknowledged this limitation, the depletion of intestinal

stem cells could significantly compromise the accuracy of differentiation trajectory analyses. Notably, the authors failed to demonstrate age-dependent differentiation trajectories starting from intestinal stem cells. Figure 5e and 5g do not clearly depict such trajectories. Additionally, the meaning of the subcluster ordering in the heatmaps is unclear. Given that intestinal stem cells have been successfully captured in other studies, the authors should more thoroughly address the cell viability issue by optimizing their experimental protocols. Furthermore, they should perform a pseudotime analysis to rigorously compare age-dependent differentiation trajectories.

*We appreciate the reviewer's comment which together with comments by reviewer #3 led us to re-analyse our dataset. Reviewer #3 (point 7) recommended to remove the 25% mitochondrial gene count filter, and this allowed a greatly increased number of absorptive enterocytes to be analysed. However, crypt-based cell types such as stem cells of the adult intestinal sample were still underrepresented. This is likely because our protocol was optimised to capture viable preweaning epithelial cells, the focus of this study. We discuss this limitation of our study in the discussion section (page 29, line 21, page 30, line 3). In addition, and as suggested, we successfully performed trajectory inference analysis and were able to identify differentiation trajectories both of preweaning and postweaning absorptive enterocytes and goblet/Paneth cells (**new Fig. 5h and m, new Fig. S5m, new Fig. 6f**). Also, we were unable to obtain meaningful data on a multicluster (including stem cells and absorptive enterocytes or stem cells and goblet/Paneth cells) dataset. Here the relative developmental trajectory distance between clusters may have by far outnumbered the trajectory distance within the clusters. In addition, we analysed the DE gene expression (**new Fig. 5j and o, new Fig. 6g**) and transcription factor signal (DoRothEA) along the differentiation trajectories (pseudotime) of both cell types (**new Fig. 5l and q, new Fig. 6h**).*

5. The scRNA-seq data lack biological replicates, which raises concerns about the reproducibility and robustness of the results presented in Figure 5 and 6. Given the suboptimal quality of the dataset, at least one additional biological replicate should be generated to support the conclusions. Alternatively, the transcriptional changes observed in Figures 1 and 3 could be recapitulated by aggregating single cells into pseudobulk profiles to validate the quality of the scRNA-seq data.

As described in the material and methods section (page 8, line 5-6), two independent day 25 samples were run and analysed independently. The results of the two day 25 samples were very similar and so all subsequent analyses were conducted with a pooled day 25 sample.

Minor comments:

1. Figure 1d, S1e, S2c: Y-axis labels should be provided.

The figure has been modified as suggested

2. Figure 1e, S2d: Representative genes should be highlighted.

All genes listed in the heatmap are indicated in the corresponding Suppl. Table.

3. Define "UMAP" thought the manuscript.

UMAP has been defined prior to the first use of the abbreviation in the manuscript (page 27, line 16-18).
