# Supplementary material for: Developmental factors drive the compartmentalized and discontinuous maturation of the small intestinal epithelium during the early postnatal period
Source: PLoS Biol. 2026 Aug 3;24(8):e3003212. doi: 10.1371/journal.pbio.3003212 (PMC13460745; doi:10.1371/journal.pbio.3003212)
Supplement: Supplementary file 2 [file pbio.3003212.s030.pdf]

Editor's comments:

Thank you for your patience while we considered your revised manuscript entitled "Developmental factors drive the compartmentalised and discontinuous maturation of the small intestinal epithelium during the early postnatal period" for publication as a Research Article at PLOS Biology. Your revised study has been evaluated by the PLOS Biology editors, the Academic Editor and the four original reviewers.

The reviews are attached below. You will see that while some of the reviewers are mostly satisfied with the revision, others raise several concerns that should be addressed before we can consider the manuscript for publication. **Reviewer 1** thinks a discussion should be added on developmental dynamics of stromal cells and immune cells and also include additional plots to track the changes in key enterocyte markers over time and explain how these relate to changes in diet/exposure to bacteria, among other requests. **Reviewer 2** has similar thoughts and finds controversial the interpretation that downregulation of Lrp2 after weaning maybe linked to the high metabolic demand imposed by the lipid-enriched milk diet of the newborn. This reviewer would like to see a revision of the discussion according to the presented data and consider the fact that Goblet and Paneth cells don't possess absorptive functions and reinterpret their results and points out that Fig. S2 and S3 are the same, thus please make sure that the correct Fig. S3 is uploaded in the new revision. **Reviewer 3** is satisfied, whereas **Reviewer 4** mentions that there is no attempt to optimise the single-cell isolation protocols, which are biased toward capturing viable preweaning epithelia cells and are suboptimal for isolating viable adult intestinal epithelial cells (points 4 & 5 of the previous review). Thus, this reviewer thinks that the comparative analyses across the developmental time points are limited. The reviewer would like to see a demonstration of the robustness and reproducibility of the conclusions drawn from these data. In light of the reviews, and after further discussion with the Academic Editor. we would like to invite you to revise one more time the work to address the reviewers' reports making sure that, at minimum, these points are thoroughly addressed:

1) scRNA-seq dataset quality control: a rigorous technical justification should be provided for the high mitochondrial content in the late-stage samples and clear evidence of reproducibility should be presented across biological replicates, to ensure that the dataset reflects biologically intact cells.

As requested, we analysed and provided evidence for the reproducibility and overall robustness of our scRNA analysis (see answer to reviewer #4).
First, we performed a comparative analysis of the absorptive enterocyte cluster between the two day 25 samples that were pooled in the analysis in the manuscript. The UMAP demonstrates strongly overlapping cell clusters. The genes differentially expressed between the lower and upper villus correlated strongly between the two day 25 samples. This suggests good reproducibility of our approach (see response to reviewer #4).
Second, we compared our day 1, 5, 10 and 25 samples with the day 10 and 24 samples recently published by Matsuki et al (Muc. Immunol., 2026). The results demonstrate that the genes differentially expressed between the lower and upper villus correlate strongly between our and the Matsuki analysis. By overlaying the crypt-villus zonation marker genes published by Moor et al (Cell, 2018) on the absorptive enterocyte cluster of our own and the dataset from Matsuki et al., we show a largely representative marker spectrum for our samples with the noted underrepresentation of crypt cells particularly in the day 10 and day 25 samples. Notably, the adult and to some extent also the neonatal absorptive enterocyte cluster of the Matsuki dataset

show a marked underrepresentation of villus cells. The lack of villus cells may explain the low number of cells with high mitochondrial gene count since anoikis and cell exfoliation are a natural process at the upper villus.

Other previous scRNA Seq studies on primary intestinal epithelial cells employed a high or no mitochondrial gene count threshold at all. For example, no threshold was used by Haber et al. (Nature, 2017), a 50% threshold was used by Wang et al. (J. Exp. Med., 2019), Tsang et al. (Gut Microbes, 2025) or Elmentaite et al. (Nature, 2021), a 30-35% threshold was used by Zwick et al. (Nat. Cell Biol., 2024) and Block et al. (Development, 2025), and a simple removal of mitochondrial RNAs without filtering cells was used by Kim et al. (Commun Biol. 2025) and some studies do not report the percentage of cells removed based on this threshold. As analysed and discussed in detail in the response to reviewer #4, some of these studies, including the Matsuki et al. study, either specifically isolated crypt epithelial cells, or likely removed epithelial cells with reduced viability (and thereby villus epithelial cells) prior to the scRNA Seq analysis (Matsuki et al., Muc. Immunol., 2026). Of note, cell anoikis (programmed cell death) represents a physiological aspect of the villus epithelium reflecting the rapid cell turnover and exclusion of these cells might therefore bias the analysis towards the crypt epithelium and remove biologically relevant cells. Finally, we would like to remind the editor and reviewers that the omission of a mitochondrial gene count threshold was requested by reviewer #3 during the first review round (reviewer 3, former point 7). However, we acknowledge a possible reduced quality of our day 25 dataset and discuss this in the limitations of the study paragraph (Page 30, line 21-page 31, line 3:
"Nonetheless, the isolation and analysis of primary small intestinal epithelial cells remain technically challenging. Firstly, detachment from the basal membrane, a prerequisite for the isolation and analysis of epithelial cells, reduces their fitness and together with the proapoptotic signals during infection-induced inflammation may reduce their viability [91]. Secondly, the anatomical organisation of the intestinal epithelium makes it difficult to isolate representative fractions of viable cells simultaneously from villi and crypts at different ages using the same protocol [92]. As our analysis focused on the early postnatal period, we prioritised villus cell over adult crypt cell coverage, which explains the underrepresentation of crypt-based stem, TA, and Paneth cells in our adult sample. However, the crypt epithelium of adult mice has been previously characterised [2,3]."

2) Improved temporal presentation of key markers: aggregated summaries should be replaced with high-resolution, gene-specific temporal plots for key antimicrobial, transporter, and hormone markers, enabling clearer links between molecular transitions and physiological milestones such as weaning and dietary changes.

See answer to point 2, reviewer #2. As requested, additional plots showing the median expression over time for the indicated antimicrobial, amino acid/peptide transporter, glucose transporter, lipid and purine metabolism genes were included showing the age- (Fig. 1d), microbiota- (Fig. S1j), zonal- (Fig. 3e and Fig. S3f), and segment-dependent (Fig. 4h) gene expression. Temporal changes are discussed in the manuscript text in the respective paragraphs as requested (for details see response to point 2, reviewer #2).

3) Refined narrative on metabolic transitions: the metabolic transition from the prenatal to post-weaning period should be clarified as a shift from a "primed/regulatory" state to an "active execution" of lipid metabolism, while noting that the metabolic profile observed in secretory cells likely reflects bioenergetic demand rather than genuine absorptive function.

As suggested, we refined the narrative on metabolic transition from the pre- to the post-weaning period.
Page 22, line 21-26 now reads:
"Also, the crypt/intervillus epithelium exhibited decreased expression of genes represented by the GO terms "regulation of lipid metabolic process" (GO:0019216) and "cellular lipid catabolic process" (GO:0016042) and a simultaneous increase of genes represented by the term "fatty acid metabolic process" (GO:0006631) at weaning, likely representing a shift from a "primed/regulatory" state to an "active execution" of lipid metabolism (Fig. 3c, h and i, Fig. S3c and d, Suppl. Table 9) [72]."

In addition, we would like to consider the paper as a Resource, thus we have changed the format to Methods and Resources.

We agree that our study should be considered a resource paper.

Reviewers' comments:

Rev. 1:
The authors have addressed my comments with new analyses and figures. Some of these are great and add value to the paper, while others should be improved for clarity:

1) Developmental dynamics of stromal cells and immune cells - the authors mention other manuscripts that explore these populations, this is ok, but the authors should mention this in the discussion as a future direction or refer to previous work.

As suggested we modified the text in the discussion as follows (page 32, line 25-page 33, line 8): "Also, the intestinal epithelium directly or indirectly interacts with other stromal, nervous and immune cells in the intestinal tissue and major changes in the cell type abundance, differentiation, activation and effector function have been described for epithelial cells but also for T and B lymphocytes (ref 15,90), dendritic cells (ref 71), lymphatic endothelial cells (ref 91), lymph node stromal cells (ref 92), and nervous cells (ref 93-95). This study focuses on the intestinal epithelium, but, ultimately, an integrated view on the postnatal development of all individual cell types and their interaction during postnatal maturation will be needed to fully understand the complexity of the postnatal transition."

2) My point 2 - The new analyses is insufficient, the authors should include additional plots in the format of Figure 1j (median expression over time or mean+-se expression over time) that will enable explicitly tracking the changes in key enterocyte markers over time - the antimicrobial programs Ccl25, Il18, Lypd8, Nlrp6, Reg3a, Reg3b, Reg3g, the amino acid/peptide transporters Slc7a7, Slc7a8, Slc7a9, Slc15a1, the glucose tranporters Slc2a2, Slc2a5, Slc5a1, the lipid-cholesterol programs Npc1l1, Apoa1, Apoa4, Apob, Dgat1, Dgat2, Cd36 and the purine villus tip programs Slc28a2, Nt5e, Enpp3 and ada. Do any of these increase/decrease at specific time points, how could this be related to changes in diet/exposure to bacteria? As it stands Figure 3c shows the summed expression of dozens of markers and does not facilitate the biological insights that could be gained by examining the temporal profiles of such specific key genes, which have direct physiological function.

As requested, we supplemented aggregated summaries with high resolution temporal plots of key antimicrobial, transporter, and peptide hormone marker genes to better illustrate their

regulation in respect to zonation, segmentation and maturation stage (see **new Fig. 1d, Fig. 3e, Fig. 4h, Fig. S1j, Fig. S3f**).

Two examples (Fig. 1d for age-dependent expression and Fig. 3e for crypt versus villus expression) are shown below:

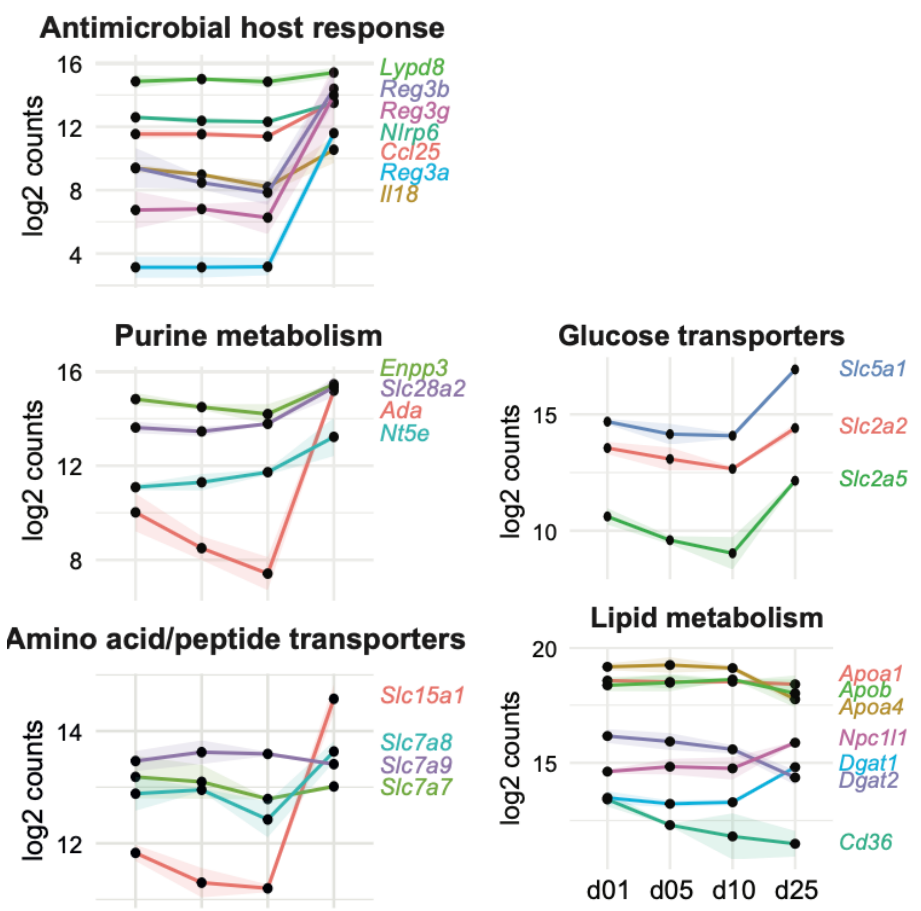

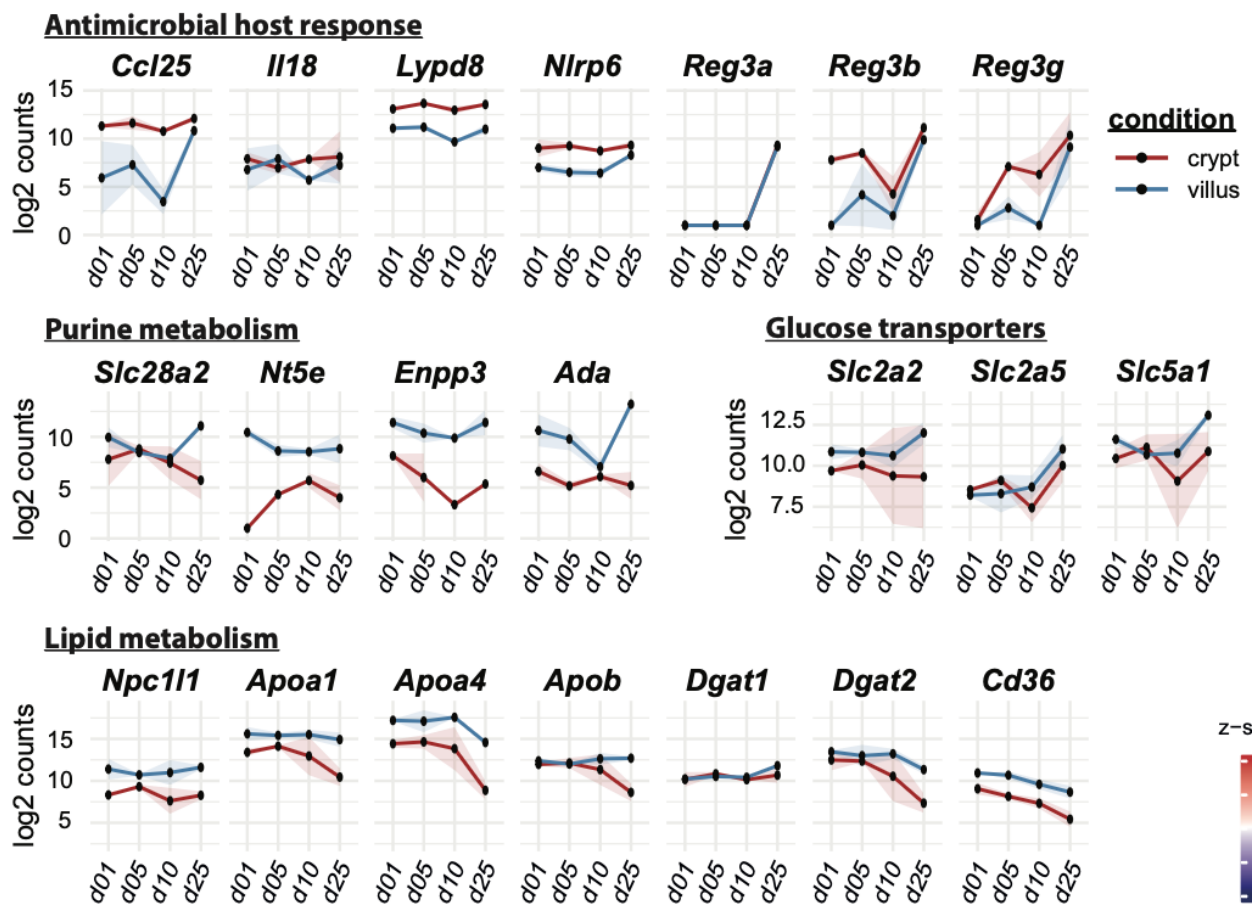

As suggested, former Fig. 3c was moved to the supplement (now Fig. S3b). Temporal changes are discussed in the manuscript text in the respective paragraph as requested (see below). We tried to integrate the description of the additional requested figure panels into the the existing text.

Page 15, line 9-15:
"Whereas expression of Paneth cell-associated antimicrobial genes (e.g. defensins (Defa), phospholipase A2 (Pla2g2a), and lysozyme (Lyz1) and the corresponding GO terms) increased steadily throughout the postnatal period, a marked increase in expression was noted at weaning for more broadly expressed factors of the antimicrobial program including the regenerating islet-derived proteins 3a, b and g (Reg3a, b, and g), interleukin-18 (Il18), the chemokine Ccl25, and the inflammasome component NOD-like receptor family pyrin domain containing 6 (Nlrp6) (Fig. 1c and d, Fig. S1a-b, Fig. S1d, Suppl. Table 1)."

Page 15, line 15-18:
"Similarly, the upregulation of some digestive enzymes (e.g. trehalase (Treh), sucrase isomaltase (Sis)) and of enzymes of the purine metabolism characteristically localised at the villus tip, such as the adenosine deaminase (Ada), was most pronounced at weaning (Fig. 1c and d, Fig. S1a-b, Fig. S1d, Suppl. Table 1)."

Page 15, line 18-25:
"Transmembrane transporters for iron (e.g. Trf and Meltf), folate (e.g. Folr1), vitamin A (e.g. Ttr), bile acids (e.g. Abcb11), glutathione peroxidases (e.g. Gpx3), as well as the multiligand endocytotic receptor Lrp2 were downregulated at weaning, whereas amino acid/peptide transporters such as the di- and tri-peptide transporter solute carrier family 15 member (Slc15a1, also known as peptide transporter, PEPT1) and the neutral amino acid transporter Slc7a8 as well as the glucose transporters Slc2a2, Slc2a5 and Slc5a1 showed a marked increase in expression at weaning, likely corresponding to a change in dietary availability and/or metabolic demand at this critical time period (Fig. 1c and d, Fig. S1a-b, Suppl. Table 1) refs 26,54-56."

Page 15, line 25-page 16, line 4:
"Interestingly, whereas expression of the final enzyme in the triacylglycerol (TAG) synthesis diacylglycerol-O-acyltransferase 2 (Dgat2), the fatty acid translocase Cd36, and the apolipoprotein A-IV (Apoa4) decreased during the postnatal period, an increase in the expression of the non-essential TAG synthesis enzyme Dgat1 and the cholesterol transporter Niemann-Pick c1-like 1 (Npc1l1) was observed at weaning (Fig. 1d)."

Page 17, line 16-18:
"Similarly, no major differences were observed during the early postnatal phase between GF and SPF mice for selected genes with known important epithelial function (Fig. S1j and Fig 1d)."

Page 21, line 20-26:
Similarly, many of the genes associated with the antimicrobial host response program were more dominantly expressed in the crypt/intervillus epithelium at all ages, consistent with the protection of the regenerative stem cell niche against microbial challenge, whereas most transport molecules for dietary substrates such as glucose, lipids, or purine metabolites were higher expressed by the villus epithelium, in closer contact to the luminal content (Fig. 3e). No major zonal expression was found for amino acid/peptide transporters, except for Slc15a1, whose major upregulation previously observed at weaning happened dominantly in the villi (Fig. S3f).

Page 25, line 10-15:
Similarly, amino acid/peptide transporter and antimicrobial program gene expression exhibited little segmental differences, whereas the adenosine desaminase (Ada) showed increased expression in the proximal small intestine and the glucose transporters Slc2a2 and Slc2a5 and fatty acid translocase Cd36 in the middle and proximal intestine at all ages, consistent with the known anatomical site of the maximal absorption of their respective substrate (Fig. 4h).

3) Dynamics of key EEC hormones - Thank you for adding this, please also show as plots of median vs. time or mean+-SE vs time, Figure 3g makes it hard to follow the trends. Please discuss the trends and their potential physiological significance, e.g. it seems that Gip, associated with satiety and insulin secretion strongly increases in the villi enterocytes only at day 25 whereas Sct and Cck seem to decrease from day 1 to day 25? Please quantify changes (you can also plot a heatmap of the hormone median levels vs. developmental time sorted from early to late). Some discussions on the trends would greatly add to the manuscript. Are these related to pancreas maturations, changes in diet, weaning etc?

Additional plots (like the plots added in response to point 2) showing the median expression of the individual EEC peptide hormone-encoding genes over time were included illustrating zonal (**new Fig. 3g and new Fig. S3h**) and segmental (**new Fig. 4k**) differences during the postnatal period.

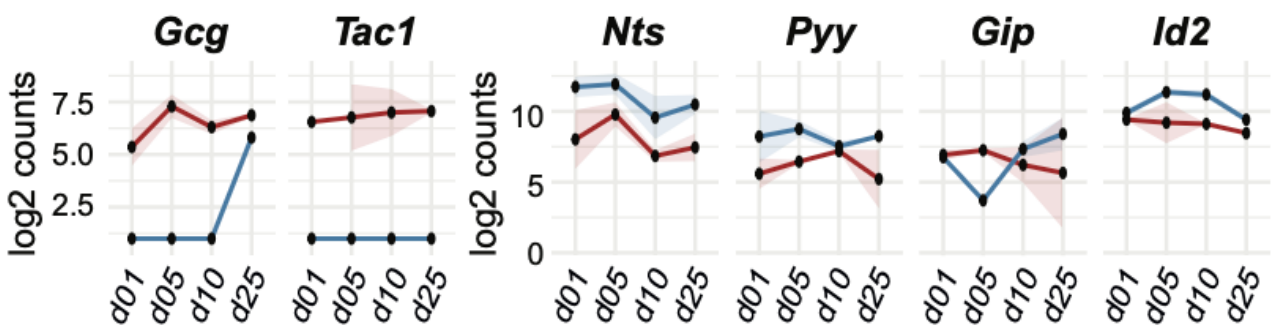

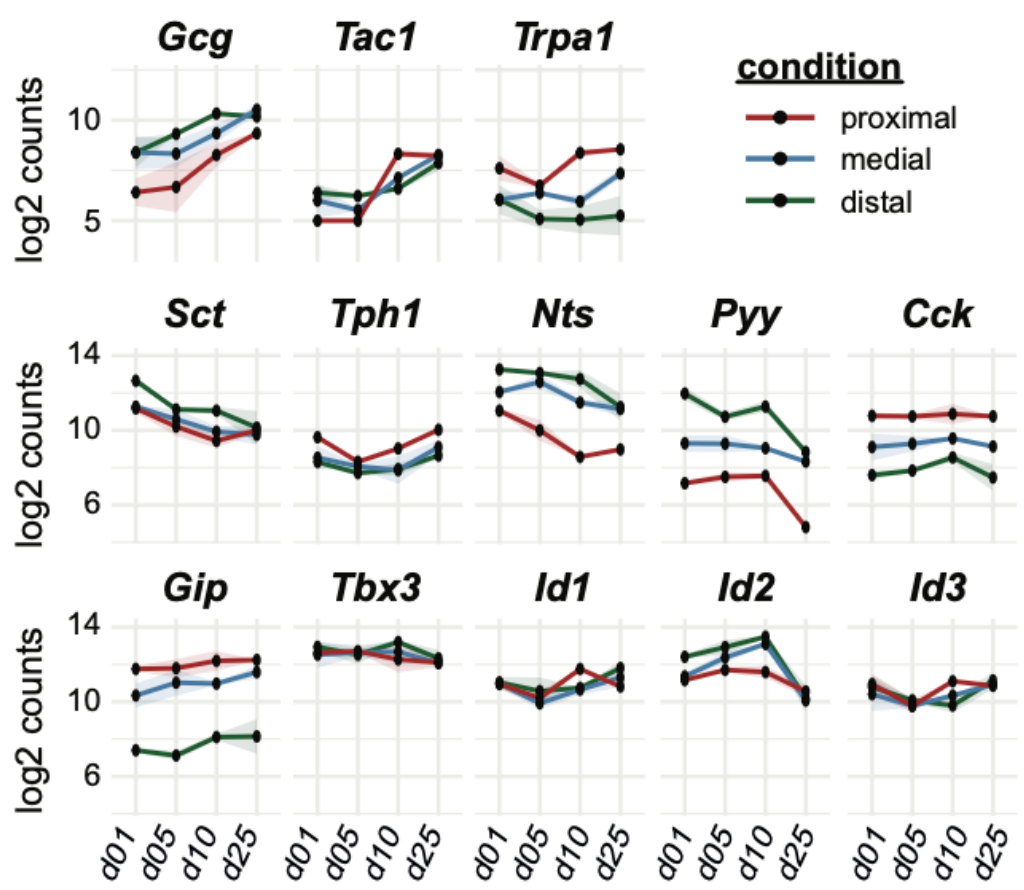

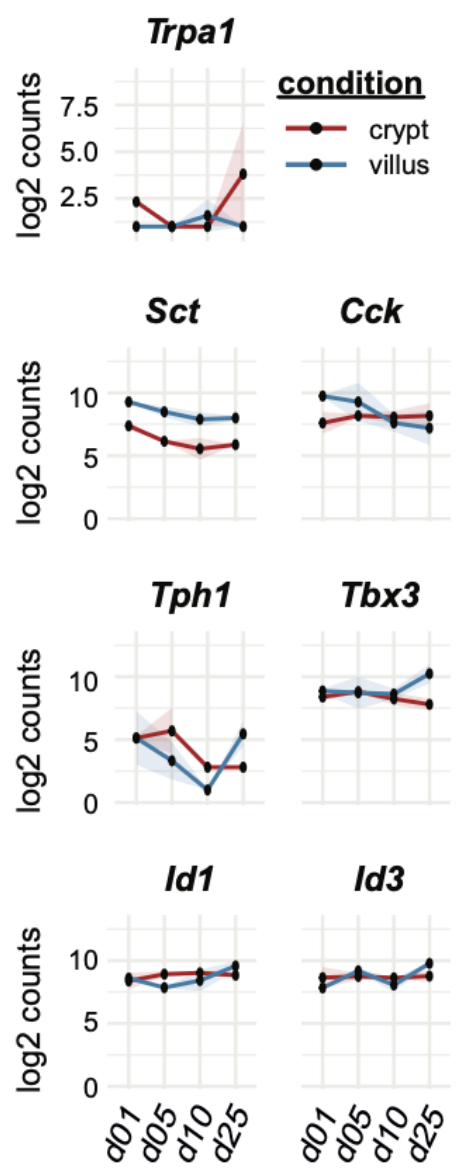

Temporal and zonal expression changes are discussed in the manuscript text (page 22, line 5-10):

„Crypt-dominated gene expression was found for glucagon (*Gcg*) and tachykinin (*Tac1*), whereas a more pronounced expression was found at the villus level for bile and pancreas-secretion inducing secretin (*Sct*) and cholecystokinin (*Cck*), neurotensin (*Nts*), neuropeptide YY (*Pyy*), and transcriptional regulator inhibitor of DNA binding (*Id2*) throughout or at specific time points during postnatal development (Fig. 3g, Fig. S3g-h).“

Also, we added a sentence specifically on villus EEC peptide hormone expression prone to facilitate luminal sensing (page 22, line 10-15):

“Interestingly, villus epithelial gene expression facilitating luminal sensing increased during the postnatal period for the satiety- and insulin-inducing but gastric acid-suppressing glucagon (Gcg) and gastric inhibitory peptide (Gip), whereas it decreased for the bile, gastric acid and pancreas secretion-inducing peptide hormones secretin (Sct) and cholecystokinin (Cck). This might reflect changes in the global energy metabolism after birth and the early start of hepatic bile acid synthesis and may support efficient absorption of breast milk nutrients [70,71].”

Spatial changes are discussed on page 25, line 18-22:

„Segmental differences of EEC hormone-encoding genes were noted, with a decreased expression of cholecystokinin (*Cck*), and the incretin gastric inhibitory peptide (*Gip*) transcripts and an increased expression of glucagon (*Gcg*), neurotensin (*Nts*), and the peptide YY (*Pyy*) transcript along the proximal-to-distal length of the intestine at all ages (Fig. 4j-k).“

For future revisions please paste all relevant responses (new text + figures) under the respective points in the response to reviewer comment file, this would greatly assist in the review process. E.g., when a reviewer faces a sentence such as: "Moreover, cell type marker genes including marker genes for EECs were included in Fig. 1-4 (new Fig.1i, new Fig. 2g, new Fig. 3g, new Fig. 4j)." it is very difficult to track the changes made. Please help reviewers by making the response file more accessible.

As requested, we pasted any new figure panels (or representative examples thereof) or text changes below our response in the point-by-point response letter.

Reviewer #2:
The authors have addressed many of my previous concerns and, overall, the data are sufficient for publication. However, the authors should address the following points, including a reconsideration of the interpretation of the two aspects of the Results.

#1
Fig.S3 was not successfully uploaded. Fig. S2 appears to have been uploaded in place of Fig. S3. Please upload Fig.S3 correctly.

We apologise that we had uploaded the wrong eps file for Fig. S3 (eps file of Fig. S2 instead) and have corrected this mistake.

#2
In the Results section (page 15, line 15; page 21, line 14), the authors suggest that downregulation of Lrp2 after weaning, or decreased expression of genes represented by GO terms related to lipid metabolism, may be linked to the high metabolic demand imposed by the lipid-enriched milk diet of the newborn. This interpretation appears controversial in light of the presented data.
Specifically, the upregulation of the fatty acid metabolic process GO term after weaning (Fig. 1h, 3i, 4h, 5e) suggests that intestinal cells are metabolically adapted to lipid-containing food after weaning. Based on current understanding, fatty acid metabolic process represents the active execution of fatty acid metabolism, whereas regulation of lipid metabolic process represents a preparatory or modulatory layer that adjusts lipid metabolic capacity in response to environmental or developmental cues.
From this perspective, the data indicate that during the preweaning stage, the intestine may regulate or sense lipid metabolic state, but does not necessarily execute high levels of lipid metabolism. In contrast, after weaning, intestinal epithelial cells appear poised to actively metabolize lipids.
Accordingly, the observed shift from enrichment of regulation of lipid metabolic process in crypt cells of the prenatal intestine (Fig. 3h) to enrichment of fatty acid metabolic process in bulk cells (Fig. 1h, 4g,h), villus cells (Fig. 3i), and enterocytes (Fig. 5e) likely reflects a developmental transition from a preparatory metabolic state to active lipid metabolism. The enrichment of regulation of lipid metabolic process in immature intestinal epithelial cells (Fig. 3h) may therefore represent a primed state in which cells respond to environmental cues and become poised for subsequent high lipid metabolic demand prior to full activation of lipid metabolic pathways (Fig. 1h, 3i).
In this context, exposure to milk during the preweaning stage could promote intestinal functional maturation and contribute to the emergence of more mature enterocytes. I therefore suggest that the authors reconsider and revise the discussion of these findings in the Results section to better align with the presented data.

We agree with the reviewer's interpretation of our findings. We have therefore revised and adjusted the description of our findings as suggested.

Page 15, line 18-25 (page 15, line 18-25 in the revised manuscript) was changed to:
"Transmembrane transporters … as well as the multiligand endocytotic receptor *Lrp2*, were downregulated at weaning, whereas amino acid/peptide transporters such as the di- and tri-peptide transporter solute carrier family 15 member (*Slc15a1*, also known as peptide transporter, PEPT1) and the neutral amino acid transporter *Slc7a8* as well as the glucose

transporters *Slc2a2*, *Slc2a5* and *Slc5a1* showed a marked increase in expression at weaning, likely corresponding to the change in dietary availability and/or metabolic demand at this critical time period (Fig. 1c and d, Fig. S1a-b, Suppl. Table 1) [26,54-56]."

Page 17, line 21-25 commenting on Fig. 1h was changed to: "Gene ontology (GO) analysis mainly assigned the NNU time pattern to metabolic and catabolic processes (e.g. "fatty acid metabolic process" GO:0006631, or "small molecule catabolic process" GO:0044282), suggesting increased active lipid and monosaccharide metabolism after weaning, and the NND time pattern to the loss of foetal-type enterocytes at weaning (e.g. "vacuolar acidification" GO:0007035, "lysosome organization" GO:0007040) (Fig. 1h, Suppl. Table 2)."

Page 22, line 21-26 in the revised version) was changed to: "Also, the crypt/intervillus epithelium exhibited decreased expression of genes represented by the GO terms "regulation of lipid metabolic process" (GO:0019216) and "cellular lipid catabolic process" (GO:0016042) and a simultaneous increase of genes represented by the term "fatty acid metabolic process" (GO:0006631) at weaning, likely representing a shift from a "primed/regulatory" state to an "active execution" of lipid metabolism (Fig. 3c, h and i, Fig. S3c and d, Suppl. Table 9) [72]."

Page 23, line 10-11 commenting on Fig. 3i was changed to: "… whereas "fatty acid metabolic process" (GO:0006631) was upregulated representing the start of active lipid metabolism (Fig. 3i, Suppl. Table 9)."

Page 25, line 3-8 commenting on Fig. 4f-g was changed to: "Cluster 2, characterised by GO terms associated with "disruption of plasma integrity"/"disruption of cellular anatomical structure in another organism" (GO:0051673 and GO:0140975), "mucosal immune response" (GO:0002385), and "fatty acid metabolic process" (GO:0006631) most likely representing Paneth cell-derived antimicrobial molecules and the start of epithelial lipid metabolism, was comprised of genes uniformly upregulated in all segments post-weaning (Fig. 4f-g, Fig. S4c, Fig. S4f)."

Page 27, line 1-6 commenting on Fig. 5e was modified to "GO term analysis of the age-dependent transcriptional profile of absorptive enterocytes confirmed the previously observed developmental changes of metabolic processes illustrated by the upregulation of genes represented by the GO term "fatty acid metabolic process" (GO:0006631), suggesting increased active lipid metabolism and identified a downregulation of translational activity with weaning (Fig. 5e, Suppl. Table 14)."

#3
Goblet and Paneth cells are not known to possess absorptive functions, although they can sense nutrients. Therefore, the statement that GO term analysis of differentially expressed genes revealed increased absorption with increasing age (Fig. 6c; Supplementary Table 14; page 26, line 10) may lead to misunderstanding.
The observed GO term enrichment may instead reflect increased bioenergetic demand and potential nutrient-handling capacity required to support enhanced secretory and antimicrobial functions in these cell types. Alternatively, possible contributions from ambient enterocyte RNA or cell doublets should be considered.
I recommend revising the authors' interpretation.

As recommended, we revised our interpretation of the GO term change of Fig. 6c in the manuscript text.
Page 27, line 26-page 28, line 3 was modified to: "GO term analysis of DE genes revealed reduced translational activity (GO:0002181) and increased intestinal absorption (GO: 0050892)

with increasing age, likely reflecting the bioenergetic demand rather than genuine absorptive function (Fig. 6c, Suppl. Table 14)."

Reviewer #3:
The authors have addressed my comments fully. Looking forward to seeing this work published as it's an informative study for the field.

We thank the reviewer for his/her positive judgment.

Reviewer #4
The revised manuscript does not address my previous concerns regarding the suboptimal quality and reproducibility of the scRNA-seq dataset. I also disagree with the revised quality control criteria, particularly the exclusion of the proportion of mitochondrial genes.

More specifically,
1. The authors did not attempt to optimize their single-cell isolation protocols, which appear to be biased toward capturing viable preweaning epithelia cells and are suboptimal for isolating viable adult intestinal epithelial cells. Given the depletion of intestinal stem cells and TA cells at day 10 and 25, together with the overall suboptimal quality of these samples, comparative analyses across developmental time points are severely limited. As a result, the single-cell dataset has limited value as a resource. Furthermore, the newly added differentiation trajectory analysis of enterocytes appears to reflect spatial zonation along the crypt-villus axis rather than true differentiation trajectories of enterocytes.

To demonstrate the overall reproducibility and robustness of our data and value as a resource, we integrated our day 1, 5, 10 and 25 datasets with the only published scRNA-Seq dataset of day 10 and day 24 intestinal epithelial cells (Matsuki et al., Muc Immunol., 2026) using Seurat. We then analysed the absorptive enterocyte cluster, which is the most abundant epithelial cell type of our dataset. In this cluster, cells were grouped into crypt, lower villus zones (V1–V3) and upper villus zones (V4–V6), according to gene expression from Moor et al. (Moor et al., Cell, 2018). Genes significantly differentially expressed between zones in at least one sample (adjusted $p<0.05$) were retained. A scatter plot was generated using log2FC values, which were further log10 compressed for better visualisation, from our first day 25 sample on the x-axis and the corresponding values from our second day 25 samples on the y-axis. Similarly, a second scatter plot comparing the day 24 sample of Matsuki et al. on the x-axis and the corresponding values from our day 25 samples on the y-axis was also created. To quantify agreement between samples, a normalised product ratio concordance score was computed for each gene as follows: $C = (2*a*b) / (a^2+b^2)$ where a and b denote the log2FC values in the two compared samples. This metric ranges from -1 (equal-magnitude regulation in opposite directions) to +1 (identical regulation). Concordance distributions were visualized as histograms using a bin width of 0.05, and median concordance values are labelled. The code used for this analysis can be found at https://github.com/jschoeneich/epithelial_cell_diversity/tree/main/robustness.

The comparative analysis of our two day 25 scRNA Seq datasets (d25_1j and d25_2j) demonstrates the reproducibility of our approach. The UMAP revealed an almost identical cell distribution of the villus cell zones. The replicate consistency of the two samples correlating genes differentially expressed between the upper and lower villus revealed a high median concordance of 0.96, suggesting a high reproducibility of our approach.

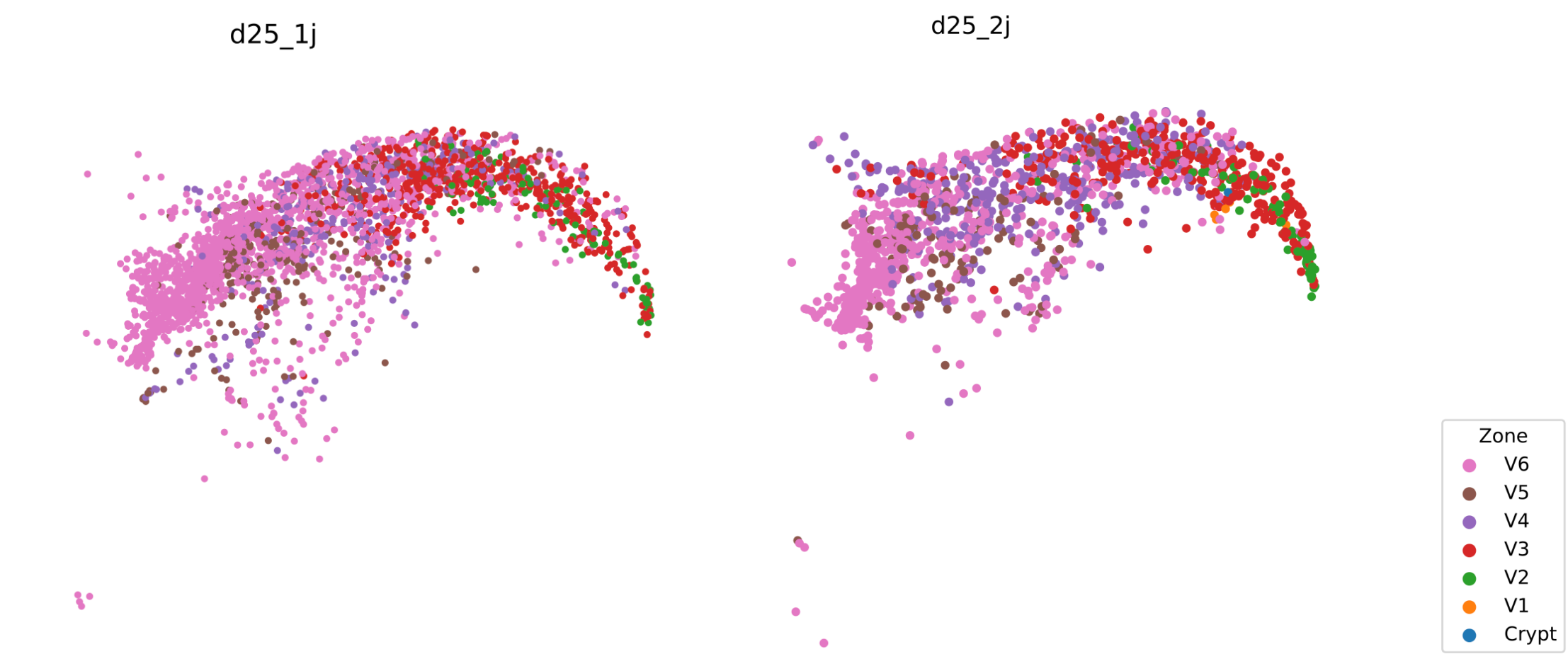

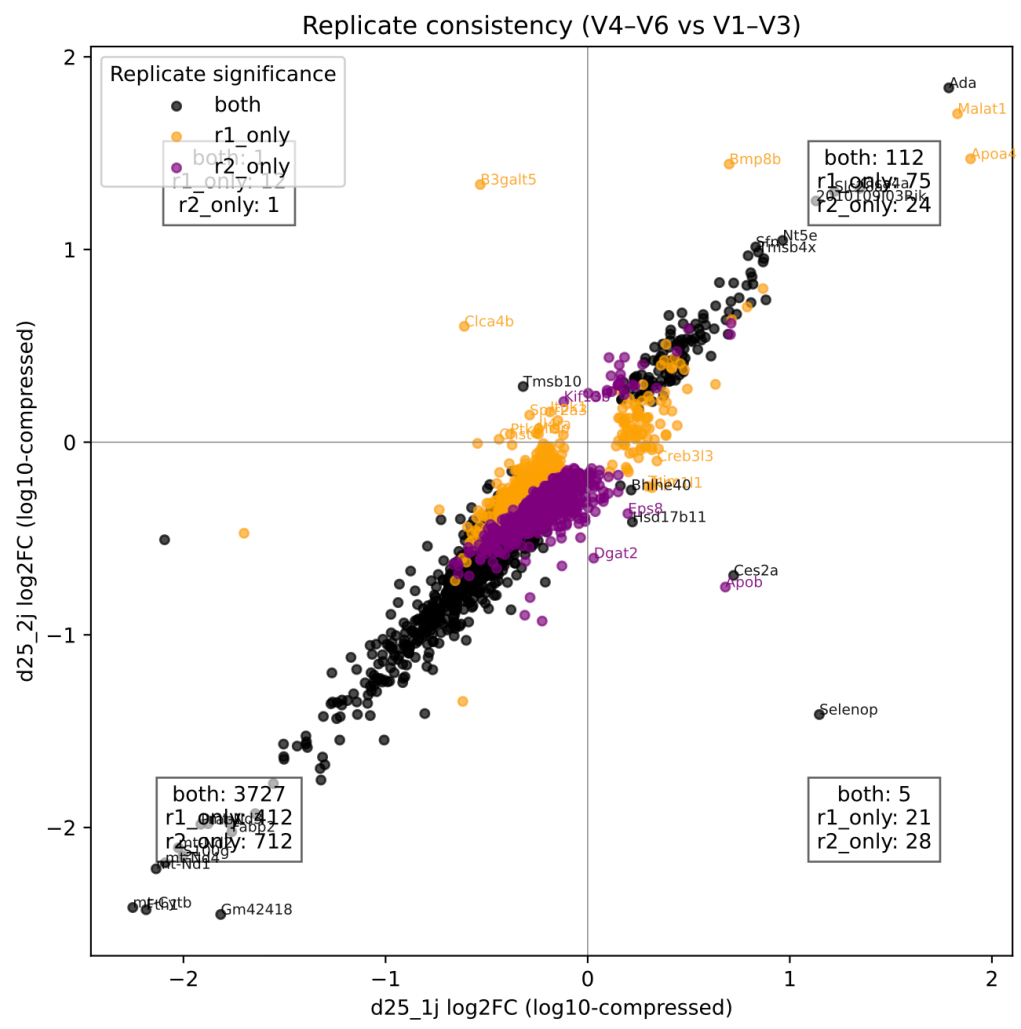

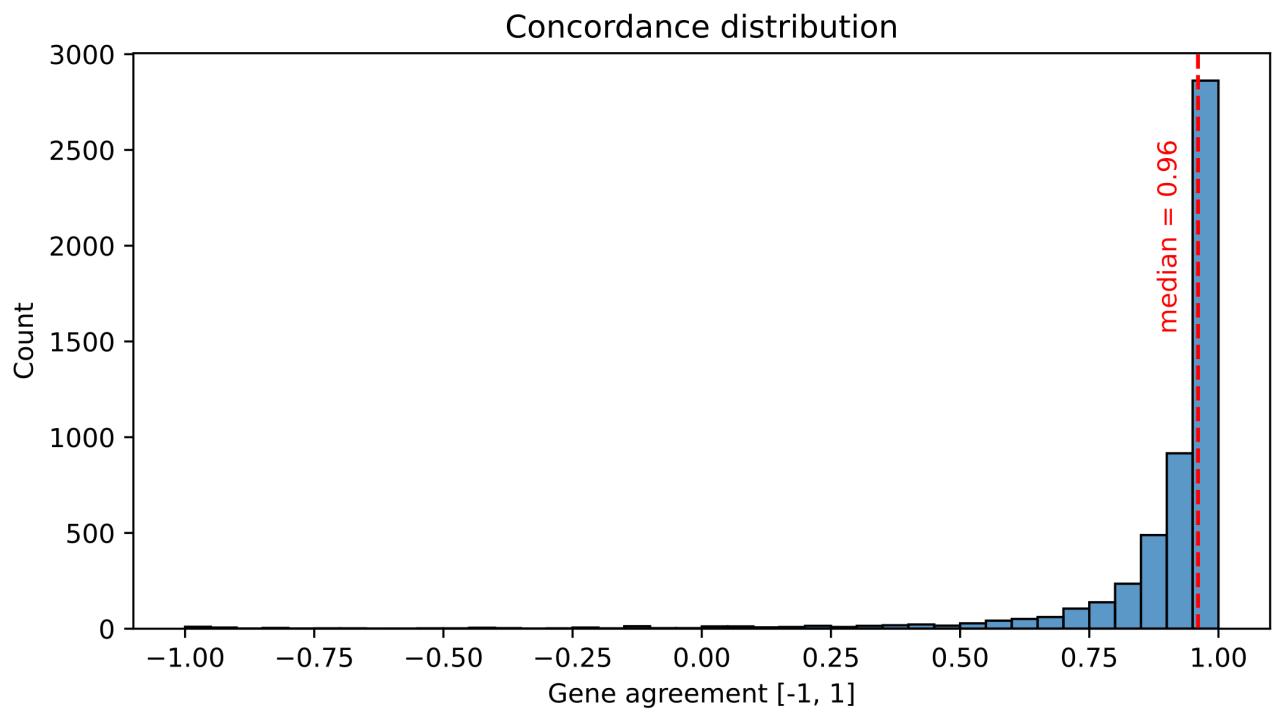

The comparative analysis of our day 1, 5, 10, and 25 scRNA-Seq datasets (d01j, d05j, d10j and d25_1j and d25_2j) and the Matsuki et al. study dataset of day 10 (d10m) and day 24 (d24m) demonstrated the value of our approach. The UMAP of our datasets revealed the representation of all villus cell zones (with the exception of an underrepresentation of the lowest villus and crypt zone in our day 25 samples). The UMAP of the Matsuki datasets revealed an almost exclusive crypt zone phenotype in the day 10 dataset and a crypt-dominated day 24 dataset. The replicate consistency of the day 25 sample from us (j(d25)) and from the Matsuki dataset (d24m) correlating genes differentially expressed between the upper and lower villus revealed a high concordance distribution of 0.88.

Our study (j) absorptive enterocyte cluster of day 1 (d01), day 5 (d05) and day 10 (d10)-old mice.

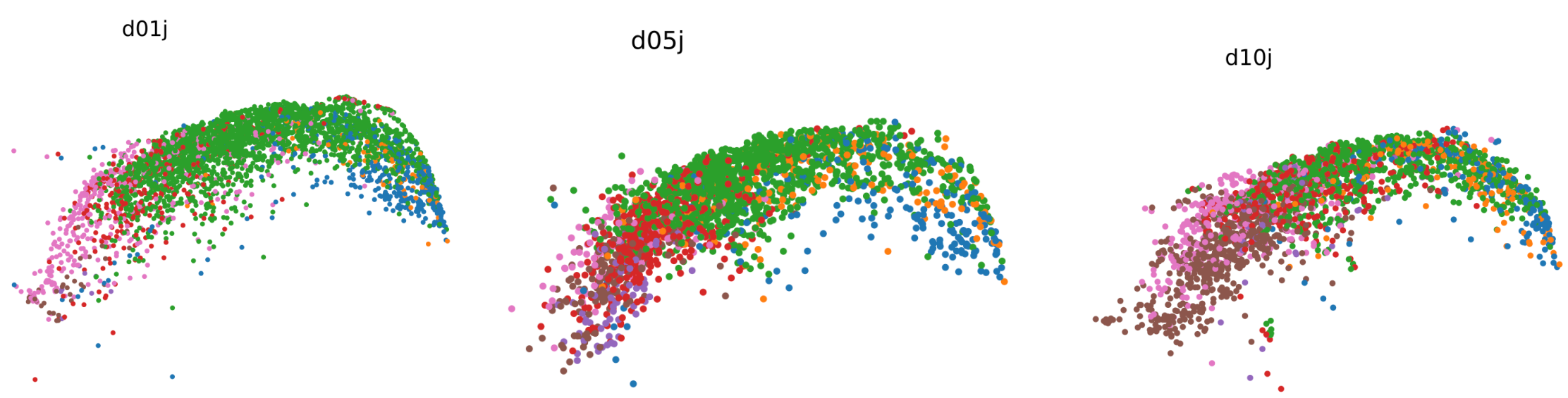

Our study (j) absorptive enterocyte cluster of the two (_1 and _2) samples of day 25 (d25)-old mice.

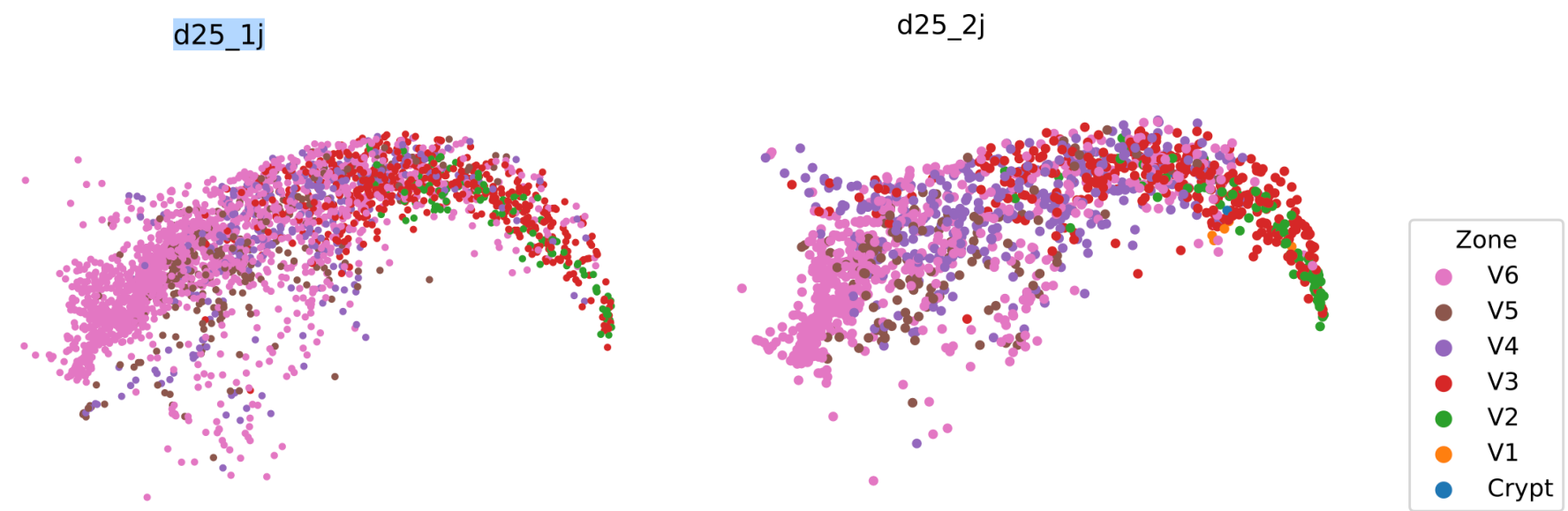

Matsuki et al., 2026 study (m), absorptive enterocyte cluster of day 10 (d10m) and day 24 (d24m)-old mice.

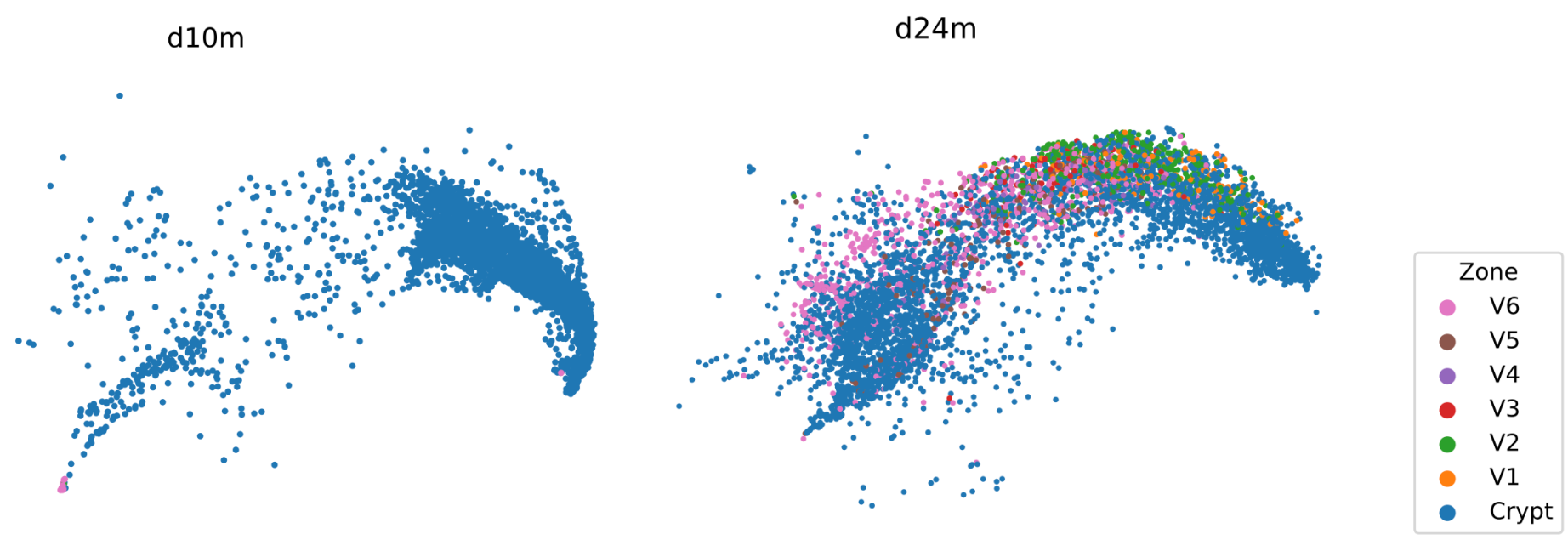

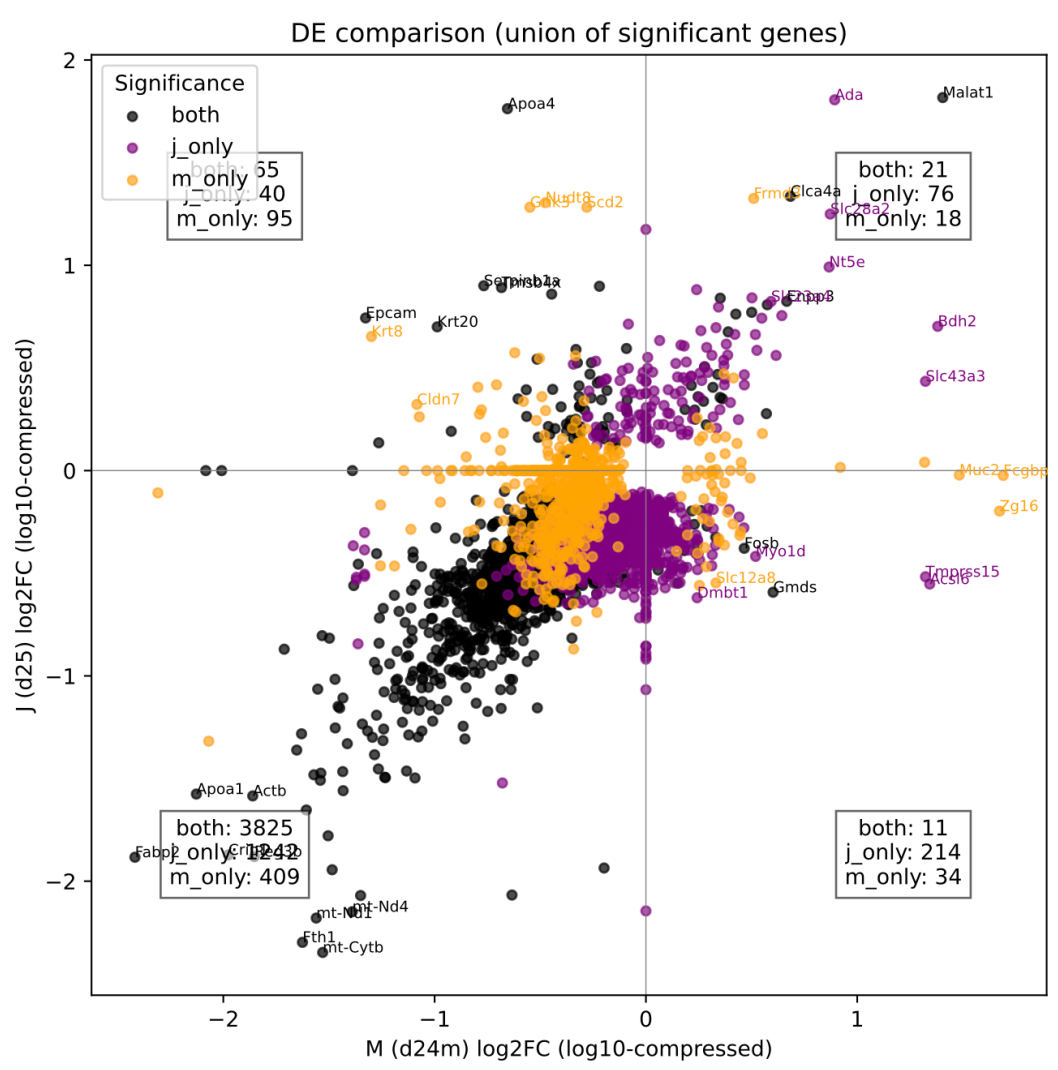

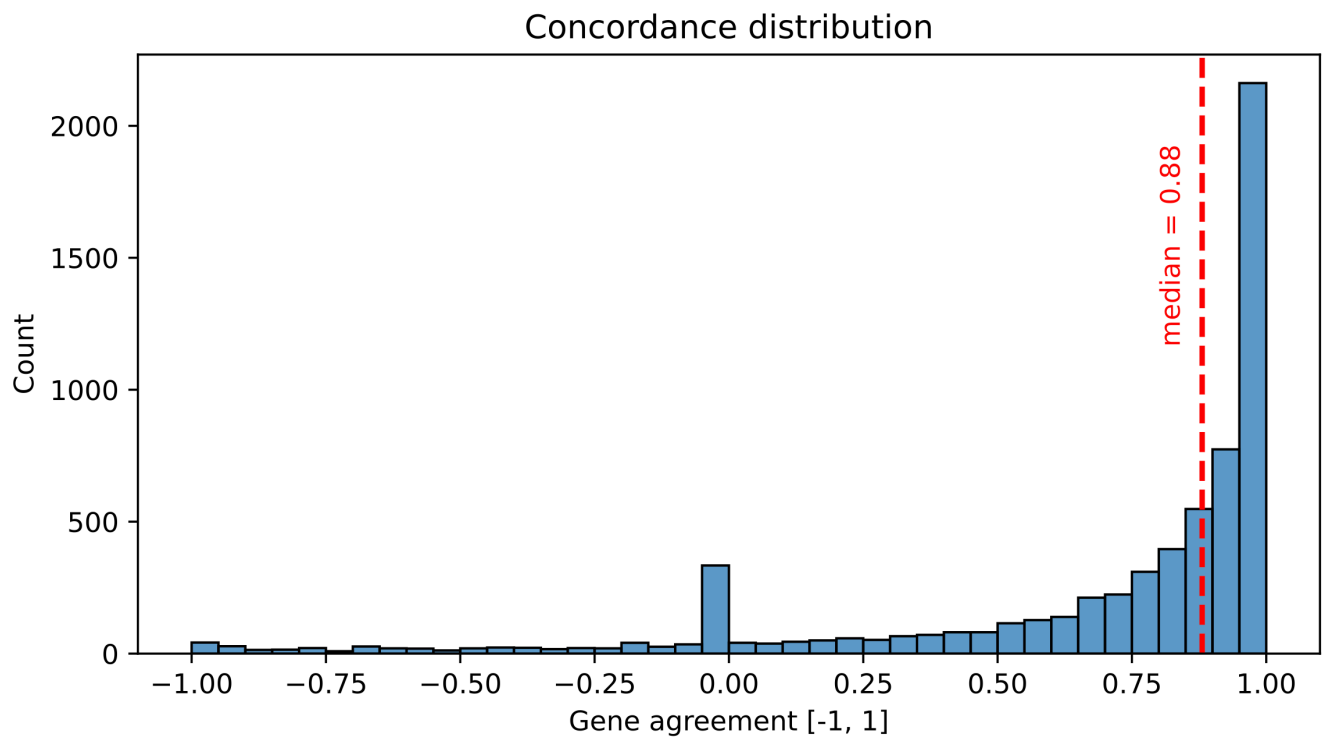

The zone composition (total cells per zone, absolute cell counts) further illustrated the representation of the crypt and all villus zones on our day 1, 5, 10 datasets and the representation of most villus zones in our two day 25 datasets. Notably, the day 10 dataset from the Matsuki et al. study almost exclusively contained crypt cells and also the day 24 datasets of the Matsuki et al., study contained an underrepresentation of villus cells. These results suggest that a strong selection for viability may have led to an underrepresentation of upper villus epithelial cells. As described in the discussion section, our study focused on the neonatal intestine with intact villi but prior to the development of deep crypt invaginations.

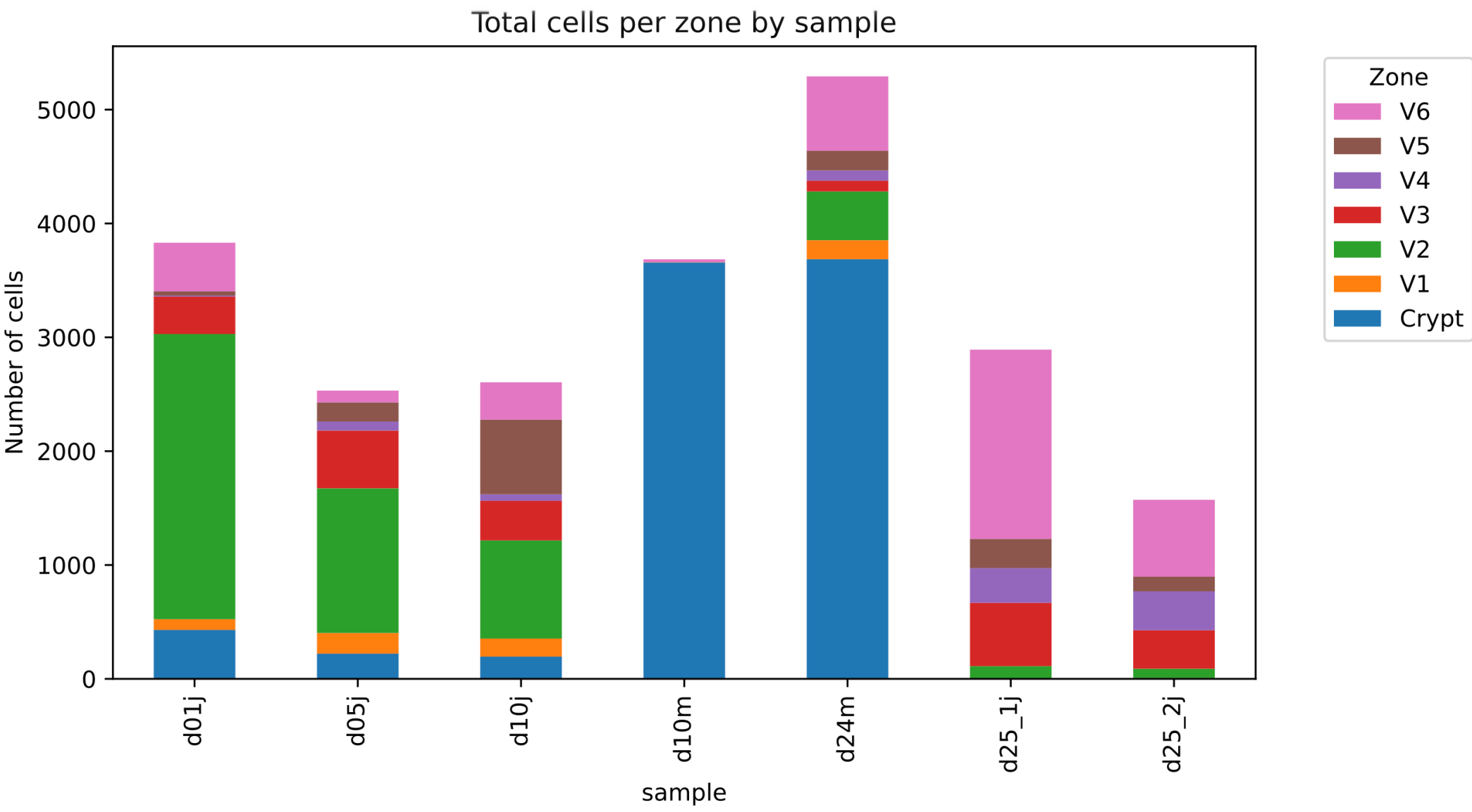

Finally, while we agree with the reviewer that our day 25 scRNA-Seq dataset is of suboptimal quality, we would like to stress that our day 25 scRNA-Seq dataset represents only a minor part of our study, that also presents bulk transcriptional (Fig. 1), and proteomic (Fig. 2) analysis of the maturation and differences caused by zonation (Fig. 3), segmentation (Fig. 4), microbiota (Suppl. Fig. 1g-j) and *Salmonella* infection combined with bulk RNA-Seq (Fig. 7) on the intestinal epithelium during the postnatal period. Since the zonation along the crypt-villus axis reflects the migration of the epithelial cells along the crypt-villus axis, which is accompanied by their differentiation and maturation (Kolev et al., 2023), we believe that the trajectory identified in our study reflects the differentiation of intestinal epithelial cells *in situ*. We show the filtering and quality assessment results of our scRNA-Seq analysis in Suppl. Fig. 5a-e and discuss this as a limitation of our study in the discussion section (page 30, line 21-page 31, line 3: "Nonetheless, the isolation and analysis of primary small intestinal epithelial cells remain technically challenging. Firstly, detachment from the basal membrane, a prerequisite for the isolation and analysis of epithelial cells, reduces their fitness and together with the proapoptotic signals during infection-induced inflammation may reduce their viability [91]. Secondly, the anatomical organisation of the intestinal epithelium makes it difficult to isolate representative fractions of viable cells simultaneously from villi and crypts at different ages using the same protocol [92]. As our analysis focused on the early postnatal period, we prioritised villus cell over adult crypt cell coverage, which explains the underrepresentation of crypt-based stem, TA, and Paneth cells in our adult sample. However, the crypt epithelium of adult mice has been previously characterised [2,3].").

2. Given the suboptimal quality of their single-cell data, it is critical to demonstrate the robustness and reproducibility of the conclusions drawn from these data. Although the authors state that the two day-25 replicates are highly similar, no supporting evidence is provided to substantiate this claim.

As requested, we here show the results of the comparative analysis of our two day 25 samples and an integrated analysis of our day 1, 5, 10 and 25 samples with the day 10 and 24 datasets of the Matsuki et al. study in the response to point 1, reviewer #4 demonstrating the reproducibility of our approach.

3. As also noted by Reviewer 3, intestinal stem cells and enterocytes are metabolically active

and therefore express mitochondrial genes at relatively high levels to support nutrient transport. However, an excessively high proportion of mitochondrial genes (>20%) typically indicates cytoplasmic leakage during tissue dissociation. In Figure S5d, the majority of cells from day 25, which already exhibit suboptimal quality, display extremely high proportions of mitochondrial genes (>50%), strongly suggesting that these cells are damaged rather than biologically intact.

We completely agree with the reviewer's comment on the high mitochondrial gene count in our day 25 scRNA-Seq sample indicating a suboptimal quality and discuss this in the limitations of the study paragraph (page 30, line 21-page 31, line 3:
"Nonetheless, the isolation and analysis of primary small intestinal epithelial cells remain technically challenging. Firstly, detachment from the basal membrane, a prerequisite for the isolation and analysis of epithelial cells, reduces their fitness and together with the proapoptotic signals during infection-induced inflammation may reduce their viability [91]. Secondly, the anatomical organisation of the intestinal epithelium makes it difficult to isolate representative fractions of viable cells simultaneously from villi and crypts at different ages using the same protocol [92]. As our analysis focused on the early postnatal period, we prioritised villus cell over adult crypt cell coverage, which explains the underrepresentation of crypt-based stem, TA, and Paneth cells in our adult sample. However, the crypt epithelium of adult mice has been previously characterised [2,3]."). However, reviewer #3 (point 7) during the last revision had stated that "… With respect to enterocytes and intestinal stem cells, these two cell subsets by scRNA-seq and other methods have been well characterized to generate more mitochondria due to their metabolic activity. As such, it is generally not recommended filter IECs based on mitochondrial gene expression…". In response to this statement, we had removed the general mitochondrial gene count filter and replaced it by the exclusion of two clusters segregating due to high mitochondrial gene counts (cluster 2 and 11, as illustrated in Suppl.- Fig. 5a-e). Also, as illustrated by the comparative analysis of our and the dataset published by Matsuki et al., the rigid removal of cells with lower viability may also remove cells of the upper villus zones that may still be of biological significance.
