# Supplementary material for: Developmental factors drive the compartmentalized and discontinuous maturation of the small intestinal epithelium during the early postnatal period
Source: PLoS Biol. 2026 Aug 3;24(8):e3003212. doi: 10.1371/journal.pbio.3003212 (PMC13460745; doi:10.1371/journal.pbio.3003212)
Supplement: Supplementary file 3 [file pbio.3003212.s031.pdf]

Point-by-point response

<u>Data and policy related issues</u>

Many thanks for providing the data underlying the graphs shown in the figures. We do require all individual quantitative observations, thus please add or let us know where to find the data shown in the following figures: Fig. 1B, D, E, F, I; Fig. 2B, C, G, H; Fig. 3B, E, F, G; Fig. 4B, C, D; Fig. 5B, F, G, H, I, M, N; Fig. 6B, D-F, I, J; Fig. 7A, E,F; Fig. S1E, F, G, J; Fig. S2C-E; Fig. S3B, F-M; Fig. S4B; Fig. S5, Fig. S6B and Fig. S7.

We have added the underlying data for Fig. 1b, d, e, f and i (S1 Data), Fig. 2b (S5 Data), Fig. 2c and g (S4 Data), Fig. 2h (S6 Data), Fig. 3b, e, f and g (S9 Data), Fig. 4b (S10 Data), Fig. 4d (S11 Data), Fig. 5b (S12 Data), Fig. 5f and g (S15 Data), Fig. 6b (S14 Data), Fig. 6d (S15 Data), Fig. 6j (S12 Data), Fig. 7a and f (S18 Data), Fig. S1e, f and j (S1 Data), Fig. S1g (S3 Data), Fig. S2c, d and e (S4 Data), Fig. 3f, g, h, j, k, l and m (S9 Data), Fig. S3i (S6 Data), Fig. S4b (S11 Data), Fig. S5g, h and i (S13 Data), Fig. S5k and l (S15 Data), Fig. S6b (S15 Data) and Fig. S7a, b, c, d and e (S18 Data). Unfortunately, underlying data for Fig. 5h, Fig. 5m and Fig. 6f (pseudotime overlaid on UMAPs) and Fig. 6e, Fig. 6i, Fig. 7e (cell and expression overlaid on UMAPs), Fig. 4c, Fig. 5i, Fig. 5n and Fig. S3b (z-score of the mean) could not be provided as we did not find a meaningful way to present such datasets as a table. We would refer the readers to the deposited raw data. Information about each GEO/ProteomeXchange dataset is available at the end of each figure legend as well as in the "data sharing plan" chapter.

1) Please also ensure that the data files referred to (in the manuscript, figure legends, and the Description field when uploading your files) using the following format verbatim: S1 Data, S2 Data, etc. Please also save the files using exactly the following convention: S1_Data.xlsx (using an underscore).

All "Suppl. Table X" have been replaced by "SX Data" throughout the manuscript and files have been saved with the suggested convention.

2) Please also ensure that figure legends in your manuscript include information on WHERE THE UNDERLYING DATA CAN BE FOUND, and ensure your supplemental data file/s has a legend. And that your Data Statement in the submission system accurately describes where your data can be found.

All figure legends include information on where the underlying data can be found, and all supplemental data files have a legend at the end of this manuscript. In addition, all individual underlying datasheet have an explanatory title and a link to the figure they complement.

3) Please note that I can't open the file for Fig. S5, so please upload it again and make sure you include the data underlying all the graphs included in this figure.

We apologize for that. We have reuploaded Fig. S5 in the system. In addition, we have reuploaded Fig. S6 as we realized that panel c was still referring to the different EEC clusters using the number, instead of the name of the EEC subtype as in the panel d (panel c is the underlying data of panel d).

4) Please make the data deposited in the ProteomeXchange (PXD059639) publicly available at this stage.

We have requested ProteomeXchange to make our data publicly available and we were requested to provide a doi prior to this. So, we suggest that we make the proteome data publicly available as soon as you provide us with a doi of the manuscript. Thank you for your understanding.

<u>Journal Requirements:</u>

1) Thank you for stating "Experimental animal work was approved by the North Rhine-Westphalia Office of Nature, Environment and Consumer Protection (LAVE, approvals 81-02.04.2017.A397)."

Please insert an Ethics Statement at the beginning of your Methods section, under a subheading 'Ethics Statement' and include the details
NOTE: It must include:
i) The name(s) of the Institutional Review Board(s) or Ethics Committee(s)
ii) The approval number(s), or a statement that approval was granted by the named board(s)

We have inserted an Ethics Statement chapter at the beginning of our Methods section as requested.

2) Please ensure that the funders and grant numbers match between the Financial Disclosure field and the Funding Information tab in your submission form. Note that the funders must be provided in the same order in both places as well.
Currently, " ZH 1254/2-1 to Z.K.," is missing from the Funding Information

We have checked and the funding information on ZH 1254/2-1 to Z.K. is included both in the funding sources section of the manuscript text and in the online system of PLoS Biology.

3) We are unable to open your Supporting Information file [Supplemental Figure 5]. Please kindly revise as necessary and re-upload.

We are sorry for that. We have re-uploaded the file of Supplemental figure 5.

4) Please refer to the following article for PLOS Biology's best-practice recommendations for data presentation: https://eur02.safelinks.protection.outlook.com/?url=https%3A%2F%2Fjournals.plos.org%2Fplosbiology%2Farticle%3Fid%3D10.1371%2Fjournal.pbio.1002128&data=05%7C02%7C%7Ce95631ad7ca14112b37b08ded017703b%7C5a6d5ee56edf4a26ba93f5872dbb9614%7C0%7C0%7C639176996252756849%7CUnknown%7CTWFpbGZsb3d8eyJFbXB0eU1hcGkiOnRydWUsIlYiOiIwLjAuMDAwMCIsIlAiOiJXaW4zMiIsIkFOIjoiTWFpbCIsIldUIjoyfQ%3D%3D%7C0%7C%7C%7C&sdata=Wp7K7zYCFj4jBfBpM%2FfOubI2ffC8Gd7pTOn7DSoinHk%3D&reserved=0.
Guidelines for statistical reporting, if relevant, may be found here: https://eur02.safelinks.protection.outlook.com/?url=https%3A%2F%2Fjournals.plos.org%2Fplosbiology%2Fs%2Fbest-practices-in-research-reporting%23loc-statistical-reporting&data=05%7C02%7C%7Ce95631ad7ca14112b37b08ded017703b%7C5a6d5ee56edf4a26ba93f5872dbb9614%7C0%7C0%7C639176996252783299%7CUnknown%7CTWFpbGZsb3d8eyJFbXB0eU1hcGkiOnRydWUsIlYiOiIwLjAuMDAwMCIsIlAiOiJXaW4zMiIsIkFOIjoiTWFpbCIsIldUIjoyfQ%3D%3D%7C0%7C%7C%7C&sdata=VbMQoWc4480s%2FlETcAKGcaCWMhFJHm1nn6vj6pF4c%2B4%3D&reserved=0.

We hope that our revised manuscript file is in accordance with the recommendations

5) [Figure 1,4]: Thank you for providing the Biorender Publication license. Please provide proper Biorender citations and ensure these are also included in the figure legends

We have provided the proper Biorender citations with the respective URL in the figure legends for Fig.1a and Fig. 4a. We realized that our Fig. 1a was a previous version of the one we got the certificate for, so we exchanged the panel 1a of Fig. 1. Fig. 1a is now fully compatible with the licence provided. For this reason, a slightly modified version of Fig. 1 has been uploaded.

Code related issues

Per journal policy, if you have generated any custom code during the course of this investigation, please make it available without restrictions. Please ensure that the code is sufficiently well documented and reusable, and that your Data Statement in the Editorial Manager submission system accurately describes where your code can be found.
Please note that we cannot accept sole deposition of code in GitHub, as this could be changed after publication. However, you can archive this version of your publicly available GitHub code to Zenodo. Once you do this, it will generate a DOI number, which you will need to provide in the Data Accessibility Statement (you are welcome to also provide the GitHub access information).

The code version used in this study was uploaded on Zenodo and the code availability statement was updates as requested: "The code used for this study is publicly available on GitHub under https://github.com/jschoeneich/epithelial_cell_diversity, with the version used in this publication (v1.0.1) archived on Zenodo under DOI: 10.5281/zenodo.21107105."
