# Supplementary material for: Developmental factors drive the compartmentalized and discontinuous maturation of the small intestinal epithelium during the early postnatal period
Source: PLoS Biol. 2026 Aug 3;24(8):e3003212. doi: 10.1371/journal.pbio.3003212 (PMC13460745; doi:10.1371/journal.pbio.3003212)
Supplement: Supplementary file 4 [file pbio.3003212.s027.docx]

In the manuscript “*Developmental factors drive the compartmentalised and discontinuous maturation of the small intestinal epithelium during the early postnatal period*”, Schöneich, Dupont, and colleagues investigated how the intestinal epithelial cells in different regions change their characteristics over time in the postnatal period, utilizing substantial transcriptomic and proteomic data analysis. These studies provide new insights into the organization and development of intestinal epithelial cells during this specific period. Notably, they also demonstrate how the influence of microbiota differs during the early pre-weaning period; gene expression was not highly affected by microbiota during this period. Overall, the paper’s new finding is interesting and relevant to the PLOS Biology readership. However, some concerns and questions need to be addressed to strengthen their conclusions.

The points to be addressed are detailed below:

Major points

#1

The authors show that at post-weaning, the alteration of TGF-beta, MAPK, and PI3K signaling coincides with the upregulation of stem cell signature genes. Those alterations of the pathways may link to the maturation of Paneth cells and promote the production of antimicrobial peptides.

To gain insight into how each cell type is affected by these signaling alterations, I recommend displaying the signaling pathway in crypt cells and villus cells (or at the single-cell level, if possible). Since the Notch pathway is also one of the essential pathways for the intestinal stem cell niche, which interacts with JAK-STAT to influence secretory vs absorptive fate, it would be beneficial to include data on how the Notch pathway is altered, providing an overview of the essential signaling.

#2

The authors demonstrated that developmental rather than microbial factors drive global transcriptome changes during the postnatal period. To strengthen this conclusion, authors should compare gene expression profiling among d01, d05, and d10 in germ-free mice, as they did in SPF mice in main Fig. 1c.

Moreover, a previous study has reported that colonization by microbiota just before weaning (day 17) induces Paneth cell differentiation in intestinal epithelial cells (PMID: **36473468**, DOI: 10.1016/j.immuni.2022.11.003). Thus, there is a possibility that microbiota influences the differentiation and maturation of Paneth cells at the late stage of neonatal (2-3 weeks old) and after weaning.

In order to clarify how microbiota contributes to Paneth cell differentiation at different neonatal stages, it would be beneficial to present gene expression profiling from germ-free mice at day 25 and compare it with that of SPF mice on the same day, if available.

Otherwise, it might be better to add ‘early’ to the first subtitle of the result, ‘Developmental rather than microbial factors drive global transcriptome changes during the XXX postnatal period.’

#3

In Figs. 5 and 6, the authors demonstrate how each type of cell undergoes alterations in gene expression and responses during the postnatal period using single-cell RNA sequencing (scRNA-seq).

However, they do not annotate transit-amplifying cells (TA cells), which have distinct features and are considered an independent cell type.

It would be better to annotate TA cells as a separate population and analyze them independently with other cell types, such as stem cells and other differentiated cells (enterocytes, tuft cells, Enteroendocrine cells, and Paneth/goblet cells).

#4

The authors demonstrate the maturation of Paneth cells, goblet cells, and enterocytes over time, along with the expression of antimicrobial peptides, as shown in Figs. 1-4.

Since this finding is key to this paper, it would be beneficial to show the expression of Defa genes or Reg3a, 3b, or 3g genes, which were identified as altered genes in Figs. 1-4 over time.

By adding this data to the heat map for Paneth cells, goblet cells, or enterocytes in Fig.6e and g, it would also make it much more straightforward to determine whether Paneth/Goblet cells or enterocytes mature and start to express antimicrobial genes. Otherwise, the possibility that the differential gene expressions of antimicrobial peptides arise from the altered proportions of these cells which currently cannot be excluded.

Minor points;

#1

In Fig.3a, it would be nice if you could draw and show how the tissue was micro-dissected with a laser in the images.

#2

In Fig.3c, dots representing Tdrp, Meltf, Mamdc4, Cyp2b10, and Igkc are exactly in the same position between Crypts and Villi volcanoes.

Could you please check if there's an issue with the data processing?

#3

In Fig. 1a, it is mentioned that no samples for SPF (P/M/D) from d25 were analyzed; however, in Fig. 4c, data for SPF (P/M/D) from d25 are shown.

Please clarify.

#4

At line number 10 on page 14, the authors discussed that the decrease in the transforming growth factor (TGF)-beta after weaning is most likely due to the loss of TGF-beta from breast milk.

However, the TGF-beta signaling in epithelial cells can also be influenced by the surrounding cells of the crypts and villi, including mesenchymal cells, which secrete BMP proteins.

It would be better to tone down your discussion.

#5

Salmonella infection can alter the expression of genes involved in tight junctions in intestinal epithelial cells and their localization, thereby changing the susceptibility of the intestine to pathogens.

Specifically, the gene expression of key tight junction proteins, such as E-cadherin, occludin, claudins, and ZO-1, is reported to change in adult intestinal epithelial cells from adult mice (PMID: **32329192**_DOI: 10.1111/cmi.13211). Did you also see those changes in the transcriptome of the neonatal intestine?
