# Supplementary material for: Developmental factors drive the compartmentalized and discontinuous maturation of the small intestinal epithelium during the early postnatal period
Source: PLoS Biol. 2026 Aug 3;24(8):e3003212. doi: 10.1371/journal.pbio.3003212 (PMC13460745; doi:10.1371/journal.pbio.3003212)
Supplement: Peer review history

# Editor report: Decision Letter 0

**DOI:** 10.1371/journal.pbio.3003212.r001

**Authors:** Ines Alvarez-Garcia

12 May 2025

Dear Dr Hornef,

Thank you for submitting your manuscript entitled "Developmental factors drive the compartmentalised and discontinuous maturation of the small intestinal epithelium during the early postnatal period" for consideration as a Research Article by PLOS Biology.

Your manuscript has now been evaluated by the PLOS Biology editorial staff as well as by an academic editor with relevant expertise and I am writing to let you know that we would like to send your submission out for external peer review.

However, before we can send your manuscript to reviewers, we need you to complete your submission by providing the metadata that is required for full assessment. To this end, please login to Editorial Manager where you will find the paper in the 'Submissions Needing Revisions' folder on your homepage. Please click 'Revise Submission' from the Action Links and complete all additional questions in the submission questionnaire.

Once your full submission is complete, your paper will undergo a series of checks in preparation for peer review. After your manuscript has passed the checks it will be sent out for review. To provide the metadata for your submission, please Login to Editorial Manager (https://www.editorialmanager.com/pbiology) within two working days, i.e. by May 14 2025 11:59PM.

If your manuscript has been previously peer-reviewed at another journal, PLOS Biology is willing to work with those reviews in order to avoid re-starting the process. Submission of the previous reviews is entirely optional and our ability to use them effectively will depend on the willingness of the previous journal to confirm the content of the reports and share the reviewer identities. Please note that we reserve the right to invite additional reviewers if we consider that additional/independent reviewers are needed, although we aim to avoid this as far as possible. In our experience, working with previous reviews does save time.

If you would like us to consider previous reviewer reports, please edit your cover letter to let us know and include the name of the journal where the work was previously considered and the manuscript ID it was given. In addition, please upload a response to the reviews as a 'Prior Peer Review' file type, which should include the reports in full and a point-by-point reply detailing how you have or plan to address the reviewers' concerns.

During the process of completing your manuscript submission, you will be invited to opt-in to posting your pre-review manuscript as a bioRxiv preprint. Visit http://journals.plos.org/plosbiology/s/preprints for full details. If you consent to posting your current manuscript as a preprint, please upload a single Preprint PDF.

Feel free to email us at plosbiology@plos.org if you have any queries relating to your submission.

Kind regards,

Ines

--

Ines Alvarez-Garcia, PhD

Senior Editor

PLOS Biology

ialvarez-garcia@plos.org

# Peer review: Decision Letter 1

**DOI:** 10.1371/journal.pbio.3003212.r002

**Authors:** Ines Alvarez-Garcia

25 Jun 2025

Dear Dr Hornef,

Thank you for your patience while your manuscript entitled "Developmental factors drive the compartmentalised and discontinuous maturation of the small intestinal epithelium during the early postnatal period" was peer-reviewed at PLOS Biology. Please accept again my apologies for the delay in sending you our decision. The manuscript has now been evaluated by the PLOS Biology editors, an Academic Editor with relevant expertise, and by four independent reviewers.

The reviews are attached below. As you will see, the reviewers find the conclusions interesting, but they also raise several issues that would need to be addressed before we can consider the manuscript for publication. Reviewer 1 thinks that the paper should focus on temporal analysis of the key villus zonated genes, describe the crypt and villus dynamic expression of the main EEC genes of the dataset, and analyse the correlation between the transcription signature of each segment at each time point at D25 to determine whether or not all segments mature transcriptionally at the same rates or there are segmental delays, among other issues. Reviewer 2 thinks the data on how the Notch pathway is altered in the intestinal stem cell niche should be included, compare gene expression among different developmental stages postnatal of germ-free mice, and show the expression of Defa or Reg3a, 3b or 3g genes to be able to determine whether Panethc/Goblet cells or enterocytes mature and start to express antimicrobial genes. Reviewers 3 and 4 have very similar views and note that some of the claims are not sufficiently supported by the evidence presented and suggest additional analyses to confirm them. In addition, Reviewer 4 thinks the cell viability issue should be addressed by optimising the experimental setup and perform a pseudotime analysis to compare age-dependent differentiation trajectories.

In light of the reviews, we would like to invite you to revise the work to thoroughly address the reviewers' reports. Given the extent of revision needed, we cannot make a decision about publication until we have seen the revised manuscript and your response to the reviewers' comments. Your revised manuscript is likely to be sent for further evaluation by all or a subset of the reviewers.

In addition to these revisions, you will need to complete some formatting changes, which you will receive in a follow up email. A member of our team will be in touch with a set of requests shortly.

We expect to receive your revised manuscript within 3 months. Please email us (plosbiology@plos.org) if you have any questions or concerns, or would like to request an extension.

At this stage, your manuscript remains formally under active consideration at our journal; please notify us by email if you do not intend to submit a revision so that we may withdraw it.

**IMPORTANT - SUBMITTING YOUR REVISION**

Your revisions should address the specific points made by each reviewer. Please submit the following files along with your revised manuscript:

1. A 'Response to Reviewers' file - this should detail your responses to the editorial requests, present a point-by-point response to all of the reviewers' comments, and indicate the changes made to the manuscript.

*NOTE: In your point-by-point response to the reviewers, please provide the full context of each review. Do not selectively quote paragraphs or sentences to reply to. The entire set of reviewer comments should be present in full and each specific point should be responded to individually, point by point.

You should also cite any additional relevant literature that has been published since the original submission and mention any additional citations in your response.

2. In addition to a clean copy of the manuscript, please also upload a 'track-changes' version of your manuscript that specifies the edits made. This should be uploaded as a "Revised Article with Changes Highlighted" file type.

3. Resubmission Checklist

When you are ready to resubmit your revised manuscript, please refer to this resubmission checklist: https://plos.io/Biology_Checklist

To submit a revised version of your manuscript, please go to https://www.editorialmanager.com/pbiology/ and log in as an Author. Click the link labelled 'Submissions Needing Revision' where you will find your submission record.

Please make sure to read the following important policies and guidelines while preparing your revision and fulfil the editorial requests:

a) *PLOS Data Policy*

Please note that as a condition of publication PLOS' data policy (http://journals.plos.org/plosbiology/s/data-availability) requires that you make available all data used to draw the conclusions arrived at in your manuscript. If you have not already done so, you must include any data used in your manuscript either in appropriate repositories, within the body of the manuscript, or as supporting information (N.B. this includes any numerical values that were used to generate graphs, histograms etc.). Please also indicate in each figure legend where the data can be found. For an example see here: http://www.plosbiology.org/article/info%3Adoi%2F10.1371%2Fjournal.pbio.1001908#s5

b) *Published Peer Review*

Please note while forming your response, if your article is accepted, you may have the opportunity to make the peer review history publicly available. The record will include editor decision letters (with reviews) and your responses to reviewer comments. If eligible, we will contact you to opt in or out. Please see here for more details:

https://blogs.plos.org/plos/2019/05/plos-journals-now-open-for-published-peer-review/

c) *Blot and Gel Data Policy*

Please provide the original, uncropped and minimally adjusted images supporting all blot and gel results reported in an article's figures or Supporting Information files. We will require these files before a manuscript can be accepted so please prepare them now, if you have not already uploaded them. Please carefully read our guidelines for how to prepare and upload this data: https://journals.plos.org/plosbiology/s/figures#loc-blot-and-gel-reporting-requirements

d) *Protocols deposition*

To enhance the reproducibility of your results, we recommend that if applicable you deposit your laboratory protocols in protocols.io, where a protocol can be assigned its own identifier (DOI) such that it can be cited independently in the future. For instructions see: https://journals.plos.org/plosbiology/s/submission-guidelines#loc-materials

Thank you again for your submission to our journal. We hope that our editorial process has been constructive thus far, and we welcome your feedback at any time. Please don't hesitate to contact us if you have any questions or comments.

Sincerely,

Ines

--

Ines Alvarez-Garcia, PhD

Senior Editor

PLOS Biology

ialvarez-garcia@plos.org

------------------------------------

Reviewers' comments

Rev. 1: Shalev Itzkovitz - note that this reviewer has signed his review

The manuscript provides a comprehensive resource for exploring neonatal maturation of the mouse intestine. It combines bulk transcriptomics, proteomics, LCMseq and scRNAseq at key time points before and after weaning. The experiments and analyses are generally well performed. It is hard to obtain a clear take-home message from the paper, but it will be useful to the community as a resource. The paper can be improved in several aspects in a revision:

- The paper focuses on epithelial lineages, what about stromal and immune lineages? At what stages are macrophages and lymphocytes established? Are there temporal variations in endothelial and fibroblast lineages?

- The paper focuses on crypt vs. villus, however the villus is a highly heterogeneous structure composed of zonal enterocytes (PMID 30270040, 39112711), and secretory cells (34031373). At what stages are these villus zonal sub-populations established? The authors should focus on temporal analysis of the key villus zonated genes.

- One of the most interesting cellular populations in the gut are enteroendocrine cells, which exhibit strong zonation both along the crypt-villus axis and across intestinal segments (PMID 30712869, 30038251). The authors should describe the crypt and villus dynamic expression of the key EEC genes in their temporally-resolved dataset.

- Segmental maturation - Figure 4 - the authors should compute the correlation between the transcriptomic signature of each segment at each time point with that at D25 to determine whether all segments transcriptomically mature at the same rates, or whether there are segmental delays.

Minor comments:

- Throughout the paper, dot plots of enriched functions are too cluttered and are hard to comprehend. The authors should focus on the most stringent pathways and rotate the plots so that the pathways are horizontal (rather than diagonal). They can provide the complete set of enriched pathways in supplementary tables.

- Fonts in some figures are too small - e.g. Figure 5e,g,i. Better to show representative genes from each cluster with larger font.

- Figure 2b - add Spearman correlation values and pvalues between RNA and proteins.

- Proteome analysis - do the authors identify pancreatic proteins? Such as trypsin, amylase and lipase?

Rev. 2:

In the manuscript “Developmental factors drive the compartmentalised and discontinuous maturation of the small intestinal epithelium during the early postnatal period”, Schöneich, Dupont, and colleagues investigated how the intestinal epithelial cells in different regions change their characteristics over time in the postnatal period, utilizing substantial transcriptomic and proteomic data analysis. These studies provide new insights into the organization and development of intestinal epithelial cells during this specific period. Notably, they also demonstrate how the influence of microbiota differs during the early pre-weaning period; gene expression was not highly affected by microbiota during this period. Overall, the paper’s new finding is interesting and relevant to the PLOS Biology readership. However, some concerns and questions need to be addressed to strengthen their conclusions.

The points to be addressed are detailed below:

Major points

#1

The authors show that at post-weaning, the alteration of TGF-beta, MAPK, and PI3K signaling coincides with the upregulation of stem cell signature genes. Those alterations of the pathways may link to the maturation of Paneth cells and promote the production of antimicrobial peptides.

To gain insight into how each cell type is affected by these signaling alterations, I recommend displaying the signaling pathway in crypt cells and villus cells (or at the single-cell level, if possible). Since the Notch pathway is also one of the essential pathways for the intestinal stem cell niche, which interacts with JAK-STAT to influence secretory vs absorptive fate, it would be beneficial to include data on how the Notch pathway is altered, providing an overview of the essential signaling.

#2

The authors demonstrated that developmental rather than microbial factors drive global transcriptome changes during the postnatal period. To strengthen this conclusion, authors should compare gene expression profiling among d01, d05, and d10 in germ-free mice, as they did in SPF mice in main Fig. 1c.

Moreover, a previous study has reported that colonization by microbiota just before weaning (day 17) induces Paneth cell differentiation in intestinal epithelial cells (PMID: 36473468, DOI: 10.1016/j.immuni.2022.11.003). Thus, there is a possibility that microbiota influences the differentiation and maturation of Paneth cells at the late stage of neonatal (2-3 weeks old) and after weaning.

In order to clarify how microbiota contributes to Paneth cell differentiation at different neonatal stages, it would be beneficial to present gene expression profiling from germ-free mice at day 25 and compare it with that of SPF mice on the same day, if available.

Otherwise, it might be better to add ‘early’ to the first subtitle of the result, ‘Developmental rather than microbial factors drive global transcriptome changes during the XXX postnatal period.’

#3

In Figs. 5 and 6, the authors demonstrate how each type of cell undergoes alterations in gene expression and responses during the postnatal period using single-cell RNA sequencing (scRNA-seq).

However, they do not annotate transit-amplifying cells (TA cells), which have distinct features and are considered an independent cell type.

It would be better to annotate TA cells as a separate population and analyze them independently with other cell types, such as stem cells and other differentiated cells (enterocytes, tuft cells, Enteroendocrine cells, and Paneth/goblet cells).

#4

The authors demonstrate the maturation of Paneth cells, goblet cells, and enterocytes over time, along with the expression of antimicrobial peptides, as shown in Figs. 1-4.

Since this finding is key to this paper, it would be beneficial to show the expression of Defa genes or Reg3a, 3b, or 3g genes, which were identified as altered genes in Figs. 1-4 over time.

By adding this data to the heat map for Paneth cells, goblet cells, or enterocytes in Fig.5e and g, it would also make it much more straightforward to determine whether Paneth/Goblet cells or enterocytes mature and start to express antimicrobial genes. Otherwise, the possibility that the differential gene expressions of antimicrobial peptides arise from the altered proportions of these cells which currently cannot be excluded.

#5

In Figs. 5d and e, it would be better to characterise each cluster of absorptive enterocytes. Showing genes, such as Reg1, Slc2a2, and Ada, that are preferentially expressed in each zone along the crypt-villus epithelial axis will help to define the clusters. Information described in PMID 30270040 will be helpful.

#6

In Figs. 5f and g, it would be better to define which cluster corresponds to Paneth cells or Goblet cells, since they are distinct and defined cell types. It would be possible to separate goblet cells and Paneth cells by defined markers, such as Ag16 and Lyz1.

Minor points;

#1

In Fig.3a, it would be nice if you could draw and show how the tissue was micro-dissected with a laser in the images.

#2

In Fig.3c, dots representing Tdrp, Meltf, Mamdc4, Cyp2b10, and Igkc are exactly in the same position between Crypts and Villi volcanoes.

Could you please check if there's an issue with the data processing?

#3

In Fig. 1a, it is mentioned that no samples for SPF (P/M/D) from d25 were analyzed; however, in Fig. 4c, data for SPF (P/M/D) from d25 are shown.

Please clarify.

#4

At line number 10 on page 14, the authors discussed that the decrease in the transforming growth factor (TGF)-beta after weaning is most likely due to the loss of TGF-beta from breast milk.

However, the TGF-beta signaling in epithelial cells can also be influenced by the surrounding cells of the crypts and villi, including mesenchymal cells, which secrete BMP proteins.

It would be better to tone down your discussion.

#5

Salmonella infection can alter the expression of genes involved in tight junctions in intestinal epithelial cells and their localization, thereby changing the susceptibility of the intestine to pathogens.

Specifically, the gene expression of key tight junction proteins, such as E-cadherin, occludin, claudins, and ZO-1, is reported to change in adult intestinal epithelial cells from adult mice (PMID: 32329192_DOI: 10.1111/cmi.13211). Did you also see those changes in the transcriptome of the neonatal intestine?

Rev. 3:

The manuscript "Developmental factors drive the compartmentalised and discontinuous maturation of the small intestinal epithelium during the early postnatal period", by Schöneich et al. characterizes transcriptional and proteomic changes in the mouse small intestinal epithelium at various stages of neonatal development. The key findings described in the manuscript provide new insight into the region- and temporal-specific changes to the small intestine during development that could provide the basis for new studies regarding neonatal development and host-microbe interactions. The manuscript would be an informative study for readers but requires additional work - considerations are provided below.

P15 - Maternally acquired proteins in the pre-weaning epithelium revealed by proteome analysis

The authors' title for this section should be revised as this section highlights major changes in the proteome of the small intestine epithelium between pre- (day 10) and post-wean (day 25) and between day 1 and day 5 (maternally acquired proteins). Given the robust changes in the proteome at both timepoints, the title should be changed to reflect the observation at both timepoints.

The authors claim that the change in proteome between day 1 and day 5 of the small intestinal epithelium is due maternal milk-derived proteins. This claim is supported by scRNA-seq data from breast tissue of Tabula Muris that shows breast luminal cells express genes of proteins identified by proteome analysis, but not RNA-seq. The aggregated expression of the 135 genes by UMAP and violin plot in luminal cells is an issue, as it obscures the degree to which these potentially maternally-derived proteins are transcriptionally expressed. Re-visualizing these data as a heatmap with all genes plotted against each cluster as a supplemental figure is recommended. In addition, these data are only correlative and do not provide sufficient evidence that the increase in proteins at day 5 are derived maternally from breast milk. As such, moving these scRNA-seq data as supplemental data would be appropriate.

P18 - "Additionally, the formation of deeper crypts during the late postnatal period may affect the metabolic and transport capabilities of the post-weaning crypt epithelium, due to reduced contact with dietary substrates." This statement provides an explanation as to why the post-wean crypts are associated with a decrease in the lipid metabolism gene signature, but it is contradicted by similar decreases observed in post-wean villi (Fig 3e). As this explanation is speculative and the mirrored decreases in the villi likely reflect a general change in dietary milk, removing this sentence from the results is recommended.

P18 - "Age-dependent compartmentalisation of gene expression along the crypt-villus axis"

The authors use laser capture microdissection (LMD) to characterize the age-dependent changes in gene expression that occurs within the crypt/intervillus and villus regions. The authors identified broad transcriptomic changes in the expression of genes associated with host-defense, lipid and triglyceride metabolism, translation activity, stemness (Olfm4), and cellular turnover. Given that these broad changes in transcriptome are also associated with changes in epithelial cell subsets, the authors should expand upon this idea by providing quantified immunofluorescence staining of some key epithelial cell subsets (ie. stem cells (Olfm4), transit-amplifying cells (Mki67/Pcna, Paneth cells (Lyz1/UEA1), goblet cells (Muc2)) and/or candidate genes of interest (ie. Reg3a)).

P19 - "In contrast, increased expression of the IgA transport molecule polymeric immunoglobulin receptor (pIgR) in both post-weaning crypt and villus epithelium was consistent with the increase of IgA-secreting plasma cells occurring at weaning (Fig. 3c, Fig. S3b-c, Suppl. Table 6)" - The argument for an increase in the abundance of IgA-secreting plasma cells in the villus post-weaning not sufficiently supported as it is based solely on gene expression data of LMD-isolated villi. I would suggest additional corroborative data such as quantifying the number of B cells present within the pre- and post-wean villi.

P19-20 "Transcriptional differences along the proximal-to-distal length of the small intestine"

The authors demonstrate that the proximal, medial, and distal intestine undergo region-specific transcriptomic changes that are dependent on age. The authors further aggregate genes of similar expression pattern and label these as 'clusters' while describing their overall function based on GO term enrichment and the expression of key genes of interest. This section would benefit with more interpretation with reference to the study by Zwick et. al. titled "Epithelial zonation along the mouse and human small intestine defines five discrete metabolic domains" (https://doi.org/10.1038/s41556-023-01337-z). Zwick et. al. demonstrate that the small intestinal epithelium can be divided along the proximal-distal axis into 5 metabolic domains. It would be of interest to the field to determine if these region-dependent metabolic domains are present pre- or post-weaning.

P20 - "Age- and cell type-specific developmental trajectories revealed by single cell transcriptional analysis"

P10 & Suppl. Fig 5 - For scRNA-seq QC metrics, the authors state that UMIs with >25% mitochondria gene expression were removed. With respect to enterocytes and intestinal stem cells, these two cell subsets by scRNA-seq and other methods have been well characterized to generate more mitochondria due to their metabolic activity. As such, it is generally not recommended filter IECs based on mitochondrial gene expression. The authors need to revisit their scRNA-seq analysis to assess if this cutoff is removing key epithelial cells from their analysis with respect to the day 10 and day 25 samples.

Figure 5 & Suppl. Fig 5 - Based on the heatmaps (Fig 5E, G) QC data presented, the authors post-wean (day 25) sample captured a critically small fraction of crypt/villus enterocytes (10-30% compared to other timepoints) along with decreased unique features/genes expressed compared to other timepoints. In addition, no stem cells were captured, while there is a 2:1 ratio of enterocytes to secretory cells. This composition of cells captured for this timepoint contradicts other parts of the manuscript which suggest post-weaning intestinal crypts become more defined and develop Paneth cells (ie. stem cells and Paneth cells should be captured). These limitations raise concerns regarding the quality of the data for this sample and suggest the findings based on this sample should be removed from the manuscript or at minimum the caveats of these conclusions should be clearly discussed in the results or discussion.

P20 - "Notably, epithelial cells from adult mice showed an overall lower viability". For the authors' scRNA-seq experiment, they should clarify whether this is based on viability by flow cytometry or scRNA-seq analysis and provide the data.

P20 - "We initially focussed on the effect of age on epithelial cell type differentiation since, although studied in the adult host, little is known about regulatory circuits that drive epithelial cell differentiation during the neonatal and infant period." Highlighted spelling error.

The authors use DoRothEA to predict transcription factor (TF) activity that drive epithelial cell differentiation during neonatal development. While the authors identified prospective TFs that may play a role in driving lineage commitment of enterocytes and secretory epithelial cells, it remains unclear if these TFs drive cell type-specific functions or lineage commitment, given that these associations are presented with 'terminally' differentiated subsets. To better delineate this idea, performing trajectory inference analysis is key to determine where along the predicted differentiation trajectory these TFs are expressed (ie. are these TFs expressed in progenitor population at earlier timepoints or only in progenitor populations post-weaning?).

P21, Figure 5D/F - "A spatial dimension was added to the absorptive enterocyte subclusters by overlapping the gene expression signatures of crypt and villus epithelium previously identified by bulk RNA-Seq (Fig. 3) onto our single cell dataset (Fig. 5d). This allowed the identification of the gene signatures of single absorptive enterocytes along the crypt-villus axis and illustrated the continuous gradient of cell differentiation and functional diversity… A similar phenomenon was also observed for goblet/Paneth cells." The authors infer the trajectory of enterocytes and goblet/Paneth cells based on their previously identified crypt-villus axis gene signatures and defined markers of goblet and Paneth cells that illustrate a gradient of expression that progress through respective subclusters. A key limitation of this analysis is that the authors have separately subclustered enterocytes and secretory lineages when the differentiation of stem cells to enterocytes and secretory lineages occurs in tandem. Moreover, inferring trajectory based on expression gradients of defined genes/gene sets superimposed over subclusters is incorrect, as it assumes subclusters are defined and ordered on the same principles, when their defining genes could be unrelated to their lineage commitment. As such, the authors should perform trajectory inference analysis for each of their respective timepoints to better support this idea.

P22 - "The global and cell type-specific gene response to enteric infection"

P22 - "UMAPs representing the scRNA-Seq of the uninfected (d05) and infected datasets (d05i) illustrated a marked decrease in the abundance of absorptive enterocytes in 5-day-old infected animals." Missing figure reference.

P22 - "DE gene analysis of day 5 uninfected and day 5 infected samples revealed upregulation of typical epithelial antimicrobial host response genes such as c-type lectins (e.g. Reg3g, Reg3b, Reg3a)". The authors demonstrate that the pre-wean epithelium under steady-state does not express antimicrobial/host defense genes, but that these genes are upregulated post-wean (C-type lectins and defensins). Interestingly, these data suggest the neonatal epithelium has the potential to express host defense genes if required. Moreover, the scRNA-seq of infected and uninfected day 5 epithelium suggest that the increase in Reg3g/a expression with infection occurs in all epithelial lineages (Suppl. Fig 6). These data suggest that the neonatal epithelium may rely on c-type lectins in response to enteric infections, as opposed to defensins from Paneth cells that arise post-weaning. As such, the importance of investigating enteric infections in the neonatal epithelium could be emphasized and improved by expanding upon this distinction in this section of the results or within the discussion.

P23 - "whereas absorptive enterocytes were associated with a higher cell division rate (e.g. "chromosome segregation", GO:0007059) and goblet/Paneth cells with a higher innate immune response orchestrated by pattern recognition receptors (e.g. "activation of innate immune response", GO: 0002218)." The authors perform differential gene expression analysis and GO term enrichment between uninfected and infected enterocytes and identify many differentially expressed genes and enriched GO terms (Suppl Fig 6a, Fig 6e). A limitation of this comparison as indicated by Fig. 6c is that differential expression compares 81 infected to 1339 uninfected enterocytes. Given the imbalance the number of enterocytes compared, the expression profile of infected enterocytes id likely not representative. This comparison should be removed.

Discussion

P25 - "Many largely uncharacterised transcription factors were significantly associated with specific epithelial cell types in the neonate and await further analysis." The purpose/message of this paragraph in the discussion is unclear. The authors state the identified TFs that may drive neonatal epithelial differentiation and speculate that these TFs may play additional roles in restitution from inflammation and regulating various gradients in the small intestine. However, the importance of this idea with respect to neonatal development or intestinal physiology needs further exploration and explanation.

Rev. 4:

Summary: In this manuscript, the authors present a multi-omics analysis of the mouse intestinal epithelium across various anatomical regions and developmental time points.

The resulting molecular atlas offers novel insights into:

1. transcriptional changes in intestinal epithelial cells during the postnatal period driven by developmental factors,

2. non-transcriptional regulation of proteins between day 1 and day 5 after birth,

3. age-dependent transcriptional changes along the proximal-to-distal and crypt-villus axes,

4. cell type-specific single-cell transcriptional dynamics during postnatal development,

5. transcriptional alterations in specific cell types in response to infection.

This study holds significant potential, as it provides a spatially resolved multi-omics resource for characterizing molecular changes within the mouse intestinal epithelium driven by developmental and microbial cues. However, several major issues need to be addressed to fully support the authors' claims.

Major points:

1. Figure 1f: It is not clear how the authors evaluated the significance of correlations between the two sets of genes.

2. Figure 2: The proteomic changes between day 1 and day 5 appear to differ from the transcriptomic changes. The authors suggest that this discrepancy may be due to non-transcriptional regulation of proteins on day 5. However, the correlation between protein and gene expression levels on day 5 appears to be comparable to that of other time points (Figure 2b). This inconsistency should be carefully investigated.

3. On page 20, the sentence "Notably, epithelial cells from adult mice showed an overall lower viability" is ambiguous. The authors should clarify what type of variability was measured and provide appropriate supporting evidence.

4. The scRNA-seq data generated in this study appears to be of suboptimal quality. In particular, intestinal stem cells at postnatal day 10 and day 25 seem to be underrepresented, likely due to issues with cell viability. Although the authors have acknowledged this limitation, the depletion of intestinal stem cells could significantly compromise the accuracy of differentiation trajectory analyses. Notably, the authors failed to demonstrate age-dependent differentiation trajectories starting from intestinal stem cells. Figure 5e and 5g do not clearly depict such trajectories. Additionally, the meaning of the subcluster ordering in the heatmaps is unclear. Given that intestinal stem cells have been successfully captured in other studies, the authors should more thoroughly address the cell viability issue by optimizing their experimental protocols. Furthermore, they should perform a pseudotime analysis to rigorously compare age-dependent differentiation trajectories.

5. The scRNA-seq data lack biological replicates, which raises concerns about the reproducibility and robustness of the results presented in Figure 5 and 6. Given the suboptimal quality of the dataset, at least one additional biological replicate should be generated to support the conclusions. Alternatively, the transcriptional changes observed in Figures 1 and 3 could be recapitulated by aggregating single cells into pseudobulk profiles to validate the quality of the scRNA-seq data.

Minor comments:

1. Figure 1d, S1e, S2c: Y-axis labels should be provided.

2. Figure 1e, S2d: Representative genes should be highlighted.

3. Define "UMAP" thought the manuscript.

Attachment Submitted filename: Review_PLOS_Biology_06062025.docx

# Author comment: Author response to Decision Letter 2

**DOI:** 10.1371/journal.pbio.3003212.r003

2 Dec 2025

Attachment Submitted filename: Response to reviewers file_1-12-2025.pdf

# Peer review: Decision Letter 2

**DOI:** 10.1371/journal.pbio.3003212.r004

**Authors:** Ines Alvarez-Garcia

28 Jan 2026

Dear Dr Hornef,

Thank you for your patience while we considered your revised manuscript entitled "Developmental factors drive the compartmentalised and discontinuous maturation of the small intestinal epithelium during the early postnatal period" for publication as a Research Article at PLOS Biology. Your revised study has been evaluated by the PLOS Biology editors, the Academic Editor and the four original reviewers.

The reviews are attached below. You will see that while some of the reviewers are mostly satisfied with the revision, others raise several concerns that should be addressed before we can consider the manuscript for publication. Reviewer 1 thinks a discussion should be added on developmental dynamics of stromal cells and immune cells, and also include additional plots to track the changes in key enterocyte markers over time and explain how these relate to changes in diet/exposure to bacteria, among other requests. Reviewer 2 has similar thoughts and finds controversial the interpretation that downregulation of Lrp2 after weaning maybe linked to the high metabolic demand imposed by the lipid-enriched milk diet of the newborn. This reviewer would like to see a revision of the discussion according to the presented data and consider the fact that Goblet and Paneth cells don’t possess absorptive functions and reinterpret their results, and points out that Fig. S2 and S3 are the same, thus please make sure that the correct Fig. S3 is uploaded in the new revision. Reviewer 3 is satisfied, whereas Reviewer 4 mentions that there is no attempt to optimise the single-cell isolation protocols, which are biased toward capturing viable preweaning epithelia cells and are suboptimal for isolating viable adult intestinal epithelial cells (points 4 & 5 of the previous review). Thus, this reviewer thinks that the comparative analyses across the developmental time points are limited. The reviewer would like to see a demonstration of the robustness and reproducibility of the conclusions drawn from these data.

In light of the reviews, and after further discussion with the Academic Editor. we would like to invite you to revise one more time the work to address the reviewers' reports making sure that, at minimum, these points are thoroughly addressed:

1) scRNA-seq dataset quality control: a rigorous technical justification should be provided for the high mitochondrial content in the late-stage samples and clear evidence of reproducibility should be presented across biological replicates, to ensure that the dataset reflects biologically intact cells.

2) Improved temporal presentation of key markers: aggregated summaries should be replaced with high-resolution, gene-specific temporal plots for key antimicrobial, transporter, and hormone markers, enabling clearer links between molecular transitions and physiological milestones such as weaning and dietary changes.

3) Refined narrative on metabolic transitions: the metabolic transition from the prenatal to post-weaning period should be clarified as a shift from a “primed/regulatory” state to an “active execution” of lipid metabolism, while noting that the metabolic profile observed in secretory cells likely reflects bioenergetic demand rather than genuine absorptive function.

In addition, we would like to consider the paper as a Resource, thus we have changed the format to Methods and Resources.

Given the extent of revision needed, we cannot make a decision about publication until we have seen the revised manuscript and your response to the reviewers' comments. Your revised manuscript could be sent for further evaluation by all or a subset of the reviewers.

In addition to these revisions, you will need to complete some formatting changes, which you will receive in a follow up email. A member of our team will be in touch with a set of requests shortly.

We expect to receive your revised manuscript within 3 months. Please email us (plosbiology@plos.org) if you have any questions or concerns, or would like to request an extension.

At this stage, your manuscript remains formally under active consideration at our journal; please notify us by email if you do not intend to submit a revision so that we may withdraw it.

**IMPORTANT - SUBMITTING YOUR REVISION**

Your revisions should address the specific points made by each reviewer. Please submit the following files along with your revised manuscript:

1. A 'Response to Reviewers' file - this should detail your responses to the editorial requests, present a point-by-point response to all of the reviewers' comments, and indicate the changes made to the manuscript.

*NOTE: In your point-by-point response to the reviewers, please provide the full context of each review. Do not selectively quote paragraphs or sentences to reply to. The entire set of reviewer comments should be present in full and each specific point should be responded to individually, point by point.

You should also cite any additional relevant literature that has been published since the original submission and mention any additional citations in your response.

2. In addition to a clean copy of the manuscript, please also upload a 'track-changes' version of your manuscript that specifies the edits made. This should be uploaded as a "Revised Article with Changes Highlighted" file type.

3. Re-submission Checklist*

When you are ready to resubmit your revised manuscript, please refer to this re-submission checklist: https://plos.io/Biology_Checklist

To submit a revised version of your manuscript, please go to https://www.editorialmanager.com/pbiology/ and log in as an Author. Click the link labelled 'Submissions Needing Revision' where you will find your submission record.

Thank you again for your submission to our journal. We hope that our editorial process has been constructive thus far, and we welcome your feedback at any time. Please don't hesitate to contact us if you have any questions or comments.

Sincerely,

Ines

--

Ines Alvarez-Garcia, PhD

Senior Editor

PLOS Biology

ialvarez-garcia@plos.org

------------------------------------

Reviewers' comments

Rev. 1:

The authors have addressed my comments with new analyses and figures. Some of these are great and add value to the paper, while others should be improved for clarity:

1) Developmental dynamics of stromal cells and immune cells - the authors mention other manuscripts that explore these populations, this is ok, but the authors should mention this in the discussion as a future direction or refer to previous work.

2) My point 2 - The new analyses is insufficient, the authors should include additional plots in the format of Figure 1j (median expression over time or mean+-se expression over time) that will enable explicitly tracking the changes in key enterocyte markers over time - the antimicrobial programs Ccl25, Il18, Lypd8, Nlrp6, Reg3a, Reg3b, Reg3g, the amino acid/peptide transporters Slc7a7, Slc7a8, Slc7a9, Slc15a1, the glucose tranporters Slc2a2, Slc2a5, Slc5a1, the lipid-cholesterol programs Npc1l1, Apoa1, Apoa4, Apob, Dgat1, Dgat2, Cd36 and the purine villus tip programs Slc28a2, Nt5e, Enpp3 and ada. Do any of these increase/decrease at specific time points, how could this be related to changes in diet/exposure to bacteria? As it stands Figure 3c shows the summed expression of dozens of markers and does not facilitate the biological insights that could be gained by examining the temporal profiles of such specific key genes, which have direct physiological function.

3) Dynamics of key EEC hormones - Thank you for adding this, please also show as plots of median vs. time or mean+-SE vs time, Figure 3g makes it hard to follow the trends. Please discuss the trends and their potential physiological significance, e.g. it seems that Gip, associated with satiety and insulin secretion strongly increases in the villi enterocytes only at day 25 whereas Sct and Cck seem to decrease from day 1 to day 25? Please quantify changes (you can also plot a heatmap of the hormone median levels vs. developmental time sorted from early to late). Some discussions on the trends would greatly add to the manuscript. Are these related to pancreas maturations, changes in diet, weaning etc?

For future revisions please paste all relevant responses (new text + figures) under the respective points in the response to reviewer comment file, this would greatly assist in the review process. E.g., when a reviewer faces a sentence such as: "Moreover, cell type marker genes including marker genes for EECs were included in Fig. 1-4 (new Fig.1i, new Fig. 2g, new Fig. 3g, new Fig. 4j)." it is very difficult to track the changes made. Please help reviewers by making the response file more accessible.

Rev. 2:

The authors have addressed many of my previous concerns and, overall, the data are sufficient for publication. However, the authors should address the following points, including a reconsideration of the interpretation of the two aspects of the Results.

#1

Fig.S3 was not successfully uploaded. Fig. S2 appears to have been uploaded in place of Fig. S3. Please upload Fig.S3 correctly.

#2

In the Results section (page 15, line 15; page 21, line 14), the authors suggest that downregulation of Lrp2 after weaning, or decreased expression of genes represented by GO terms related to lipid metabolism, may be linked to the high metabolic demand imposed by the lipid-enriched milk diet of the newborn. This interpretation appears controversial in light of the presented data.

Specifically, the upregulation of the fatty acid metabolic process GO term after weaning (Fig. 1h, 3i, 4h, 5e) suggests that intestinal cells are metabolically adapted to lipid-containing food after weaning. Based on current understanding, fatty acid metabolic process represents the active execution of fatty acid metabolism, whereas regulation of lipid metabolic process represents a preparatory or modulatory layer that adjusts lipid metabolic capacity in response to environmental or developmental cues.

From this perspective, the data indicate that during the preweaning stage, the intestine may regulate or sense lipid metabolic state, but does not necessarily execute high levels of lipid metabolism. In contrast, after weaning, intestinal epithelial cells appear poised to actively metabolize lipids.

Accordingly, the observed shift from enrichment of regulation of lipid metabolic process in crypt cells of the prenatal intestine (Fig. 3h) to enrichment of fatty acid metabolic process in bulk cells (Fig. 1h, 4g,h), villus cells (Fig. 3i), and enterocytes (Fig. 5e) likely reflects a developmental transition from a preparatory metabolic state to active lipid metabolism. The enrichment of regulation of lipid metabolic process in immature intestinal epithelial cells (Fig. 3h) may therefore represent a primed state in which cells respond to environmental cues and become poised for subsequent high lipid metabolic demand prior to full activation of lipid metabolic pathways (Fig. 1h, 3i).

In this context, exposure to milk during the preweaning stage could promote intestinal functional maturation and contribute to the emergence of more mature enterocytes. I therefore suggest that the authors reconsider and revise the discussion of these findings in the Results section to better align with the presented data.

#3

Goblet and Paneth cells are not known to possess absorptive functions, although they can sense nutrients. Therefore, the statement that GO term analysis of differentially expressed genes revealed increased absorption with increasing age (Fig. 6c; Supplementary Table 14; page 26, line 10) may lead to misunderstanding.

The observed GO term enrichment may instead reflect increased bioenergetic demand and potential nutrient-handling capacity required to support enhanced secretory and antimicrobial functions in these cell types. Alternatively, possible contributions from ambient enterocyte RNA or cell doublets should be considered.

I recommend revising the authors' interpretation.

Rev. 3:

The authors have addressed my comments fully. Looking forward to seeing this work published as it's an informative study for the field.

Rev. 4:

The revised manuscript does not address my previous concerns regarding the suboptimal quality and reproducibility of the scRNA-seq dataset. I also disagree with the revised quality control criteria, particularly the exclusion of the proportion of mitochondrial genes.

More specifically,

1. The authors did not attempt to optimize their single-cell isolation protocols, which appear to be biased toward capturing viable preweaning epithelia cells and are suboptimal for isolating viable adult intestinal epithelial cells. Given the depletion of intestinal stem cells and TA cells at day 10 and 25, together with the overall suboptimal quality of these samples, comparative analyses across developmental time points are severely limited. As a result, the single-cell dataset has limited value as a resource. Furthermore, the newly added differentiation trajectory analysis of enterocytes appears to reflect spatial zonation along the crypt-villus axis rather than true differentiation trajectories of enterocytes.

2. Given the suboptimal quality of their single-cell data, it is critical to demonstrate the robustness and reproducibility of the conclusions drawn from these data. Although the authors state that the two day-25 replicates are highly similar, no supporting evidence is provided to substantiate this claim.

3. As also noted by Reviewer 3, intestinal stem cells and enterocytes are metabolically active and therefore express mitochondrial genes at relatively high levels to support nutrient transport. However, an excessively high proportion of mitochondrial genes (>20%) typically indicates cytoplasmic leakage during tissue dissociation. In Figure S5d, the majority of cells from day 25, which already exhibit suboptimal quality, display extremely high proportions of mitochondrial genes (>50%), strongly suggesting that these cells are damaged rather than biologically intact.

# Author comment: Author response to Decision Letter 3

**DOI:** 10.1371/journal.pbio.3003212.r005

18 May 2026

Attachment Submitted filename: point by point _2rev.pdf

# Editor report: Decision Letter 3

**DOI:** 10.1371/journal.pbio.3003212.r006

**Authors:** Ines Alvarez-Garcia

19 Jun 2026

Dear Dr Hornef,

Thank you for your patience while we considered your revised manuscript entitled "Developmental factors drive the compartmentalised and discontinuous maturation of the small intestinal epithelium during the early postnatal period" for publication as a Methods and Resources at PLOS Biology. This revised version of your manuscript has been evaluated by the PLOS Biology editors and by the Academic Editor.

Based on our Academic Editor's assessment of your revision, we are likely to accept this manuscript for publication, provided you satisfactorily address the data and other policy-related requests stated below my signature.

As you address these items, please take this last chance to review your reference list to ensure that it is complete and correct. If you have cited papers that have been retracted, please include the rationale for doing so in the manuscript text, or remove these references and replace them with relevant current references. Any changes to the reference list should be mentioned in the cover letter that accompanies your revised manuscript.

In addition to these revisions, you may need to complete some formatting changes, which you will receive in a follow up email. A member of our team will be in touch with a set of requests shortly. If you do not receive a separate email within a few days, please assume that checks have been completed, and no additional changes are required.

We expect to receive your revised manuscript within two weeks.

To submit your revision, please go to https://www.editorialmanager.com/pbiology/ and log in as an Author. Click the link labelled 'Submissions Needing Revision' to find your submission record. Your revised submission must include the following:

- a cover letter that should detail your responses to any editorial requests, if applicable, and whether changes have been made to the reference list

- a Response to Reviewers file that provides a detailed response to the reviewers' comments (if applicable, if not applicable please do not delete your existing 'Response to Reviewers' file.)

- a track-changes file indicating any changes that you have made to the manuscript.

NOTE: If Supporting Information files are included with your article, note that these are not copyedited and will be published as they are submitted. Please ensure that these files are legible and of high quality (at least 300 dpi) in an easily accessible file format. For this reason, please be aware that any references listed in an SI file will not be indexed. For more information, see our Supporting Information guidelines:

https://journals.plos.org/plosbiology/s/supporting-information

*Published Peer Review History*

Please note that you may have the opportunity to make the peer review history publicly available. The record will include editor decision letters (with reviews) and your responses to reviewer comments. If eligible, we will contact you to opt in or out. Please see here for more details:

https://plos.org/published-peer-review-history/

*Press*

Should you, your institution's press office or the journal office choose to press release your paper, please ensure you have opted out of Early Article Posting on the submission form. We ask that you notify us as soon as possible if you or your institution is planning to press release the article.

*Protocols deposition*

To enhance the reproducibility of your results, we recommend that if applicable you deposit your laboratory protocols in protocols.io, where a protocol can be assigned its own identifier (DOI) such that it can be cited independently in the future. Additionally, PLOS ONE offers an option for publishing peer-reviewed Lab Protocol articles, which describe protocols hosted on protocols.io. Read more information on sharing protocols at https://plos.org/protocols?utm_medium=editorial-email&utm_source=authorletters&utm_campaign=protocols

Please do not hesitate to contact me should you have any questions.

Sincerely,

Ines

--

Ines Alvarez-Garcia, PhD

Senior Editor

PLOS Biology

ialvarez-garcia@plos.org

------------------------------------------------------------------------

DATA POLICY:

Many thanks for providing the data underlying the graphs shown in the figures. We do require all individual quantitative observations, thus please add or let us know where to find the data shown in the following figures:

Fig. 1B, D, E, F, I; Fig. 2B, C, G, H; Fig. 3B, E, F, G; Fig. 4B, C, D; Fig. 5B, F, G, H, I, M, N; Fig. 6B, D-F, I, J; Fig. 7A, E,F; Fig. S1E, F, G, J; Fig. S2C-E; Fig. S3B, F-M; Fig. S4B; Fig. S5, Fig. S6B and Fig. S7

1) Please also ensure that the data files referred to (in the manuscript, figure legends, and the Description field when uploading your files) using the following format verbatim: S1 Data, S2 Data, etc. Please also save the files using exactly the following convention: S1_Data.xlsx (using an underscore).

2) Please also ensure that figure legends in your manuscript include information on WHERE THE UNDERLYING DATA CAN BE FOUND, and ensure your supplemental data file/s has a legend. And that your Data Statement in the submission system accurately describes where your data can be found.

3) Please note that I can't open the file for Fig. S5, so please upload it again and make sure you include the data underlying all the graphs included in this figure.

4) Please make the data deposited in the ProteomeXchange (PXD059639) publicly available at this stage.

------------------------------------------------------------------------

CODE POLICY

Per journal policy, if you have generated any custom code during the course of this investigation, please make it available without restrictions. Please ensure that the code is sufficiently well documented and reusable, and that your Data Statement in the Editorial Manager submission system accurately describes where your code can be found. More information on our Code Policy, what and how to share can be found here: https://journals.plos.org/plosbiology/s/code-availability

Please note that we cannot accept sole deposition of code in GitHub, as this could be changed after publication. However, you can archive this version of your publicly available GitHub code to Zenodo. Once you do this, it will generate a DOI number, which you will need to provide in the Data Accessibility Statement (you are welcome to also provide the GitHub access information). See the process for doing this here: https://docs.github.com/en/repositories/archiving-a-github-repository/referencing-and-citing-content

# Author comment: Author response to Decision Letter 4

**DOI:** 10.1371/journal.pbio.3003212.r007

7 Jul 2026

Attachment Submitted filename: renamed_bf0be.pdf

# Editor report: Decision Letter 4

**DOI:** 10.1371/journal.pbio.3003212.r008

**Authors:** Ines Alvarez-Garcia

16 Jul 2026

Dear Dr Hornef,

Thank you for the submission of your revised Research Article entitled "Developmental factors drive the compartmentalised and discontinuous maturation of the small intestinal epithelium during the early postnatal period" for publication in PLOS Biology. On behalf of my colleagues and the Academic Editor, Bon-Kyoung Koo, I am delighted to let you know that we can in principle accept your manuscript for publication, provided you address any remaining formatting and reporting issues. These will be detailed in an email you should receive within 2-3 business days from our colleagues in the journal operations team; no action is required from you until then. Please note that we will not be able to formally accept your manuscript and schedule it for publication until you have completed any requested changes.

Please take a minute to log into Editorial Manager at http://www.editorialmanager.com/pbiology/, click the "Update My Information" link at the top of the page, and update your user information to ensure an efficient production process.

PRESS

We frequently collaborate with press offices. If your institution or institutions have a press office, please notify them about your upcoming paper at this point, to enable them to help maximise its impact. If the press office is planning to promote your findings, we would be grateful if they could coordinate with biologypress@plos.org. If you have previously opted in to the early version process, we ask that you notify us immediately of any press plans so that we may opt out on your behalf.

We also ask that you take this opportunity to read our Embargo Policy regarding the discussion, promotion and media coverage of work that is yet to be published by PLOS. As your manuscript is not yet published, it is bound by the conditions of our Embargo Policy. Please be aware that this policy is in place both to ensure that any press coverage of your article is fully substantiated and to provide a direct link between such coverage and the published work. For full details of our Embargo Policy, please visit http://www.plos.org/about/media-inquiries/embargo-policy/.

Many congratulations and thanks again for choosing PLOS Biology for publication and supporting Open Access publishing. We look forward to publishing your study.

Sincerely,

Ines

--

Ines Alvarez-Garcia, PhD

Senior Editor

PLOS Biology

ialvarez-garcia@plos.org
